# FANCM is required for the PAX3::FOXO1-driven oncogenic program in rhabdomyosarcoma

**DOI:** 10.1016/j.celrep.2026.117960

**Published:** 2026-09-08

**Authors:** Christopher D. Delaney, Min Lu, Seth P. Zimmerman, Yingying Meng, Camille Vaganay, Christian G. Cerda-Smith, Annie Liu, Jacob P. Hoj, Shane T. Killarney, Kerry Dillon, Amy E. Stewart, Kevin Huang, Julia D. Caci, Olivia White, Alexandre Puissant, David E. Root, Jack F. Shern, Christine E. Eyler, Chun-Wei David Chen, Christopher M. Counter, Corinne M. Linardic, Lee Zou, Kris C. Wood

**Affiliations:** 1 Department of Pharmacology and Cancer Biology, Duke University School of Medicine, Durham, NC, USA; 2 INSERM UMR 944, IRSL, St Louis Hospital, University of Paris-Cité, Paris, France; 3 Broad Institute of MIT and Harvard, Cambridge, MA, USA; 4 Pediatric Oncology Branch, Center for Cancer Research, National Cancer Institute, Bethesda, MD, USA; 5 Department of Biochemistry and Molecular Biology, Mayo Clinic, 200 First Street S.W., Rochester, MN 55905, USA; 6 Department of Pediatrics, Duke University School of Medicine, Durham, NC, USA; 7 Lead contact

## Abstract

Many cancers are driven by mutationally altered transcription factors (TFs) that rewire cells to an oncogenic state. Cells must activate specific mechanisms to tolerate the burden of oncogenic TF activity. To define such mechanisms, we focused on a canonical oncogenic fusion protein-driven cancer, alveolar rhabdomyosarcoma (ARMS), where the PAX3::FOXO1 fusion protein hyperactivates and mislocalizes PAX3 and FOXO1 TF functions. Employing sequential functional genomic CRISPR-Cas9 screens, we identified FANCM, a DNA translocase in the Fanconi anemia pathway, as a selective dependency in *PAX3::FOXO1+* ARMS. *FANCM* loss reduces fusion protein levels, induces myogenic differentiation, and disrupts the PAX3::FOXO1 transcriptional program, thereby halting oncogenic proliferation. Mechanistically, *FANCM* depletion exacerbates replication stress (RS) and DNA damage signaling, with chromatin-associated RS enriched at PAX3::FOXO1 target gene loci, resulting in selective downregulation of the oncogenic program. CRISPR exon-tiling screens prioritized FANCM’s helicase and DNA-binding domains as essential for this dependency, linking FANCM-mediated replication fork binding to sustained oncogenesis.

## In brief

Delaney et al. show that PAX3::FOXO1 oncofusion-positive rhabdomyosarcoma depends on FANCM to tolerate fusion-associated replication stress. FANCM depletion disrupts the fusion transcriptional program and promotes myogenic differentiation, nominating FANCM as a selective therapeutic target in this rare and aggressive pediatric malignancy.

## Graphical Abstract

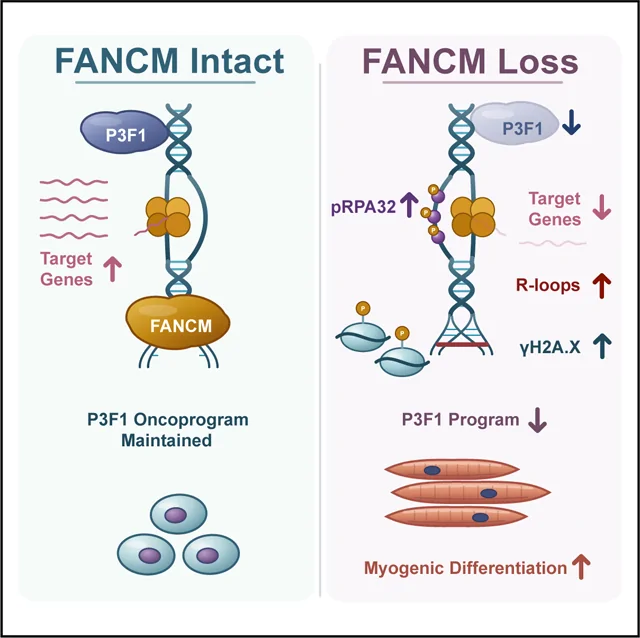

## INTRODUCTION

The dynamic regulation of transcription factor (TF) activity underpins essential cellular processes, including embryonic development, lineage commitment, and proliferation.^1,2^ Accordingly, dysregulated TF activity is a hallmark of oncogenesis, particularly in pediatric cancers driven by fusion oncoproteins.^3^ In the example of alveolar rhabdomyosarcoma (ARMS), the PAX3::FOXO1 fusion protein hyperactivates and mislocalizes PAX3 and FOXO1 TF functions, rewiring myogenic lineage commitment into a proliferative yet myoblastic state.^4,5^ The scale of transcriptional disruption imposed by oncogenic TFs such as PAX3::FOXO1 impairs cell fitness, yet very little is known about the mechanisms used by cells to tolerate this burden.

Rhabdomyosarcoma (RMS) is the most common soft tissue sarcoma in children and adolescents.^6^ The ARMS subtype makes up approximately 30% of all RMS cases.^7,8^ ARMS tumors are molecularly classified as fusion-positive (FP) based on the presence of the PAX3::FOXO1 (P3F1) or PAX7::FOXO1 (P7F1) fusion proteins, which account for approximately 60% and 20% of cases, respectively.^9^ Fusion status is an important clinical indicator as both gene rearrangements are associated with aggressive clinical progression and poor prognosis, particularly P3F1.^10,11^ Five-year survival rates for afflicted children have plateaued around 50% and drop below 20% when distant metastases are present at diagnosis.^12^ While progress has been made to improve survival outcomes in other subtypes of RMS, clinical treatments that translate to improved patient outcomes remain elusive in ARMS. Unfortunately, this treatment intractability is characteristic of many cancers driven by mutated TFs, as the downstream cellular changes are vast and the oncoproteins themselves are difficult to target directly with small molecules.

The PAX3::FOXO1 fusion protein is a potent transcriptional activator that impairs myogenic differentiation and promotes tumorigenesis by driving the expression of a highly coordinated oncogenic transcriptional program.^4,5,13,14^ We hypothesized that this miswired oncogenic transcriptional program in FP RMS would render cells reliant on a specific set of regulators needed to sustain and coordinate oncofusion function, beyond the canonical downstream effector genes of the fusion protein that are prone to resistance when targeted individually. CRISPR-Cas9-based genetic screening has emerged as a powerful tool for unbiased identification of essential genes in cancer, providing an opportunity to uncover genetic vulnerabilities.^15,16^ In this study, we employed a sequential multimodal CRISPR-Cas9 screening approach to identify selective vulnerabilities in FP RMS cells required to maintain the PAX3::FOXO1-driven oncogenic transcriptional program. We performed genome-scale loss-of-function screens in both monolayer and 3D spheroid cultures, followed by targeted sub-library screens across multiple FP and fusion-negative (FN) RMS cell lines. Additionally, we utilized flow cytometry-based functional screens to identify genes regulating PAX3::FOXO1 protein levels and myogenic differentiation. Our comprehensive analysis highlighted *FANCM*, which encodes a key member of the Fanconi anemia (FA) pathway, as a critical dependency required to sustain the oncogenic transcriptional program in FP RMS.

FANCM plays a well-established role in DNA damage repair, particularly in the resolution of replication stress (RS).^17–19^ Oncogene-induced RS is emerging as a hallmark of cancer, particularly in oncofusion-driven pediatric malignancies such as FP RMS and EWS::FLI1-driven Ewing sarcoma.^20–23^ Our findings demonstrate that *FANCM* loss selectively impairs the growth of FP RMS cells both *in vitro* and *in vivo*, reduces PAX3::FOXO1 protein and transcript levels, shuts down the oncogene transcriptional program, and induces myogenic differentiation. Mechanistically, we show that *FANCM* loss increases RS, DNA damage, and downstream DNA damage response (DDR) signaling in FP cells. More specifically, upon *FANCM* loss, chromatin-associated RS is enriched at the *PAX3::FOXO1* locus and other downstream PAX3::FOXO1 target loci, coinciding with reduced fusion protein abundance and suppression of its transcriptional program. Furthermore, CRISPR-Cas9 exon-tiling screens prioritized FANCM’s helicase and DNA-binding domains as candidate functional hotspots for its FP-specific dependency, implicating its role in replication fork binding and fork stability as key functions underlying FANCM’s role in protecting cells from PAX3::FOXO1-induced RS.

This work identifies FANCM as a potential therapeutic target in FP RMS and highlights the power of multimodal CRISPR-Cas9 genetic screens to uncover cancer vulnerabilities. Indeed, interest continues to mount around understanding FANCM biology and druggability given its role in both alternative lengthening of telomeres (ALT) maintenance, a process that sustains tumorigenesis in 10%–15% of human malignancies,^18^ and synthetic lethality with SMARCAL1.^24,25^ This interest is further piqued by the fact that biallelic *FANCM* loss is tolerable in both humans and mice, suggesting the existence of a potentially broad therapeutic window for future FANCM inhibitors.^26,27^ More broadly, this work defines a paradigm whereby oncofusion TF activity can be disrupted by targeting stress tolerance mechanisms engaged at fusion protein target loci, a concept that may be extensible to additional DNA repair vulnerabilities in other oncofusion-driven malignancies.

## RESULTS

### Multimodal CRISPR-Cas9 genetic screens nominate *FANCM* as a genetic dependency and regulator of PAX3::FOXO1 in RMS cell lines

First, we used a genome-scale CRISPR-Cas9 loss-of-function screen to identify genes required for FP RMS cell growth in monolayer and 3D spheroid culture (Figure 1A; Figures S1A and S1B).^28^ Screening was performed in Rh4 clone cells, which harbor an endogenous *PAX3::FOXO1-FLAG* allele,^29^ using the TKOv3 CRISPR-Cas9 library.^30^ Samples from day 0 and day final (13 doublings) were sequenced to identify candidate dependencies. Screen design incorporated low MOI transduction, high coverage, replicate screening, representation maintenance, non-targeting control normalization, and MAGeCK gene-level scoring to mitigate common pooled-screen limitations.^30,31^ The genome-scale screens showed high concordance between monolayer and spheroid essentiality scores (Figure 1B), with modest condition-specific differences (Figure S1C; Table S1). We used lead candidates from these screens to design a 7,501-sgRNA sub-library targeting 1,841 genes, including FP-specific DepMap genes, PAX3::FOXO1 target genes, nonessential genes, and controls (Table S2).^32^ Targeted sub-library essentiality screens were performed in four FP cell lines (Rh4 clone, Rh4 native, Rh28, and Rh30) and two FN cell lines (RD and SMS-CTR) (Figure 1C). Cells were transduced with Cas9 and then with the sgRNA-only sub-library, and day final versus day 0 sgRNA depletion was analyzed to identify genes preferentially required in FP cells.

We identified a set of genes with lower beta scores in FP versus FN cell lines, which are the genes most required to maintain the PAX3::FOXO1-driven oncogenic state (Figure 1D; Table S3). STRING functional protein association network analysis of the top 100 such genes revealed genes associated with the PAX3::FOXO1 transcriptional activation program, as well as members of the FA pathway (Figure S1D).^33^ Strikingly, gene set enrichment analysis (GSEA) of these top 100 FP-specific genes revealed the FA pathway to be the most enriched gene set in the Kyoto Encyclopedia of Genes and Genomes (KEGG) human database (Figure S1E).

We next used the same sub-library in FACS-based screens to identify genes regulating oncogene levels and function. For PAX3::FOXO1 protein levels, we used the Rh4 clone endogenous *PAX3::FOXO1-FLAG* allele and validated anti-FLAG flow cytometry with breakpoint-targeting shRNA (Figures S2A and S2B).^34^ After sub-library transduction, sgRNA enrichment in low-FLAG versus high-FLAG populations identified genes whose loss reduced fusion protein abundance (Figure 1E). The PAX3::FOXO1-FLAG flow-based screen identified regulators of fusion protein levels (Figure 1F). Reassuringly, sgRNAs targeting *PAX3* reduced PAX3::FOXO1 levels in our screen, consistent with on-target editing of the *PAX3*-derived coding sequence retained in the fusion protein. However, these guides are not fusion-selective, because the same target sequences are present in endogenous *PAX3*. Thus, sgPAX3 represents a dual *PAX3/PAX3::FOXO1* perturbation rather than a selective *PAX3::-FOXO1* loss-of-function control. Nevertheless, we identified lead candidate modulators of fusion protein levels that include FA pathway, DDR signaling, cell cycle control, and metabolic pathway genes, as well as regulators of RNA and protein processing (Figure S2C; Table S4).^33^

A parallel FACS-based screen identified regulators of myogenic differentiation. Selective *PAX3::FOXO1* suppression by shP3F1 repressed the fusion program and induced differentiation (Figure S2D). RNA-seq and western blotting nominated CKM as a robust differentiation marker after *PAX3::FOXO1* KD (Figures S2E and S2F) and CKM induction was detectable by flow cytometry (Figure S2G). Four FP cell lines were then screened for sgRNAs enriched in CKM-high versus CKM-low populations (Figure 1G). The CKM screen identified regulators of myogenic differentiation in FP cell lines, including chromatin-remodeling, FA pathway, DDR, cell cycle, metabolic, and RNA/protein-processing genes (Figure 1H; Figure S2H; Table S5). Because sgPAX3 disrupts both endogenous *PAX3* and *PAX3::FOXO1* and dual targeting has been associated with apoptosis rather than differentiation,^35^ sgPAX3 was not used as a fusion-selective CKM-induction benchmark. Hits were instead defined by gene-level enrichment in CKM-high versus CKM-low populations and interpreted alongside the PAX3::FOXO1-FLAG and viability screens.

Combined analysis of the essentiality, PAX3::FOXO1-FLAG and CKM screens nominated *FANCM*, a member of the FA pathway, as the lead concordant candidate. *FANCM* loss was selectively toxic to FP RMS cell lines, reduced fusion protein levels, and induced myogenic differentiation (Figure 1I; Figure S2I; Table S3). Other genes including *ARID4B*, *KIF5B*, and *FRG1* showed discordant phenotypes that may reflect functions beyond fusion-program regulation or technical variability across assay readouts.

### *FANCM* is a dependency in FP RMS models *in vitro* and *in vivo*

To interrogate the role of FANCM in FP RMS, we first evaluated the effect of *FANCM* loss in DepMap across 6 FP cell lines, which validated our screening results by showing a significantly lower gene effect score in FP cell lines compared to all other cancer cell lines tested (Figure 2A).^32^ We validated this *FANCM* dependency in a non-pooled, single sgRNA format across four FP cell lines in both traditional cell culture growth assays (Figure 2B) and clonogenic assays (Figure S3A) using two independent sgRNAs. Both sgRNAs targeting *FANCM* were confirmed to cause population-level KO of FANCM by WB (Figure 2C). To rescue the *FANCM* KO phenotype, we developed a *FANCM* ectopic expression construct with silent mutations to evade sgFANCM-mediated base editing (Figure S3B). Expression of the *FANCM* rescue construct prior to *FANCM* KO by sgRNA reversed the growth defect phenotype in FP RMS cells (Figure 2D) and also the clonogenic defect observed upon *FANCM* KO (Figure S3C).

To test the growth dependency phenotype of FP RMS cells on *FANCM in vivo*, we used an established inducible sgRNA KO system (Figures S4A and S4B).^36^ In xenograft models of FP RMS based on the Rh4 clone cell line, KO of *FANCM* by inducible-sgRNA significantly slowed tumor growth and enhanced mouse survival relative to control sgRNA (Figure 2E). Analysis of tumors that did ultimately grow in the *FANCM* KO xenografts demonstrated reduced frameshift mutation frequency by tracking of indels by decomposition (TIDE) analysis, consistent with the notion that *FANCM* loss was toxic to FP RMS tumors and suggesting positive selection for cells lacking knockout (Figure S4C).^37^ Congruent with these findings, orthogonal approaches to reduce *FANCM* levels by inducible-shRNA demonstrated similar findings *in vitro* (Figures S5A–S5C) and *in vivo* (Figure S5D).^38^

We next tested if *PAX3::FOXO1* expression alone is sufficient to induce sensitivity to *FANCM* loss using a model wherein a Dox-inducible *PAX3::FOXO1* construct is expressed in immortalized human myoblasts.^39^ Upon doxycycline treatment, the fusion protein was detected by WB (Figure 2F). To investigate the effects of concurrent *FANCM* loss and *de novo PAX3::FOXO1* expression, we transduced these cells with an inducible-shRNA targeting *FANCM* or a seed-matched control (SMC) hairpin. Upon doxycycline induction, both the shRNA construct and the ectopic *PAX3::FOXO1* were expressed simultaneously. In this system, induction of *FANCM* KD induced a growth defect only in *PAX3::-FOXO1* expressing cells, suggesting that fusion protein expression alone is sufficient to sensitize cells to *FANCM* loss (Figure 2G).

### *FANCM* loss reduces PAX3::FOXO1 levels and suppresses the fusion transcriptional program

Consistent with the results of the PAX3::FOXO1-FLAG flow-based screens, loss of *FANCM* reduced fusion protein levels (Figure 3A). Similarly, *PAX3::FOXO1* transcript levels were also decreased upon *FANCM* KD, as observed by RT-qPCR (Figure 3B). To test whether this decrease reflected altered mRNA stability, an actinomycin D chase assay was performed after *FANCM* KD. *PAX3::FOXO1* transcript degradation kinetics was not significantly altered compared with SMC, arguing against accelerated transcript decay as the primary mechanism of reduced PAX3::FOXO1 abundance (Figure 3C). These data suggest that *FANCM* loss reduces *PAX3::FOXO1* transcript abundance upstream of mRNA degradation, potentially through altered transcriptional production or co-transcriptional processing.

To obtain a more comprehensive analysis of mRNA transcript-level changes upon *FANCM* loss, we performed RNA sequencing after shRNA KD of *FANCM*. We found a decrease in *PAX3* and *FOXO1* transcript levels (a proxy for *PAX3::FOXO1* levels, despite contamination with endogenous wild-type *PAX3* and *FOXO1* reads) upon *FANCM* KD (Figure 3D). Notably, GSEA of transcript-level changes upon *FANCM* KD revealed multiple PAX3::FOXO1 target gene sets among the most negatively enriched gene sets in the C2:CGP Molecular Signatures Database collection from MSigDB (Figure 3E).^40^ To test whether this suppression of PAX3::FOXO1 target gene expression reflected a generic response to DNA damage, the same gene sets were analyzed after 5 Gy irradiation at 24 h. In contrast to *FANCM* KD, irradiation did not deplete PAX3::FOXO1 target gene sets and instead shifted these gene sets toward positive enrichment, with two gene sets reaching statistical significance for upregulation at 24 h (Figure 3E). This divergent response argues that reduced PAX3::FOXO1 transcriptional output after *FANCM* loss is not simply a general consequence of DNA damage. Indeed, positive enrichment after irradiation may be consistent with prior work showing cell cycle-dependent *PAX3::FOXO1* expression and activity in G2, a phase enriched after DNA damage checkpoint activation following irradiation.^41,42^ Together, these RNA sequencing results support a model in which *FANCM* positively regulates *PAX3::FOXO1* transcript abundance and the downstream fusion oncogene transcriptional program.

### *FANCM* loss induces myogenic differentiation in FP RMS cells

We next sought to characterize the myogenic differentiation phenotype upon shRNA-mediated *FANCM* KD using the same inducible model. Consistent with the CKM flow-based screen results, *FANCM* KD induced morphologic changes consistent with myogenic differentiation and increased cytoplasmic CKM protein by immunofluorescence (Figure 4A). Quantification of mean cytoplasmic CKM intensity confirmed a significant increase in CKM protein after *FANCM* KD compared with non-targeting control (Figure 4B). To define the timing of molecular differentiation markers after *FANCM* loss, Western blotting was performed across days 0, 2, 4, and 6 after doxycycline induction and quantified by densitometry with normalization to both the loading control and day-matched control hairpin (Figure 4C; Figure S6). *FANCM* KD reduced PAX3::FOXO1 protein abundance by day 2, with continued relative decreases on days 4 and 6. MYOD1 protein levels decreased beginning at day 2, while MYOG showed a transient increase at day 2 followed by reduction at later time points. CKM protein was modestly enriched at early time points and increased significantly on day 4, remaining elevated at day 6. Later differentiation-associated structural proteins, including MYL1 and MYH4, increased most prominently at day 6. Because *FANCM* loss also reduces FP RMS cell growth, apoptosis was assessed across the same inducible shRNA-mediated *FANCM* KD time course. Immunoblotting of adherent cells did not show increased cleaved PARP or cleaved caspase-3 after *FANCM* KD compared with SMC, while annexin V and PI staining of total cell populations showed only modest increases in apoptotic fractions (Figures S7A and S7B). Thus, *FANCM* loss promotes myogenic differentiation and loss of proliferative capacity without robust activation of apoptotic pathways in the adherent differentiating cell population. Taken together, these findings show that the myoblast-like oncogenic state characteristic of FP cells, in which myogenic regulatory factors and differentiation markers are discordantly expressed at baseline, shifts toward a more differentiated myogenic state upon *FANCM* loss, characterized by increased CKM, MYL1, and MYH4 protein levels and decreased early myoblastic TFs MYOD1 and MYOG (Figure 4D**)**.

### *FANCM* loss increases replication stress, DNA damage, and transcription-replication conflicts in FP RMS cells

At baseline, FP RMS cells are known to have higher levels of RS than FN cells, and *PAX3::FOXO1* expression alone is sufficient to increase RS.^23^ Given the established role of FANCM in promoting replication fork restart, fork protection, and repair pathway choice at stalled replication forks, we sought to characterize the impact of *FANCM* loss on RS and DNA damage signaling in FP RMS cells.^19,43–46^ Upon shRNA-mediated *FANCM* KD, RS and DNA damage markers increased, as measured by pRPA32-S8 and γH2A.X protein levels (Figure 5A). Quantitative imaging further showed increased nuclear γH2A.X intensity and increased γH2A.X foci per nucleus after *FANCM* loss, consistent with accumulation of DNA damage (Figure 5B; Figure S8). Ectopic expression of sgRNA-resistant *FANCM* rescued the accumulation of pRPA32-S8 and γH2A.X after *FANCM* KO, consistent with on-target regulation of these RS and DNA damage phenotypes by *FANCM* (Figure 5C). *FANCM* rescue also restored FP RMS growth and the expression of selected downstream PAX3::FOXO1 target genes (Figures S9A and S9B).

To characterize downstream DDR signaling after *FANCM* loss, ATR-associated and ATM-associated phospho-signaling markers were assessed across days 0, 2, 4, and 6 after doxycycline induction. *FANCM* KD increased multiple DDR markers across the time course, including pATR-T1989, pATRIP-S224, pCHK1-S317, pCHK1-S345, pATM-S1981, and pCHK2-T68, consistent with activation of checkpoint signaling after RS and DNA damage accumulation (Figure 5D; Figure S10). These data show that *FANCM* loss increases RS and activates downstream DDR signaling in FP RMS cells.

Because transcription-replication conflicts (TRCs) are a major source of RS at highly transcribed loci, TRCs were next assessed by proximity ligation assay (PLA) between PCNA and phospho-RNA polymerase II Ser2. Because PCNA marks replication machinery at active forks and phosphorylation of RNA polymerase II at Ser2 marks elongating transcription complexes, PCNA-phospho-RNA polymerase II Ser2 PLA foci provide a cellular readout of close proximity between replication and transcription machineries, as used previously to assess TRCs.^47^ *FANCM* KD increased PCNA and phospho-RNA polymerase II Ser2 PLA foci, consistent with increased TRCs after *FANCM* loss (Figure 5E). Since RNase H1 hydrolyzes the RNA strand of RNA-DNA hybrids that form R-loops, wild-type RNase H1 provides a functional test of whether R-loop-associated RNA-DNA hybrid structures contribute to TRC-associated phenotypes, while catalytically inactive RNase H1 controls for nuclease-independent effects of RNase H1 expression.^48^ Inducible expression of wild-type RNase H1 reduced TRC-associated PLA foci in *FANCM* KD cells (Figure 5E). To assess R-loop-associated RS, we performed PLA between S9.6, an antibody that recognizes RNA-DNA hybrids including R-loops, and pRPA32-S33, a marker of RS. FANCM KD increased S9.6-pRPA32-S33 PLA foci, and this signal was reduced by wild-type RNase H1 expression (Figure 5F). In parallel assays, wild-type RNase H1, but not catalytically inactive mutant RNase H1, partially rescued the growth defect caused by *FANCM* KD and partially restored expression of selected PAX3::FOXO1-regulated transcripts (Figures S9C and S9D). Together, these data suggest that RNase H1-sensitive R-loops and R-loop-associated TRCs contribute to the RS, growth defect, and suppression of selected PAX3::FOXO1-regulated transcripts observed after *FANCM* loss.

### *FANCM* loss enriches chromatin-associated replication stress at PAX3::FOXO1-regulated loci

Having established that *FANCM* loss increases RS, DNA damage signaling, TRCs, and RNase H1-sensitive R-loop-associated pRPA32-S33 PLA, we next asked where chromatin-associated RS accumulates across the genome. The S9.6-pRPA32-S33 PLA assay supports increased proximity between RNA-DNA hybrid-containing structures and phosphorylated RPA after *FANCM* loss, but this imaging-based assay does not identify the genomic loci experiencing RS. Therefore, pRPA32-S33 ChIP-seq was performed in triplicate after shRNA-mediated *FANCM* KD compared with a SMC in Rh4 Native cells to map the genomic distribution of *FANCM*-loss-induced RS. A recent proximity-labeling interactome study of FP RMS oncofusions identified FANCM among the proteins associated with PAX3::FOXO1, supporting the possibility that this RS could be linked to the fusion oncoprotein-associated chromatin state.^49^ However, because high-quality FANCM ChIP-seq-compatible antibody reagents were unavailable, pRPA32-S33 ChIP-seq served as a locus-resolved readout of RS rather than a direct map of FANCM protein occupancy.

DiffBind analysis identified genomic loci enriched for pRPA32-S33 signal after *FANCM* KD (Figure 6A; Table S6).^50^ Genes associated with increased pRPA32-S33 signal included functional groups involved in nucleosome biology, cytoskeletal and extracellular matrix regulation, NOTCH signaling, transcriptional processing, and chromatin remodeling (Figure 6B).^33^ Remarkably, gene set overrepresentation analysis of pRPA32-S33-enriched genes identified PAX3::FOXO1 target gene sets among the most enriched gene sets (Figure 6C). Thus, the RS signal induced by *FANCM* loss is enriched at loci linked to the fusion oncogenic transcriptional program. At *QKI*, a previously identified PAX3::FOXO1 target gene, pRPA32-S33 signal increased near the transcription start site, distal to nearby PAX3::FOXO1-occupied, H3K27ac-enriched enhancer regions (Figure 6D).^13,14,51^

The *PAX3::FOXO1* locus is a target of PAX3::FOXO1 itself, specifically at the C-terminal translocated *FOXO1* super-enhancer that engages the PAX3 promoter.^4^ Examination of this *FOXO1* locus identified increased pRPA32-S33 signal near the end of the gene, close to but not directly overlapping PAX3::FOXO1 and H3K27ac co-occupied regions (Figure 6E). This proximity without direct peak overlap is consistent with a TRC model in which oncogene-induced RS arises within actively transcribed genes and at genic features such as transcription start and termination regions, such that pRPA32-S33 signal need not coincide precisely with enhancer-bound PAX3::FOXO1 and H3K27ac peaks.^52–54^ Together with the TRC data aforementioned, these locus-resolved pRPA32-S33 ChIP-seq results support a model in which *FANCM* loss increases transcription-linked RS at PAX3::FOXO1-regulated loci, thereby contributing to repression of the fusion oncogenic transcriptional program.

### CRISPR-Cas9 exon-tiling screen prioritizes candidate functional hotspots in FANCM that underlie its FP-specific dependency

We next performed a CRISPR-Cas9 exon-tiling screen across the entire *FANCM* coding region to identify functional regions of FANCM that underlie the FP-selective dependency.^55–57^ The Rh4 clone and RD cell lines were selected for this domain-mapping experiment because they showed the largest differential growth response to *FANCM* loss among the FP and FN models tested, thereby maximizing the phenotypic window for detecting domain-level effects. The exon-tiling library contained 428 sgRNAs saturating every PAM site in *FANCM* exons, approximately every 14 base pairs **(**full details in Table S7). Built-in controls included 200 non-targeting control sgRNAs and select positive control sgRNAs targeting essential genes, including *PAX3*. Cells were transduced with the exon-tiling sgRNA library and grown for 3 weeks. Final time point samples were deep sequenced and deconvoluted to identify those sgRNAs that changed frequency throughout the screen time course. Final sgRNA frequencies were compared in FP versus FN samples, averaged across a sliding window, and standardized to identify those regions most differentially essential in FP cells. Notably, the two N-terminal helicase domains, the adjacent MID domain, as well as the C-terminal ERCC4 nuclease/FAAP24-interacting domain are most differentially essential in the FP RMS cells (Figure 7A). Importantly, these regions map to FANCM activities involved in engagement with branched DNA or stalled-fork-associated repair complexes: the N-terminal translocase domain remodels replication-fork-like DNA structures, the MID/MHF interaction promotes FANCM localization to DNA branch points, and the C-terminal ERCC4/HhH/FAAP24-interacting region contributes to FANCM-associated DNA damage signaling and chromatin engagement (Figure 7B).^44–46,58^

To provide focused validation of selected exon-tiling hotspots, individual sgRNAs targeting the N-terminal helicase region and C-terminal FAAP24-interacting region were tested in non-pooled assays (Figures S11A and S12A). Both hotspot-targeting guides recapitulated the FP-selective growth phenotype observed in the tiling screen, with impaired Rh4 clone colony formation and relative sparing of RD cells (Figures S11B and S12B). Allele-level sequencing across the edited loci identified specific in-frame edited alleles that were depleted over time, including deletions spanning residues 91 to 94 in the N-terminal helicase region and residues 1922 to 1925 in the C-terminal FAAP24-interacting region (Figures S11C and S12C). These depleted residues are evolutionarily conserved and map to structured regions predicted to affect FANCM protein engagement with DNA or FAAP24 (Figures S11D–S11F and S12D–S12F). Together, these focused validation studies support the exon-tiling nomination of the N-terminal helicase and C-terminal FAAP24-interacting regions as candidate functional hotspots, while full engineered mutant-rescue studies will be required to define the precise biochemical contribution of individual residues.

## DISCUSSION

The finding that *FANCM* is required to maintain the PAX3::FOXO1 oncogenic transcriptional program in FP RMS identifies a RS tolerance mechanism that supports fusion TF-driven oncogenesis. The sequential multimodal CRISPR-Cas9 screening approach used here nominated *FANCM* across orthogonal readouts of FP RMS fitness, PAX3::FOXO1 abundance, and myogenic differentiation, linking RS tolerance to maintenance of the FP RMS oncogenic state.

In the baseline oncogenic state, PAX3::FOXO1 drives high levels of RS.^23^ This is consistent with localized pRPA32-S33 accumulation at baseline at PAX3::FOXO1 target genes, including near the *PAX3::FOXO1* locus (Figures 6D and 6E, light blue tracks). This RS may be driven, at least in part, by R-loop-associated TRCs at highly transcribed oncogene-regulated loci, a mechanism described for oncogene-induced and fusion-oncogene-associated RS.^20,52^ With FANCM present, FP RMS cells appear able to tolerate enough fusion-associated RS to successfully complete replication and division, while maintaining an oncofusion transcriptional program that blocks myogenic differentiation and coordinates proliferation (Figure 7C). The observation that *PAX3::-FOXO1* transcript and protein levels are repressed in S phase may be consistent with this model, suggesting that cell cycle-linked dampening of fusion activity could help limit self-inflicted RS during DNA replication.^41^ That mutated and hyperactive TF activity imbues cells with a fitness defect inherent to the S phase is intuitive in retrospect, as the complex mechanisms of DNA duplication run against genome-level chromatin changes that support oncogenesis.

Mechanistically, the data support a model in which PAX3::FOXO1-driven transcription creates a focal RS burden that is buffered by FANCM protein. Upon *FANCM* loss, global TRCs and R-loop-associated pRPA32-S33 PLA foci increase, pRPA32-S33 accumulates at PAX3::FOXO1-regulated loci, and downstream DDR markers are activated. These events are accompanied by reduced *PAX3::FOXO1* transcript abundance, reduced PAX3::FOXO1 protein abundance, and suppression of the downstream fusion oncogenic transcriptional program (Figure 7D). Accordingly, the actinomycin D chase assay argues against altered *PAX3::FOXO1* mRNA stability as the primary mechanism of fusion transcript loss, but future nascent transcription studies will be required to distinguish whether *FANCM* loss affects transcription initiation, RNA polymerase II pause release, elongation, or co-transcriptional processing at PAX3::FOXO1 target loci in the context of increased focal RS.

FANCM has emerged as an attractive therapeutic target given its proposed function in alternative lengthening of telomeres (ALT) maintenance in a subset of cancer types.^18^ Our work extends FANCM dependency biology beyond ALT-associated contexts and SMARCAL1-synthetic-lethal settings to a fusion-oncogene-driven FP RMS context.^18,24,25^ Prior work in FP RMS has established the biomarker potential of elevated FANCD2, a downstream effector of the FA core complex, in predicting poor response to radiation therapy, presumably from a heightened ability to resolve treatment-associated DNA damage.^59^ Our findings, however, implicate an FA pathway member as a vulnerability in FP RMS. The therapeutic potential of targeting FANCM is promising, albeit limited by the current lack of specific FANCM inhibitors.^60,61^ The one commercially available inhibitor of FANCM, PIP-199, has been characterized as highly promiscuous and not recommended for biological studies.^62^ Moreover, PIP-199 and related compounds in development target the MM2 domain-RMI1/RMI2 interaction, a functional node of FANCM signaling not strongly implicated in the exon-tiling screen (Figures 7A and 7B).^63^ Consistent with these limitations, dose-response testing of PIP-199 across four FP RMS cell lines and two FN RMS cell lines showed overlapping GI50 values and no reproducible FP-selective activity (Figure S13). Despite these current chemical limitations, it is encouraging that normal human and mouse tissues tolerate biallelic *FANCM* loss, a finding consistent with the observation that cell lines from most other cancer types exhibit similar tolerance to *FANCM* loss in DepMap.^26,27,32^ This contrasts with *ATR*, which is relatively pan-essential across cell types, and suggests a broader therapeutic window for future FANCM-directed interventions.^32^ Consistent with this therapeutic concept, ATR inhibition has already been proposed in FP RMS, although the therapeutic window is limited, including in combination strategies with PARP inhibition.^23^ In exploratory dose-response assays, *FANCM* KD modestly sensitized Rh4 native cells to two clinically relevant ATR inhibitors, AZD6738 (Ceralasertib) and BAY1895344 (Elimusertib), as reflected by a leftward shift in GI50 values (Figure S14). These data support future evaluation of combinations that pair FANCM inhibition with clinically viable DDR inhibitors, while emphasizing the need for FANCM inhibitors that faithfully engage the functional domains nominated here. Further investigation is warranted to study FANCM inhibitors designed against the helicase, MID, and ERCC4 nuclease/FAAP24-interacting domains. The focused hotspot validation (Figures S11 and S12) supports at least two of these domains as priority regions for future study, but detailed engineered mutant-rescue experiments will be needed to define how individual residues affect FANCM protein stability, DNA binding, FAAP24 interaction, checkpoint signaling, and FP RMS viability.

Several additional future directions follow from the presented work. Preliminary non-pooled sgRNA studies showed partial FP RMS growth defects after perturbation of *FAAP24*, the FANCM-interacting partner linked to the C-terminal exon-tiling signal, and *UBE2T*, although whether these genes also regulate RS, PAX3::FOXO1 protein abundance, or myogenic differentiation remains to be determined (Figure S15). For future FANCM inhibitors, induction of myogenic differentiation markers, including CKM, MYL1, and MYH4, together with reduced PAX3::FOXO1 protein abundance or reduced *PAX3::-FOXO1* target gene expression, may provide useful pharmacodynamic biomarkers of on-target FANCM inhibitory activity in FP RMS. Beyond these cell-intrinsic biomarkers, future studies should determine whether exacerbating PAX3::FOXO1-associated RS through FANCM inhibition alters tumor immune recognition or promotes broader antitumor immune responses, particularly in immune-competent FP RMS models.

Overall, this work shows that FP RMS cells tolerate PAX3::FOXO1-driven transcriptional rewiring by engaging FANCM-dependent RS tolerance, and that disrupting this tolerance mechanism collapses the fusion oncogenic program rather than simply producing nonspecific DNA damage. More broadly, oncogenes that rewire chromatin, transcription, or replication programs may create compensatory dependencies on distinct fork-stability and DNA damage-buffering pathways. Defining these stress-tolerance adaptations may reveal therapeutic opportunities to selectively destabilize otherwise difficult-to-target oncogenic states across fusion TF driven and other oncogene-driven cancers.

### Limitations of the study

This study has several limitations. Most mechanistic experiments were performed in established FP RMS cell-line models, and the *in vivo* analyses used immunocompromised xenografts. Therefore, additional patient-derived and immune-competent models will be needed to define the breadth of the FANCM dependency, the tumor microenvironmental consequences of *FANCM* loss, and the possible therapeutic windows. As noted, the CRISPR-Cas9 screens can be influenced by sgRNA efficiency, off-target editing, editing-outcome heterogeneity, and model-specific fitness effects. However, multiple controls, orthogonal perturbation methods, rescue experiments, and validations across multiple cell lines were used to mitigate these limitations. Full structure-function mutant-rescue studies will be required to assign precise biochemical functions to individual FANCM residues or domains. Additionally, the chromatin and PLA assays support increased TRCs, R-loop-associated RS, and pRPA32-S33 enrichment at PAX3::FOXO1-regulated loci after *FANCM* loss, but they do not directly measure nascent transcription or prove the exact step at which transcription is impaired. Finally, selective and well-validated FANCM inhibitors are not yet available, so the therapeutic implications of FANCM inhibition in FP RMS remain to be tested with future pharmacologic tools and more clinically representative models.

## RESOURCE AVAILABILITY

### Lead contact

Further information and requests for resources and reagents should be directed to and will be fulfilled by the lead contact, Kris C. Wood (kris.wood@duke.edu).

### Materials availability

Cell lines and derivative cell lines generated in this study are available from the lead contact upon reasonable request and completion of an institutional material transfer agreement. This study does not include deposition of newly generated plasmids to a public plasmid repository. Plasmids obtained from public repositories or other laboratories are listed in the key resources table and described below.

### Data and code availability

- Raw and processed CRISPR-Cas9 screening data have been deposited at GEO: GSE292882. Raw and processed RNA-seq data have been deposited at GEO: GSE292872. Raw and processed ChIP-seq data have been deposited at GEO: GSE292876. Additional data reported in this paper will be shared by the lead contact upon request.
- This paper does not report original code.
- Any additional information required to reanalyze the data reported in this paper are available from the lead contact upon request.

## STAR★METHODS

### EXPERIMENTAL MODEL AND STUDY PARTICIPANT DETAILS

#### Human cell lines and culture conditions

##### Adherent cell culture

All cell lines were maintained in a humidified incubator at 37°C with 5% CO_2_. All RMS cell lines were kindly provided by Corinne Linardic, including the Rh4 Clone,^29^ Rh4 Native, Rh28, Rh30, RD, and SMS-CTR cell lines. All RMS lines (and their derivatives) were grown in RPMI 1640 (Gibco) medium with 10% fetal bovine serum (FBS, VWR) and 1% penicillin-streptomycin (P/S, Gibco). FBS was switched to tetracycline-free (tet-free) FBS (Cytiva) for use in doxycycline-inducible experiments (ishRNA, isgRNA, and iP3F1). The immortalized human myoblast cell lines (Dbt cell lines) were from Corinne Linardic and were cultured in F10 Medium (Gibco) with 15% FBS (VWR), 1% P/S (Gibco), 0.25 μg/mL Amphotericin B (Gibco), 1 mM creatine (Sigma), 1 mM sodium pyruvate (Gibco), and 50 μg/mL Uridine (Sigma). The 293T AAVpro cell line was purchased from Takara Bio (#632273) and cultured in DMEM high-glucose medium (Gibco) with 10% FBS, 1% P/S, 1% sodium pyruvate (Gibco), 1% non-essential amino acids (Gibco), and 1% GlutaMax (Gibco). Trypsin (0.25%) from Cytiva was used for adherent cell dissociation by incubation at 37°C for 5 min. Each cell line had short tandem repeat (STR) profiling performed at the Duke University DNA Analysis Facility to confirm its identity. Regular *Mycoplasma* testing with the MycoAlert Mycoplasma Detection Kit (Lonza) ensured all the cell lines were free from contamination.

##### Spheroid culture

Cell lines were grown in 3D spheroid culture as previously described.^28^ Briefly, cells were seeded in standard tissue culture medium with 0.75% methylcellulose (Fisher). RPMI 1640 was first mixed with methylcellulose and autoclaved to dissolve, then FBS and P/S were added once cooled to 37°C. Single-cell suspensions of detached adherent cells were seeded at 50,000 cells/cm^2^ in pre-treated ultra-low attachment plates (Corning) for small-scale studies, or self-prepared poly-HEMA-coated (Sigma) tissue culture plates for 3D screening. Accutase (Innovative Cell Technologies) was used to dissociate spheroids for reseeding.

#### Mouse xenograft models

#### Study approval

The Institutional Animal Care and Use Committee (IACUC) at Duke University approved all animal procedures and studies. The IACUC protocol number is A189–22–11.

##### NSG mice

Six-week-old female, NOD.Cg-Prkdc^scid^ Il2rg^tm1Wjl^/SzJ, strain #:005557 mice from the Jackson Laboratory were used for xenograft studies as described below.

### METHOD DETAILS

#### Chemical and drug treatments

##### In vitro

Doxycycline (Dox) hyclate (VWR) was prepared at 2 mg/mL in molecular-grade water and used at the indicated concentrations. Puromycin dihydrochloride (GoldBio), blasticidin S HCl (Gibco), and G418 sulfate (Gibco) were used for selection at 2 μg/mL, 10 μg/mL, and 800 μg/mL, respectively, unless otherwise indicated. Methotrexate (MedChemExpress, DMSO, 0.1 μM), etoposide (MedChemExpress, DMSO, 10 μM), and actinomycin D (Thermo Fisher Scientific/Gibco, DMSO, 50 μM) were used for γH2A.X and transcript-stability assays as indicated. PIP-199 (MedChemExpress, DMSO), AZD6738 (Ceralasertib, MedChemExpress, DMSO), and BAY1895344 (Elimusertib, MedChemExpress, DMSO) were used for dose-response studies as indicated.

##### In vivo

Doxycycline (Dox) hyclate 98% (Thermo Fisher Scientific) was given via daily oral gavage at 1 mg per mouse as indicated to initiate *in vivo* inducible construct expression. Rodent diet chow with 625 mg/kg Doxycycline (Dox) hyclate (Inotiv) was provided *ad libitum* for inducible construct expression maintenance.

#### Plasmids, cloning, and lentiviral production

The following plasmids were purchased from Addgene: Toronto KnockOut (TKO) CRISPR Library - Version 3 (TKOv3; RRID: Addgene_90294), lentiGuide-Puro (RRID: Addgene_52953), lentiCas9-Blast (RRID: Addgene_52962), Tet-pLKOpuro (RRID: Addgene_21915), pRDA_355 (RRID: Addgene_187159), psPAX2 (RRID: Addgene_12260), pMD2.G (RRID: Addgene_12259), and pLenti CMV *Neo* DEST (705–1) (pLenti-CMV-Neo; RRID: Addgene_17392). The pLV-U6-gRNA-hUbC-mCherry-P2A-PuroR plasmid (termed enh-sgRNA-mCherry-Puro) was kindly provided by Charles Gersbach (Duke University). The ectopic *FANCM* rescue construct (Figure S3B; Table S8) was synthesized by GenScript into the pUC57-Mini plasmid, which was then used as the entry clone vector in Gateway cloning. Doxycycline-inducible GFP-tagged RNase H1-WT and RNase H1-D210N expression constructs in the pInducer20 lentiviral backbone, named pinducer-20-RH1WT and pinducer-20-RH1D210N, were provided by the laboratory of Lee Zou.

##### sgRNA constructs

Individual sgRNA constructs were cloned as previously described, with slight modification.^15,75^ In brief, unique 19 or 20-mer sgRNA inserts targeting genes of interest were synthesized by Integrated DNA Technologies (IDT) with flanking sequence adaptors. The synthetic oligos were diluted to 10 μM in molecular grade water and forward/reverse oligo pairs were first phosphorylated with T4 polynucleotide kinase (NEB) and annealed in the thermocycler. Annealed oligos were then purified with the QIAquick PCR Purification Kit (Qiagen) or the DNA Clean and Concentrator Kit (Zymo Research) and diluted 1:200. Diluted annealed oligos were ligated into digested backbone with a T4 ligase reaction (NEB). For individual sgRNA constructs, the backbones were either enh-sgRNA-mCherry-Puro (for constitutive sgRNAs) or pRDA_355 (for the inducible sgRNAs). Both backbone constructs were prepared for ligation the same way: restriction enzyme digestion with BsmBI-v2 (NEB) and concurrent dephosphorylation with Quick CIP (NEB), followed by gel purification from a 1% agarose gel using the QIAquick Gel Extraction Kit (Qiagen). Ligation reaction mixture was transformed into One Shot Stbl3 Chemically Competent *E. coli* (Invitrogen). Transformed cells were recovered and spread on LB-ampicillin plates for overnight incubation. Single colonies were picked, cultured overnight in liquid LB broth, and plasmid was extracted and purified using the QIAprep Spin Miniprep Kit (Qiagen) or the ZymoPURE Plasmid Miniprep Kit (Zymo Research). Plasmid DNA sequences were checked using Sanger sequencing (Eton Bioscience) with the hU6 promoter primer (LKO.1 5’: GACTATCATATGCTTACCGT). Most sgRNA targeting sequences were from the TKOv3 library and were the same in the constitutive or inducible systems: sgLuc: TCTAGAGGATAGAATGGCGC, sgLacZ: CTGACAATGGCAGATCCCAG, sgPAX3: ATTCGGGAA AGGTGAAGAGG, sgFANCM-1: GAAATTGTACATGACCACGG, sgFANCM-2: TACCCAGAAGGAGCAACTGG. The targeting sequence for sgNTC was from the MinLibCas9 library (sgRNA ID: CTRL00147) with sequence: TATGCTCTATGGGATAAAC.^76^

##### shRNA constructs

Individual shRNA constructs were cloned into the doxycycline-inducible Tet-pLKOpuro backbone as previously described.^15^ The targeting sequence against *FANCM* and the mammalian non-targeting control sequence was from The RNAi Consortium (TRC): ishFANCM: GCTCTCGGAAACTATGCTTAT (TRCN0000051357), and ishNTC: CAACAAGATGAAGAGCACCAA (SHC002). Bases 9–13 on ishFANCM were mutated to make the seed-matched control (SMC): ishFANCM-SMC: GCTCTCGGTTTGAATGCTTAT. The *PAX3::FOXO1* breakpoint-targeting sequence was modified from prior work: ishP3F1: CCTCTCACCTCAGAATTCATT.^34^

##### FANCM rescue construct

*FANCM* ectopic rescue ORF (Figure S3B; Table S8) was synthesized and cloned into pLenti-CMV-Neo using a Gateway cloning strategy.^77^ Briefly, *FANCM* consensus coding sequence CCDS32070 was modified with silent point mutations to ablate sgRNA binding from five sgRNAs, as well as three shRNAs. A 5^′^ Kozak sequence was added for enhanced mammalian expression. A C-terminal HA-tag connected by a GGGGS-GGGGS-GGGGS linker was added for facile detection. Flanking attL1 and attL2 sites were included for Gateway cloning. This modified *FANCM* ORF sequence was synthesized by GenScript into the pUC57-Mini plasmid, which was then used as the entry vector in Gateway cloning. Final expression construct (pLenti-CMV-FANCM-silently-mutated-HA-Neo, simply termed Ectopic *FANCM* Rescue Construct) was obtained via Gateway LR Clonase II (Invitrogen) reaction between entry clone and destination vector. Purified plasmid sequence was confirmed by Sanger sequencing across the entire *FANCM* ORF. Sequence-checked plasmid was used to make lentivirus, which was transduced into Rh4 Native cells, selected for construct integration with G418 at 800 μg/mL for more than 2 weeks, and then transduced with indicated sgRNA constructs. Knockout-resistant FANCM-HA expression was confirmed by immunoblotting.

##### RNase H1 constructs

The pinducer-20-RH1WT and pinducer-20-RH1D210N plasmids were provided by the laboratory of Lee Zou. Sequence-checked plasmids were used to make lentivirus, transduced into Rh4 Native cells, selected for construct integration with G418 at 800 μg/mL for more than 2 weeks and used for rescue experiments.

##### Lentivirus production

Lentivirus was produced as previously described, with minor changes.^15^ Briefly, 293T AAVpro cells were grown to 50–60% confluency and transfected using Lipofectamine 3000 (Invitrogen) or Lipofectamine LTX (Invitrogen) with PLUS Reagent (Invitrogen). Plasmid DNA from psPAX2, pMD2.G, and the lentiviral construct were first diluted in Opti-MEM medium (Gibco). PLUS Reagent was then diluted in Opti-MEM and gently pipetted into the DNA mixture. After a 5-min room temperature incubation, Lipofectamine 3000 (or LTX) diluted in Opti-MEM medium was pipetted into the DNA/PLUS Reagent mixture. After a 15-min room temperature incubation, the mixture was added dropwise onto 293T AAVpro cells. After incubating the cells at 37°C for 8–12 h in transfection mixture, transfection medium was aspirated and exchanged for virus harvest medium (293T medium with 30% FBS and no P/S). After 48 h, virus-containing medium was harvested, filtered with a 0.45 μm filter and stored at – 80°C.

##### Transduction

Cells were transduced as previously described, with minor differences.^78^ Briefly, cells were spinfected with a transduction mixture of lentivirus-containing medium, 8 μg/mL polybrene, and standard medium at 2,000 rpm for one hour at 37°C. The following day, fresh medium with 2 μg/mL puromycin, 10 μg/mL blasticidin, or 800 μg/mL G418 was added as indicated. Cells were maintained in selection antibiotic for 3–4 days (puromycin), at least 10 days (blasticidin), or greater than 2 weeks (G418) to ensure adequate selection. For large-scale transductions of sgRNA libraries, up to 5 million cells/well in a 6-well plate were transduced by spinfection and then were transferred to 15-cm dishes the next day upon initiating puromycin selection.

#### CRISPR/Cas9 genetic screens

##### Genome-scale screens

The TKOv3 plasmid library was amplified according to published Addgene protocols.^30^ The TKOv3 library contains 70,948 sgRNAs targeting 18,053 protein-coding genes (∼4 sgRNAs targeting each gene) and 142 non-targeting control sgRNAs against LacZ, EGFP and luciferase. Briefly, TKOv3 pooled plasmid library DNA was diluted 1:10 in TE buffer (Invitrogen) and electroporated into Endura electrocompetent cells (Lucigen) at a coverage of >25× of the library. The library plasmid pool was purified using the Plasmid Plus Maxi Kit (Qiagen). Lentivirus generation and transduction were performed as described at a minimum of 500× coverage. Viral titration was performed as previously described.^15^ Briefly, viral titer was found by transducing Rh4 Clone Cells with a 1:2 dilution series of library virus. Cells were selected with puromycin for 3 days and the multiplicity of infection (MOI) was determined by the number of cells infected. Rh4 Clone cells were transduced at an MOI of ∼0.3 (for an average of one sgRNA integration per cell) at >500× coverage of the library and then were puromycin selected for 4 days. Cells were then divided into 2D and 3D conditions in duplicate and initial time point (Time Initial) samples were harvested. Cells were passaged and harvested for cell pellets (40 million cells/pellet/replicate) every 4 days, and were reseeded at sufficient numbers to maintain >500× coverage. Genomic DNA was extracted from cell pellets using the DNeasy Blood & Tissue Kit (Qiagen) for Time Initial pellets and Time Final pellets (day 30 for 3D culture and day 21 for 2D culture), when doubling times were equal at ∼13 population doublings. Pooled plasmid library DNA was used as a template for Plasmid Library sample at sufficient coverage to maintain >500× coverage. Amplification of the sgRNA barcodes and indexing of each sample was performed via 2-step PCR as detailed below.

##### Custom library generation

The custom sgRNA library was designed with 7501 sgRNAs targeting 1841 genes, including top candidates from the genome-scale screen, FP-specific genes from DepMap, PAX3::FOXO1 target genes, and a robust list of nonessential and control genes **(full details in** Table S2). Nonessential and control genes totaled 2590 sgRNAs, roughly 35% of the library, to ballast the predicted depletion of most other sgRNAs due to toxicity. This nonessential subset of the library was termed the Foundation library. sgRNA targeting sequences were primarily from TKOv3, but supplemented with custom-designed sequences from CRISPick for a minority of genes not found in the TKOv3 library.^30,79^ The oligo pool was synthesized by Twist Bioscience. Briefly, a multiplex strategy was employed in the synthesized oligo pool following the CRISPR Clue pipeline to separately amplify the Foundation library of control genes and the curated FP-relevant genes.^80^ This design strategy (separately amplifying the Foundation library and FP-relevant gene library from a multiplexed oligo pool, followed by combining the two libraries in a molar-weighted ratio based on the number of sgRNAs per library) allowed for reuse of the Foundation library in the development of other custom libraries, as well as for economies of scale in oligo pool synthesis (since two additional sgRNA libraries not used in this study were included in the multiplexed oligo pool). The library made from combining the Foundation and FP-relevant libraries was termed the ARMS library. Library amplification was performed as described in the CRISPR Clue protocol and library cloning into the Lenti-Guide-Puro backbone was performed as previously described using blunt-end ligation (NEB).^80–82^ Library coverage was maintained at >200× during cloning and the separate multiplexed libraries (Foundation + FP-relevant libraries) were mixed together following cloning. NGS was performed to validate library composition and confirm minuscule sgRNA contamination from multiplexed libraries not included in the desired custom library design. Lentiviral sgRNA custom library was produced as previously described at >1000× coverage and used below in the indicated essentiality and flow-based screens.

##### Custom library essentiality screens

Custom sgRNA lentiviral library titration was performed as previously described on cell lines stably expressing Cas9 via the LentiCas9-Blast construct.^15^ Briefly, viral titer was found by transducing Rh4 Clone, Rh4 Native, Rh28, Rh30, RD, and SMS-CTR cells with a 1:2 dilution series of library virus. Cells were selected with puromycin for 3 days and the MOI was determined by the number of cells infected. All cell lines were transduced at an MOI of ∼0.3 at >1000× coverage of the library and then were puromycin selected for 4 days. Cells were then divided in duplicate and initial time point (Time Initial) samples were harvested. Cells were passaged and harvested for cell pellets (7.5 million cells/pellet/replicate) every 4 days, and were reseeded at sufficient numbers to maintain >1000× coverage. Genomic DNA was extracted from cell pellets using the *Quick*-DNA Midiprep Plus Kit (Zymo Research) for Time Initial pellets and Time Final pellets (after 21 days post puromycin selection, or ∼13 population doublings). Custom sgRNA plasmid library was used as a template for Plasmid Library sample at sufficient coverage to maintain >1000× coverage. Amplification of the sgRNA barcodes and indexing of each sample was performed via 2-step PCR as detailed below.

##### Custom library PAX3::FOXO1-FLAG flow-based screen

Custom sgRNA lentiviral library titration was performed as previously described on the Rh4 Clone cell line (with a C-terminal FLAG tag knocked-in to the *PAX3::FOXO1* locus).^15,29^ Cells were first made to stably express Cas9 via the LentiCas9-Blast construct. Viral titer was found by transducing the Rh4 Clone cells with a 1:2 dilution series of library virus. Cells were selected with puromycin for 3 days and the MOI was determined by the number of cells infected. Cells were transduced at an MOI of ∼0.3 at >1000× coverage of the library and then were puromycin selected for 4 days. Cells were then divided in triplicate and grown for three additional days until day 7 post transduction. FACS-based screening was then performed as previously described, with slight modifications.^82^ Replicates were harvested, fixed and permeabilized using the eBioscience Intracellular Staining Buffer Set (Thermo Fisher Scientific) according to manufacturer recommendations. Fixed and permeabilized cells were incubated overnight at 4°C with an anti-FLAG antibody (M2, Sigma #F3165) at a 1:100 dilution. The next day, cells were incubated with an anti-mouse-Alexa-647 (Thermo Fisher Scientific) antibody at a 1:100 dilution, then washed and filtered for flow cytometry. For each replicate, stained cells were then sorted by FACS and the top and bottom 10% of FLAG-expressing cells were collected on the basis of A647 signal. Cells were collected in FBS-coated tubes, maintaining greater than 100× coverage per high and low populations. An ungated control (ALL) sample was also collected for each replicate. Immediately following collection, samples were individually subjected to reverse-crosslinking and genomic DNA extraction using the Arcturus PicoPure DNA Extraction Kit (Thermo Fisher Scientific), according to manufacturer recommendations. Amplification of the sgRNA barcodes and indexing of each sample was performed via 2-step PCR as detailed below.

##### Custom library CKM flow-based screen

Custom sgRNA lentiviral titration was performed on Rh4 Clone, Rh4 Native, Rh28, and Rh30 cells transduced with lentiCas9-Blast as described above. All cell lines were transduced at an MOI of ∼0.3 at >1000× coverage of the library and then were puromycin selected for 4 days. Cells were then divided in duplicate and grown for nine additional days until day 13 post transduction. FACS-based screening was then performed as previously described, with slight modifications.^82^ Replicates were harvested, fixed and permeabilized using the eBioscience Intracellular Staining Buffer Set (Thermo Fisher Scientific) according to manufacturer recommendations. Fixed and permeabilized cells were incubated overnight at 4°C with an anti-CKM antibody (1E3, Abnova #H00001158-M02) at a 1:100 dilution. The next day, cells were incubated with anti-mouse-Alexa-647 (Thermo Fisher Scientific) antibody at a 1:100 dilution, then washed and filtered for flow cytometry. For each replicate, stained cells were then sorted by FACS and the top and bottom 10% of CKM-expressing cells were collected on the basis of A647 signal. Cells were collected in FBS-coated tubes, maintaining greater than 100× coverage per high and low populations. An ungated control (ALL) sample was also collected for each replicate. Immediately following collection, samples were individually subjected to reverse-crosslinking and genomic DNA extraction using the Arcturus PicoPure DNA Extraction Kit (Thermo Fisher Scientific), according to manufacturer recommendations. Amplification of the sgRNA barcodes and indexing of each sample was performed via 2-step PCR as detailed below.

##### FANCM exon tiling screen

Another custom sgRNA library saturating the *FANCM* coding sequence was designed and cloned into the enh-sgRNA-mCherry-Puro backbone using a similar sgRNA cloning strategy described above, although without library multiplexing. The custom *FANCM* exon-tiling library contained 999 sgRNAs, with 428 sgRNAs saturating every PAM site in *FANCM* exons (approximately every 14 base pairs on average), 200 non-targeting control sgRNAs, and 371 sgRNAs targeting select positive control and DDR genes (full library composition details in Table S7). *FANCM*-exon-targeting sgRNA sequences were from CRISPick and included all possible *FANCM*-targeting sequences, except when the same genomic site was targeted by both 3^′^ and 5^′^ sgRNAs, wherein only the sgRNA with the best on-target specificity score was included.^79^ Additional sgRNA sequences included in the library were from the MinLibCas9 and TKOv3 libraries, as indicated.^30,76^ Two cell lines, one FP RMS line (Rh4 Clone) and one FN line (RD) were used and were first transduced with Cas9 via the lentiCas9-Blast construct. Briefly, exon-tiling sgRNA lentiviral library titer was found by transducing each cell line with a 1:2 dilution series of library virus. Cells were selected with puromycin for 3 days and the MOI was determined by the number of cells infected. All cell lines were transduced at an MOI of ∼0.3 at >1000× coverage of the library and then were puromycin selected for 4 days. Cells were then divided in triplicate and initial time point (Time Initial) samples were harvested. Cells were passaged and harvested for cell pellets (1 million cells/pellet/replicate) every 4 days, and were reseeded at sufficient numbers to maintain >1000× coverage. Genomic DNA was extracted from cell pellets using the *Quick*-DNA Midiprep Plus Kit (Zymo Research) for Time Initial pellets and Time Final pellets (after 21 days post puromycin selection, or ∼13 population doublings). Custom exon-tiling sgRNA plasmid library was used as a template for Plasmid Library sample at sufficient coverage to maintain >1000× coverage. Amplification of the sgRNA barcodes and indexing of each sample was performed via 2-step PCR as detailed below.

##### CRISPR screen sequencing and analysis

Amplification of the sgRNA barcodes and indexing of each sample was performed via 2-step PCR as previously described using the NEBNext Ultra II Q5 Master Mix (NEB).^15^ Amplified libraries were run on a 2% agarose gel and purified by QIAquick Gel Extraction Kit (Qiagen). Each DNA sample was quantified using the Quant-iT dsDNA High Sensitivity Assay Kit (Thermo Fisher Scientific).

All sequencing was performed at the Duke University Sequencing and Genomic Technologies Core Facility using previously described indexing and sequencing primers from Illumina.^15^ Genome-scale screen samples were pooled and sequenced on a NovaSeq 6000 (Illumina) platform, with 50 bp single-end sequences reported. Custom library essentiality and flow-based screens were pooled and sequenced on a NovaSeq 6000 (Illumina) S-Prime flow cell with 50 bp paired-end sequencing. The FANCM sgRNA tiling screen samples were pooled and sequenced on a NextSeq 1000 (Illumina) P1 flow cell with 50 bp paired-end sequencing.

Raw FASTQ sequencing files were first quality tested with FastQC (RRID: SCR_014583). Read files were then merged across flow cell lanes when applicable. The MAGeCK Count function from the MAGeCK pipeline (RRID: SCR_025016) was used to map sequences to sgRNA read counts.^31^ In all MAGeCK analyses, read counts were normalized to a defined list of non-targeting control sgRNAs unique to each library using the Control normalization method. To obtain Beta (essentiality) scores, the MAGeCK maximum likelihood estimation (MLE) function from the MAGeCK VISPR pipeline was used.^73,83^

For the Genome-scale screens, 2D and 3D Beta scores were obtained from MAGeCK MLE using Time Final samples compared to Plasmid Library samples. For the custom library essentiality screens, the four FP cell lines were merged and Beta scores were obtained from MAGeCK MLE using Time Final samples compared to Plasmid Library samples. The same analysis was performed separately for the two FN cell lines. For the *PAX3::FOXO1*-based flow screen, the P3F1 Depletion Score was equal to the Beta score from MAGeCK MLE using the sgRNA frequencies from bottom 10% population compared to the top 10% population. For the *CKM*-based flow screen, the four FP cell lines were merged and the CKM Depletion Score was equal to the Beta score from MAGeCK MLE using the sgRNA frequencies from bottom 10% population compared to the top 10% population. For the *FANCM* exon tiling screen, control normalized sgRNA counts were obtained from MAGeCK Count for the Rh4 Clone and RD cell lines and the Log_10_–Fold Change (LFC) of Rh4 Clone/RD normalized read counts was smoothed with a 0.1-weighted moving average in JMP Pro Version 17.0.0. This value was then standardized to compare relative depletion and plotted as Standardized Relative sgRNA Depletion (FP vs. FN Cells).

##### Screen design and quality-control considerations

Pooled CRISPR/Cas9 screens were designed and interpreted using low-MOI transduction, high library coverage, replicate screening, maintenance of sgRNA representation during passaging and sorting, non-targeting control normalization, and MAGeCK gene-level scoring across independent sgRNAs. These features were used to mitigate variable sgRNA efficiency, off-target activity, copy-number-associated cutting toxicity, and context-dependent dropout of cells with strong fitness defects.^79,84–86^

#### CRISPR/Cas9 knockout (KO) and shRNA knockdown studies

##### Constitutively active sgRNA system

For constitutive sgRNA-mediated gene KO, a two-part Cas9 and sgRNA system was used. Cells were first transduced with Cas9 via lentiCas9-Blast (RRID: Addgene_52962) and were selected with blasticidin at 10 μg/mL for at least 10 days. Blasticidin was maintained in the tissue culture medium at 5 μg/mL to maintain Cas9 expression. Cells were then transduced with constitutive sgRNA constructs cloned into the enh-sgRNA-mCherry-Puro backbone and were selected with puromycin at 2 μg/mL for 3–4 days. Cells were then seeded for viability assays or harvested for confirmation of pooled lysate gene KO by immunoblotting.

##### Inducible sgRNA system

For inducible-sgRNA-mediated gene KO, a two-part Cas9/inducible-sgRNA system was used. Cells were first transduced with Cas9 via lentiCas9-Blast (RRID: Addgene_52962) and were selected with blasticidin at 10 μg/mL for at least 10 days. Blasticidin was maintained in the tissue culture medium at 5 μg/mL to maintain Cas9 expression. Cells were then transduced with inducible sgRNA constructs cloned into the pRDA_355 (RRID: Addgene_187159) backbone and were selected with puromycin at 2 μg/mL for 3–4 days. Cells were then expanded under puromycin selection pressure (1 μg/mL) for use *in vivo*, or treated with Dox (at 500 ng/mL) at the time of seeding for viability assays. Maximal gene KO was confirmed by immunoblotting and TIDE assay as early as two days in Dox at 500 ng/mL.

##### Inducible shRNA system

The doxycycline-inducible Tet-pLKOpuro backbone was used for time-controlled gene KD as previously described.^15^ Briefly, ishRNA constructs were transduced into cells and selected with puromycin at 2 μg/mL for at least 3 days. Puromycin selection pressure was maintained at 1 μg/mL thereafter to maintain construct integration. Cells were then expanded for use *in vivo*, or treated with Dox at the time of seeding for viability assays. Dox concentration was optimized for maximal gene KD by day 2 via immunoblotting and found to be 100 ng/mL (for ishP3F1 studies) or 150 ng/mL (for ishFANCM studies).

#### Cell growth, drug-response, and apoptosis assays

##### Clonogenic growth assays

Clonogenic assays were mostly performed as previously described.^78^ Briefly, 1000 cells per well were seeded in triplicate in 12-well tissue culture plates either directly following puromycin selection of sgRNAs or after stable puromycin selection of inducible shRNA or sgRNA constructs. For *FANCM* rescue studies, ectopic *FANCM* construct was stably selected with G418 prior to sgRNA transduction and puromycin selection. For inducible construct studies, Dox was added at the indicated concentration at the time of clonogenic assay seeding and Dox-containing medium was refreshed every 48 h. The cells were grown in standard growth medium until the sgLuc, sgLacZ, isgNTC, or ishFANCM-SMC control populations reached confluency. The cells were then fixed and stained with 0.5% (w/v) crystal violet in 6.0% (v/v) glutaraldehyde (Thermo Fisher Scientific).

##### Cell viability assays

Cells were seeded in triplicate (or sextuplet) at 100 cells/well in 96-well tissue culture plates either directly following puromycin selection of sgRNAs or after stable puromycin selection of inducible shRNA or sgRNA constructs. At seeding, seven 96-well replicate plates were seeded for sequential measurements over the experimental time course. For *FANCM* rescue studies, ectopic *FANCM* construct was stably selected with G418 prior to sgRNA transduction and puromycin selection. For inducible construct studies, Dox was added at the indicated concentration at the time of assay seeding and Dox-containing medium was refreshed every 48 h. The cells were grown in standard growth medium until the sgLuc, sgLacZ, isgNTC, or ishFANCM-SMC control populations reached confluency. CellTiter-Glo (CTG, Promega) was used to quantify the relative number of live cells in each well on day 0 (2–3 h after seeding), and approximately every 48–72 h thereafter. To each well containing 100 μL of medium, 15 μL of CTG was added and allowed to incubate at room temperature for at least 20 min. Luminescence was then measured on a Tecan plate reader. Relative viability was determined by first normalizing the raw luminescence values for each condition to medium-only wells from that same day and plate, and then to day 0 luminescence values. Further normalization was done to control sgRNA or shRNA constructs as indicated.

##### Drug dose-response assays

For PIP-199 and ATR inhibitor studies, 100 cells/well were seeded in 96-well plates and treated with a dilution series of PIP-199, AZD6738 (ceralasertib), BAY1895344 (elimusertib), or DMSO vehicle. For shFANCM sensitization assays, doxycycline was added at the time of seeding to induce shFANCM or SMC. Viability was measured by CellTiter-Glo at the indicated time points as above, normalized to vehicle and/or control hairpin conditions, and GI_50_ values were estimated from nonlinear dose-response curves in Prism (RRID: SCR_002798).

##### Apoptosis assay

Rh4 Native cells expressing inducible shFANCM or SMC were treated with doxycycline for 0, 2, 4, or 6 days, harvested, and stained with Annexin V-APC and propidium iodide using the eBioscience Annexin V Apoptosis Detection Kit (Invitrogen) before flow cytometry. Cells were classified as viable (Annexin V−/PI−), early apoptotic (Annexin V+/PI−), late apoptotic/dead (Annexin V+/PI+), or dead/debris (Annexin V−/PI+).

#### Immunoblotting and densitometry

##### Western blotting (WB)

Immunoblotting was performed as previously described, with slight modification.^15,87^ Cells were suspended, washed in ice-cold PBS (Gibco), and stored as cell pellets at −80°C. Cell pellets were thawed on ice and protein lysates were prepared with 1× RIPA Lysis Buffer (Sigma) with 1× cOmplete protease inhibitor cocktail (Roche) and 1× PhosSTOP phosphatase inhibitor (Roche), rotated for 30 min at 4°C, passed through a QIAshredder column (Qiagen), cleared by centrifugation at 20,000 × g for 10 min at 4°C, and normalized by total protein content using Bradford analysis or BCA analysis. Protein lysate was run through 4–20% Mini-Protean TGX precast protein gels (Bio-Rad) and transferred to PVDF membrane using the Trans-Blot Turbo System (Bio-Rad). All membranes were blocked with 5% milk in PBS-T buffer. Membranes were probed with the following primary antibodies (diluted in 5% milk in PBS-T) overnight for at least 12 h at 4°C: FLAG (M2, Sigma #F3165, diluted 1:1000), PAX3 (GT1210, Thermo Fisher Scientific #MA5–31583, diluted 1:1000), FOXO1 (C29H4, CST #2880, 1:1000), Beta-Actin (13E5, CST #4970, diluted 1:4000), CKM (1E3, Abnova #H00001158-M02, 1:1000), FANCM (CV5.1, Novus #NBP2–50418, 1:1000), Vinculin (E1E9V, CST #13901, 1:1000), MYOD1 (D8G3, CST #13812, 1:1000), MYOG (5FD, Santa Cruz #sc-52903, 1:1000), MYL1 (EPR9934(B), Abcam #ab151749, 1:1000), MYH4 (MF20, DSHB, 1:1000), pRPA32-S8 (CST #83745, 1:1000), RPA32 (CST #52448, 1:1000), pH2A.X-S139 (20E3, CST #9718, 1:1000), H2A.X (D17A3, CST #7631, 1:1000), pATR-T1989 (GeneTex #GTX128145, 1:500), ATR (E1S3S, CST #13934, 1:1000), pATRIP-S224 (CST #5161, 1:1000), ATRIP (E2I9A, CST #97687, 1:1000), pCHK1-S317 (CST #2344, 1:1000), pCHK1-S345 (133D3, CST #2348, 1:1000), CHK1 (2G1D5, CST #2360, 1:1000), pATM-S1981 (D25E5, CST #13050, 1:1000), ATM (D2E2, CST #2873, 1:1000), pCHK2-T68 (C13C1, CST #2197, 1:1000), CHK2 (D9C6, CST #6334, 1:1000). Blots were then incubated with HRP-conjugated secondary antibody (CST) diluted 1:4000 in 5% milk in PBS-T for one hour at room temperature. Blots were developed with SuperSignal West Pico PLUS Chemiluminescent Substrate (Thermo Fisher Scientific) or ECL Western Blotting Substrate (Thermo Fisher Scientific) or WesternBright ECL HRP Substrate (Advansta) and detected using LabForce HyBlot CL Autoradiography Film (Thomas Scientific).

##### Densitometry

Western blot signal intensity was quantified in ImageJ.^88^ Signals for FANCM, PAX3::FOXO1, myogenic markers and DDR markers were obtained in triplicate to quadruplicate, normalized to the indicated loading or total-protein control, and then to the day-matched shFANCM-SMC condition unless otherwise indicated.

#### Microscopy, immunofluorescence, and image quantification

##### Brightfield

Cells were seeded in triplicate in 6-well plates (spheroid images) or 12-well plates (adherent cell images) and imaged in-plate as live cells on the indicated days (adherent cells) or when spheroids reached ∼100–200 μm in size. Cells were imaged using an AMG Evos Fl Fluorescence Microscope, followed by image processing in ImageJ software.^88^ A minimum of two images were taken per triplicate, and one representative image was chosen for presentation.

##### CKM immunofluorescence

To evaluate CKM signal upon *FANCM* loss, Rh4 Native cells were made to stably express ishRNA constructs as described above, and IF staining and imaging was performed as previously described.^89^ Cells were seeded directly onto μ-Slide 8 Well Glass Bottom Coverslips (Ibidi) and 150 ng/mL of Dox was added to the culture medium at the time of seeding. After three days, cells were fixed in 4% (w/v) ice-cold PFA and blocked in BSA in PBS prior to staining with anti-CKM antibody (1E3, Abnova #H00001158-M02), followed by anti-mouse-Alexa-647 (Abnova) antibody. To visualize nuclei, fixed cells were stained with Hoechst 33342 (Thermo Fisher Scientific) and outer cell boundaries (cytoskeletons) were visualized by phalloidin staining using an Alexa Fluor 488 Phalloidin probe (Thermo Fisher Scientific). A slide containing unstained cells was used as a negative control. Cells were imaged using an AMG Evos Fl Fluorescence Microscope, followed by image processing in ImageJ software.^88^ A minimum of two images from non-overlapping fields of view were taken per replicate, and one representative image was chosen for presentation. For quantification, mean cytoplasmic CKM intensity was quantified in ImageJ software from triplicate samples. Cytoplasm was defined by phalloidin-positive cell masks with nuclear exclusion.

##### γH2A.X staining, imaging, and foci quantification

Rh4 Native cells transduced with doxycycline-inducible shRNAs targeting FANCM or SMC were induced with 150 ng/mL doxycycline for 72 h. Induced and non-induced control cells were detached with 0.05% trypsin-EDTA and seeded at single-cell density on #1.5 cover glass. Cells were treated with vehicle, 0.1 μM methotrexate, or 10 μM etoposide while doxycycline induction continued for 16 h. Cells were fixed in 4% paraformaldehyde in PBS, quenched with 1 M glycine in PBS, and permeabilized with 0.3% Triton X-100 in PBS for 10 min each at room temperature. After PBS washes, cells were blocked for 30 min in 5% bovine serum albumin (BSA) in PBS and stained overnight at 4°C with phospho-Histone H2A.X (Ser139) (20E3) rabbit monoclonal antibody (Cell Signaling Technology #9718; 1:100) diluted in 5% BSA/PBS. Cells were stained for one hour at room temperature with goat anti-rabbit Alexa Fluor 488 (Thermo Fisher Scientific #A-11008; 1:1,000) and DAPI (Thermo Fisher Scientific #62248), mounted with ProLong Gold (Thermo Fisher Scientific #P36934), and imaged on an EVOS M5000 epifluorescence microscope (Thermo Fisher Scientific) with GFP and DAPI filter sets and an Invitrogen Plan FL PH 40×/0.65 RMS objective. Acquisition settings were held constant within each experiment. Images were analyzed in CellProfiler v4.2.8.^90^ Nuclei were segmented from DAPI using a two-class Otsu threshold. γH2A.X puncta in the 488-nm channel were enhanced and segmented using a minimum cross-entropy threshold. Puncta overlapping nuclear masks were counted per nucleus, and integrated puncta fluorescence intensity was measured from the original 488-nm channel. Nuclei with one or more segmented puncta were classified as γH2A.X-positive.

##### Proximity ligation assays

Rh4 Native cells carrying the indicated inducible shRNA and RNase H1 constructs were seeded on cover glass and treated with doxycycline as indicated. Cells were pre-extracted with ice-cold 0.5% PBS-Triton X-100 on ice for 30 s, fixed with 4% PFA for 15 min at room temperature, and permeabilized with 0.2% PBS-Triton X-100 for 10 min at room temperature. Cells were incubated overnight at 4°C with primary antibody pairs: PCNA (Santa Cruz Biotechnology, sc-56; 1:500) with RNAPII phosphorylated at Ser2 (Novus Biologicals, NB100–1805; 1:500) for TRC PLA, or phospho-RPA32 Ser33 (Novus Biologicals, NB100–544; 1:500) with S9.6 (home-made from Sigma; 1:1,000) for R-loop-associated PLA. PLA was performed using Duolink *In Situ* PLA reagents (Sigma; DUO92001, DUO92002, DUO92008) according to the manufacturer’s protocol, counterstained with DAPI, and imaged by fluorescence microscopy. PLA foci were quantified per nucleus using ImageJ software.^88^ Wild-type RNase H1 rescue was used to assess RNA-DNA hybrid-sensitive PLA signal.

#### RT-qPCR and *PAX3::FOXO1* transcript-stability assays

##### RT-qPCR analysis

Total RNA was isolated using the indicated RNA purification kit and analyzed by one-step RT-qPCR using GoTaq 1-Step RT-qPCR System (Promega) or the previously described two-step RT-qPCR workflow, as indicated.^91^ Expression was normalized to the indicated housekeeping transcript using ΔCq values, and relative expression was calculated as 2^−ΔΔCq^. Primers used in expression analyses were: Beta-actin (F: AGAGCTACGAGCTGCCTGAC, R: AGCACTGTGTTGGCGTACAG), FANCM (F: AGGGATGGAATG AGGCAAAGT, R: AGTGGTTGATTCTTGAGCTGGT), PAX3::FOXO1 (F: CATGGATTTTCCAGCTATACAGAC, R: GTTCCTTCATTCTG CACACG), GAPDH (F: GGAGCGAGATCCCTCCAAAAT, R: GGCTGTTGTCATACTTCTCATGG).

##### PAX3:FOXO1 transcript stability assay

Rh4 cells transduced with inducible shFANCM or SMC were induced with 150 ng/mL doxycycline for 96 h. Transcription was inhibited with 50 μM actinomycin D (Thermo Fisher Scientific/Gibco) and RNA was harvested at 0, 30, 60, 120, and 180 min using the Quick-RNA Miniprep Plus Kit (Zymo Research, R1058). RT-qPCR was performed with GoTaq 1-Step RT-qPCR System (Promega, A6020) according to the manufacturer’s protocol with 25 ng input RNA per reaction. PAX3:FOXO1 and GAPDH Cq values were exported from a CFX Opus384 (Bio-Rad). ΔCq values were calculated by subtracting the matched GAPDH Cq value from the PAX3:FOXO1 Cq value. Transcript levels were normalized to the matched 0-min condition and compared between shFANCM-SMC and shFANCM conditions. The same PAX3:FOXO1 and GAPDH primer pairs listed in the RT-qPCR analysis section were used for this assay.

#### RNA-seq sample preparation and analysis

##### PAX3:FOXO1 KD

Rh4 Clone cells were made to stably express ishNTC or ishP3F1 as previously described. Cells were prepared in triplicate for each condition, treated with Dox at 100 ng/mL for 7 days, and then were pelleted and frozen at −80°C. Pellets were thawed on ice, and RNA was homogenized and purified using the QIAshredder and RNeasy Kits (Qiagen). Purified RNA was submitted for poly(A) enrichment and library preparation using the KAPA mRNA HyperPrep Kit (Roche), and sequenced at the Duke University Sequencing and Genomic Technologies Core Facility on a NovaSeq 6000 (Illumina) S-Prime flow cell with 50 bp paired-end sequencing.

##### FANCM KD

Rh4 Clone cells were made to stably express ishFANCM-SMC or ishFANCM as previously described. Cells were prepared in triplicate for each condition, treated with Dox at 150 ng/mL for 2 days, and then were pelleted and frozen at −80°C. Pellets were thawed on ice, and RNA was homogenized and purified using the QIAshredder and RNeasy Kits (Qiagen). Purified RNA was submitted for poly(A) enrichment and library preparation using the KAPA mRNA HyperPrep Kit (Roche), and sequenced at the Duke University Sequencing and Genomic Technologies Core Facility on one lane of a NovaSeq X Plus (Illumina) 10B flow cell with 50 bp paired-end sequencing.

##### Irradiation control

For the DNA-damage comparator in the PAX3:FOXO1 target gene GSEA analysis, Rh4 Clone cells were exposed to 5 Gy irradiation in triplicate and then grown under standard tissue culture conditions for 24 h. After 24 h, treated and parental control replicate samples were washed and suspended in RNA/DNA Shield reagent (Zymo Research). Lysate was frozen at −80°C and sent to Plasmidsaurus for RNA-seq. Raw reads were downloaded and analyzed using the same GSEA workflow as in the shFANCM KD condition.

##### Analysis

Raw FASTQ files were first validated and trimmed with TrimGalore (RRID: SCR_011847). Preprocessed FASTQ files were indexed against the GRCh38 human genome assembly using HISAT2 (RRID: SCR_015530).^72^ Output SAM files were converted to BAM files, indexed, and sorted using SAMtools (RRID: SCR_002105).^74^ FeatureCounts (RRID: SCR_012919) was used to get raw gene read counts, which were then normalized and processed for differential gene expression using the DESeq2 (RRID: SCR_015687) pipeline in R version 4.4.2.^70,92^ Log-fold change values from DESeq2 were analyzed with gene set enrichment analysis (GSEA) via ClusterProfiler (RRID: SCR_016884) or FGSEA (RRID: SCR_020938) in R version 4.4.2.^65,71,92^

#### ChIP-seq sample preparation and analysis

##### ChIP protocol

Parental Rh4 Native cells (for H3K27ac ChIP) or Rh4 Native derivative cell lines stably expressing ishFANCM-SMC or ishFANCM (for pRPA32 ChIP) were prepared in triplicate, grown to confluency (Rh4 Native cells) or treated with 150 ng/mL doxycycline for two days (ishRNA derivatives), trypsinized, washed in PBS (Gibco), and crosslinked in 1% w/v formaldehyde (from 16% w/v methanol-free Formaldehyde, Thermo Fisher Scientific) by incubating at room temperature for 15 min on a rocking platform. Crosslinking was quenched with 2.5 M glycine (Sigma) by rocking at room temperature for 5 min. Cells were then washed twice in ice-cold PBS containing 2× Protease Inhibitor (PI: cOmplete Protease Inhibitor, Roche), pelleted, and stored at −80°C.

Frozen crosslinked pellets were thawed on ice and lysed by suspension in Lysis Buffer 1: 50 mM HEPES (Gibco), 140 mM NaCl (Invitrogen), 1 mM EDTA (Invitrogen), 10% Glycerol (Thermo Fisher Scientific), 0.50% NP-40 (Thermo Fisher Scientific), 0.25% Triton X-100 (Thermo Fisher Scientific), and 2× PI. Nuclei were pelleted by centrifugation at 1,350 × g for 10 min at 4°C and washed in Lysis Buffer 2: 10 mM Tris-HCl pH 7.0 (Thermo Fisher Scientific), 200 mM NaCl, 1 mM EDTA, 0.5 mM EGTA (Sigma), and 2× PI. Nuclei were pelleted by centrifugation at 1,350 × g for 10 min at 4°C. Nuclei were resuspended, lysed, and sonicated in Sonication Buffer: 50 mM Tris-HCl pH 7.0, 140 mM NaCl, 1 mM EDTA, 1 mM EGTA, 1% Triton X-100, 0.1% Na-Deoxycholate (DOC, Sigma). Sonication was performed on an ME220 Covaris machine controlled with SonoLab 8.0.0 at 7°C with 2 million cells/130 μL microtube (microTUBE-130 AFA Fiber Screw-Cap, Covaris) for 5 min at 70 W peak power with duty factor 20% and 1,000 cycles/burst. Shearing was confirmed on an Agilent 2200 TapeStation using a D1000 High Sensitivity ScreenTape (Agilent).

Sheared chromatin was diluted in Dilution Buffer (50 mM Tris-HCl pH 7.0, 140 mM NaCl, 1 mM EDTA, 1 mM EGTA, and 2× PI) and 50 μL per replicate was isolated for whole cell extract (WCE) samples. Chromatin from 2.5 million cells/replicate of Rh4 Native cells was mixed with 3 μL of anti-H3K27ac antibody (Active Motif, #39133) and rotated at 4°C overnight. Chromatin from 4.5 million cells/replicate of Rh4 Native + ishFANCM-SMC or ishFANCM was mixed with 5 μL of anti-pRPA32-S33 antibody (Bethyl, #A300–246A) and rotated at 4°C overnight. 50 μL per sample of Recombinant Protein G – Sepharose beads (Invitrogen) was washed in Dilution Buffer and added to each antibody/chromatin mixture and rotated end-over-end at 4°C for 2 h. Beads were then washed with ice-cold RIPA Wash Buffer (0.1% SDS (CST), 0.1% DOC, 1% Triton X-100, 1 mM EDTA, 10 mM Tris-HCl pH 8.0 (Thermo Fisher Scientific), and 150 mM NaCl) and pelleted by magnet. Beads were then washed with ice-cold RIPA 500 Wash Buffer (0.1% SDS, 0.1% DOC, 1% Triton X-100, 1 mM EDTA, 10 mM Tris-HCl pH 8.0, and 500 mM NaCl) and pelleted by magnet. Beads were then washed in ice-cold LiCl Wash Buffer (10 mM Tris-HCl pH 8.0, 250 mM LiCl (Sigma), 0.5% Triton X-100, and 0.5% DOC) and pelleted by magnet. Beads were then washed in ice-cold Final Wash Buffer (10 mM Tris-HCl pH 8.5) and pelleted by magnet. Protein-bound complex was eluted off beads using ChIP Elution Buffer (10 mM Tris-HCl pH 8.0, 5 mM EDTA, 300 mM NaCl, and 0.1% SDS). Buffer concentrations of WCE samples were matched to ChIP Elution Buffer, and all samples were reverse-crosslinked by incubating overnight at 65°C in the thermocycler in Reverse-Crosslink Buffer: 250 mM Tris-HCl pH 6.5 (Thermo Fisher Scientific), 1.25 M NaCl, 62.5 mM EDTA, 5 mg/mL Proteinase K (Thermo Fisher Scientific), and 62.5 mg/mL RNase A (Thermo Fisher Scientific). DNA was then purified with 2.2× SPRIselect Beads (Beckman) and eluted in 10 mM Tris-HCl pH 8.0.

##### Sequencing

For each replicate, DNA libraries were end prepared and adapter ligated using the NEBNext Ultra II DNA Library Prep Kit for Illumina (NEB), and indexed using the NEBNext Multiplex Oligos for Illumina (NEB) according to manufacturer recommendations. Amplified indexed libraries were quantified using the Quant-iT dsDNA High Sensitivity Assay Kit (Thermo Fisher Scientific) and mixed in equimolar ratios for pooled library sequencing. Pooled library was sequenced at the Duke University Sequencing and Genomic Technologies Core Facility on a NovaSeq X Plus (Illumina) 1.5B flow cell with 150 bp paired-end sequencing.

##### Analysis

Raw FASTQ sequencing files were first quality tested with FastQC (RRID: SCR_014583) and reads were then merged across flow cell lanes. Merged FASTQ files were aligned to the GRCh38 human genome assembly using BWA (RRID: SCR_010910).^64^ Output SAM files were converted to BAM files, indexed, and sorted using SAMtools (RRID: SCR_002105).^74^ Duplicate reads were marked and removed with PICARD (RRID: SCR_006525) and bigWig (bW) files were generated from the bamCoverage function in deepTools (RRID: SCR_016366) with the following settings: bin size of 20, RPKM normalization, smooth length of 60, extend reads to 150, and center reads.^68^ For visualized tracks, bW sample files were normalized by ratio to respective WCE bW files using the bigwigCompare function in deepTools, and representative replicate files were depicted using the Integrative Genomics Viewer (IGV; RRID: SCR_011793).^93^ For pRPA32 samples, MACS2 (RRID: SCR_013291) was used to call peaks relative to WCE samples and to generate narrowPeak files.^94^ To obtain the genomic loci enriched in pRPA32 for ishFANCM samples relative to ishFANCM-SMC controls, DiffBind (RRID: SCR_012918) was used. Genomic loci enriched in pRPA32 signal were mapped to genes and gene overrepresentation analysis (ORA) was performed with ClusterProfiler (RRID: SCR_016884).^65^

#### DepMap and STRING analyses

##### DepMap

The Two Class Comparison function in the DepMap Custom Analyses portal was used for the DepMap comparison.^32^ Fusion positive cell lines were sorted into the “In Group” based on *PAX3::FOXO1* expression, which included the Rh4, Rh28, RhJT, SCMCRM2, JR, and Rh30 cell lines. The “Out Group” comprised all other cell lines. The two groups were compared on the basis of DepMap Public 23Q4+Score, Chronos for the *FANCM* Gene Effect.^32,95^

##### STRING

To perform functional protein association network (STRING) analysis, indicated gene lists were uploaded and matched to human proteins using the multiple protein upload function in the STRING pipeline.^33^ When indicated, K-means clustering was applied to separate protein clusters into groups based on their interaction partners.

#### *In vivo* inducible FANCM xenograft studies and TIDE analysis

##### Inducible shFANCM knockdown studies

The Rh4 Clone cell line was transduced with Dox-inducible shRNA constructs, selected in puromycin, and expanded in tetracycline-free FBS as previously described. All cells were confirmed to be *Mycoplasma* negative before injection *in vivo*. Cells were resuspended in sterile PBS and mixed with Matrigel (Corning) at a concentration of 25 million cells/mL. Injections of 100 μL (or 2.5 million cells) were made into the flank of 10 6-week-old NSG mice per ishRNA cohort. Tumor size was measured every 3–4 days with calipers, and tumor volume was calculated by the formula: V = (L × W × W)/2 (L = longest diameter and W = shortest diameter). When tumors reached ∼100–200 mm^3^ in size, three tumors were harvested for pre-Dox (day 0) control samples. For the remaining 7 mice, Dox was administered by oral gavage for 10 days at 1 mg per mouse and the rodent diet was changed to Dox-containing chow, which remained available for the course of the study. Tumor size was monitored until IACUC-approved endpoints were met, including when tumors reached ∼750 mm^3^, tumors ulcerated, or the mice displayed clinical impairments.

##### Inducible sgFANCM

The Rh4 Clone cell line stably expressing Cas9 was transduced with Dox-inducible sgRNA constructs, selected in puromycin, and expanded in tetracycline-free FBS as previously described. All cells were confirmed to be *Mycoplasma* negative before injection *in vivo*. Cells were resuspended in sterile PBS and mixed with Matrigel (Corning) at a concentration of 25 million cells/mL. Injections of 100 μL (2.5 million cells) were made into the flank of 13 6-week-old NSG mice per isgRNA construct. Tumor size was measured every 3–4 days with calipers, and tumor volume was calculated by the formula: V = (L × W × W)/2 (L = longest diameter and W = shortest diameter). When tumors reached ∼100–200 mm^3^ in size, three tumors were harvested for pre-Dox (day 0) control samples. For three of the remaining 10 mice, Dox was administered by oral gavage for 5 days at 1 mg per mouse, at which point tumors were harvested for early Dox induction (day 5) samples. For the remaining 7 mice, Dox was administered by oral gavage for 10 days at 1 mg per mouse and the rodent diet was changed to Dox-containing chow, which remained available for the course of the study. Tumor size was monitored until IACUC-approved endpoints, including when tumors reached ∼1,500 mm^3^, tumors ulcerated, or the mice displayed clinical impairments.

##### TIDE assay

Genomic DNA was purified from harvested tumors from isgRNA-containing xenografts using the Quick DNA Kit (Zymo Research). Genomic DNA was used as PCR template using NEBNext Ultra II Q5 Master Mix (NEB) to amplify the *FANCM* locus surrounding the PAM sites targeted by the isgFANCM-1 sequence (F: GCTCAAGTATCTCCCGATCATCTG, R: CTGGCACTTATCTAGGA CTACAACC) and the isgFANCM-2 sequence (F: CCTGGGCTCAAGCGATTTCCCC, R: TCATGGTTTCTTCTGACCCCACT). Sanger sequencing of the isgFANCM-1-targeted amplicons was done using the forward amplicon primer, and sequencing of the isgFANCM-2-targeted amplicons was done with a sequencing primer nested within the amplicon sequence (GTGTTGACATTACAGGCATGAGCTG). Indels were deconvoluted using the DECODR analysis pipeline.^67^

#### FANCM hotspot validation, amplicon sequencing, and structural modeling

##### Individual sgRNAs

Rh4 Clone and RD cells expressing doxycycline-inducible sgRNAs targeting selected regions of *FANCM* were harvested 1 and 5 days after induction with 500 ng/mL doxycycline. The sgRNAs targeted regions encoding either the N-terminal translocase/helicase domain or the C-terminal FAAP24-interacting region: isgFANCM-271, CCCTACCAATTGCCCAGTGC, and isgFANCM-5774, AGCCTGCTGACTACCTTAAT. Genomic DNA was isolated from triplicate samples for each sgRNA and time point, and target loci were PCR-amplified for amplicon sequencing using the following primers: isgFANCM-271 forward, ACGCTTTTTCAGACGT GGGGCT, and reverse, ACTGTCCCAACCATGACTAGGCT; isgFANCM-5774 forward, AGGGTAGGGAAGTAATTGGCAAGA, and reverse, AGAACACCAAACACAGTAGCCT. For annotation, the predicted Cas9 cut sites were assigned to approximate FANCM amino acid positions 90 for isgFANCM-271 and 1925 for isgFANCM-5774.

##### Allele quantification and residue-level depletion hotspot scoring

Extracted genomic DNA was sent to Plasmidsaurus via their amplicon sequencing pathway. Raw FASTQ files were downloaded from Plasmidsaurus, assigned to the expected amplicon, and individual sequencing reads were processed to identify unique edited amino acid haplotypes. For each sample, allele frequency was calculated as reads supporting the allele divided by total QC-passing reads. For each allele, Δ(D5-D1) = frequency D5 minus frequency D1. Alleles were classified as depleted, increased, or stable/not significant using the direction of Δ(D5-D1) and the D1-versus-D5 classification *p* values. Allele frequencies were summarized across triplicate samples at day 1 and day 5. The full allele-level table used for trajectory plots and modeled-allele selection is provided as Table S9. Each edited allele was then expanded into the amino acid positions altered by deletion or substitution. Significant depleted alleles contributed positive evidence to altered residues, significant increased alleles contributed negative evidence, and stable or non-significant alleles contributed zero. To prevent long edits from dominating the analysis, each allele’s contribution was divided by the number of amino acids altered by that allele. Thus, the residue score represents the sum of length-normalized depletion and enrichment evidence assigned to each residue. Positive scores indicate depletion-biased evidence, whereas negative scores indicate enrichment-biased or counter-depletion evidence. Additional residue-level metrics included deletion-only scores, altered-residue counts, depleted and increased allele counts, and guide-preserving cut-site permutation controls. Scores were used to visualize local evidence near selected alleles and not to define uniformly essential domains. Allele-level depletion and enrichment values are provided in Table S9.

##### Divergent-phyla homology analysis

*FANCM* and *FANCM*-like orthologs from vertebrate, fish, invertebrate, plant, and fungal species were aligned over local windows surrounding the selected alleles. Each aligned residue was classified relative to human *FANCM* as exact, conservative, mismatch, gap, or no sequence, and selected allele windows were highlighted. Conservation heatmaps were made to display the aligned residue identity at each position and highlight the selected allele window.

##### Selection of modeled alleles

The N-terminal allele spanning FANCM residues 91–94 with residues 91, 92, and 94 deleted, termed Δ91–94 for simplicity, was selected because it was among the strongest depleted alleles in the N-terminal translocase region. The C-terminal allele with cleanly deleted residues 1922–1925 was selected because it was the most depleted allele mapped to the FAAP24-interacting C-terminal region. Exact sequences of the two modeled mutant FANCM protein segments are provided in Table S10.

##### ColabFold2 modeling and structural rendering

Human FANCM mutant sequences were generated by applying the selected in-frame deletions to local FANCM sequence inputs and submitted to ColabFold2 for hypothesis-generating structure prediction.^66^ The Δ91–94 N-terminal model was compared with the FANCM N-terminal translocase/DNA reference structure 9EL5.^96^ The Δ1922–1925 C-terminal model was compared with the DNA-bound FANCM-FAAP24 reference structure 9HJO.^96^ Predicted mutant structures were aligned to matched FANCM Cα atoms in the corresponding reference structures, and per-residue displacement was calculated as the Euclidean distance between matched Cα coordinates after alignment. Mutant models were colored by displacement in 0.5 Å bins and rendered in open-source PyMOL (version 3.0.5) with relevant DNA and protein partners retained for context.^97^ The models were treated as hypothesis-generating structural predictions rather than experimentally determined mutant structures.

### QUANTIFICATION AND STATISTICAL ANALYSIS

Unless otherwise noted, data are shown as mean ± SEM from independent biological replicates or technical replicates as indicated. Pairwise comparisons used two-sided t tests; multiple comparisons used ANOVA with appropriate post hoc correction as specified in figure legends. Growth curves were analyzed by two-way ANOVA or curve-fit/regression models as indicated; xenograft tumor-growth curves were compared by ordinary least-squares regression of fitted polynomial models; survival was compared by log rank test. Differential sequencing analyses used MAGeCK, DESeq2, DiffBind, or the indicated allele-classification pipeline. Analyses and plotting were performed in Prism 11, R, JMP Pro 17, and the indicated analysis pipelines. Significance notation: *p < 0.05, **p < 0.01, ***p < 0.001, ****p < 0.0001, NS, not significant.

## Supplementary Material

1

2

3

5

6

7

8

9

10

11

12

SUPPLEMENTAL INFORMATION

Supplemental information can be found online at https://doi.org/10.1016/j.celrep.2026.117960.

## Figures and Tables

**Figure 1.**
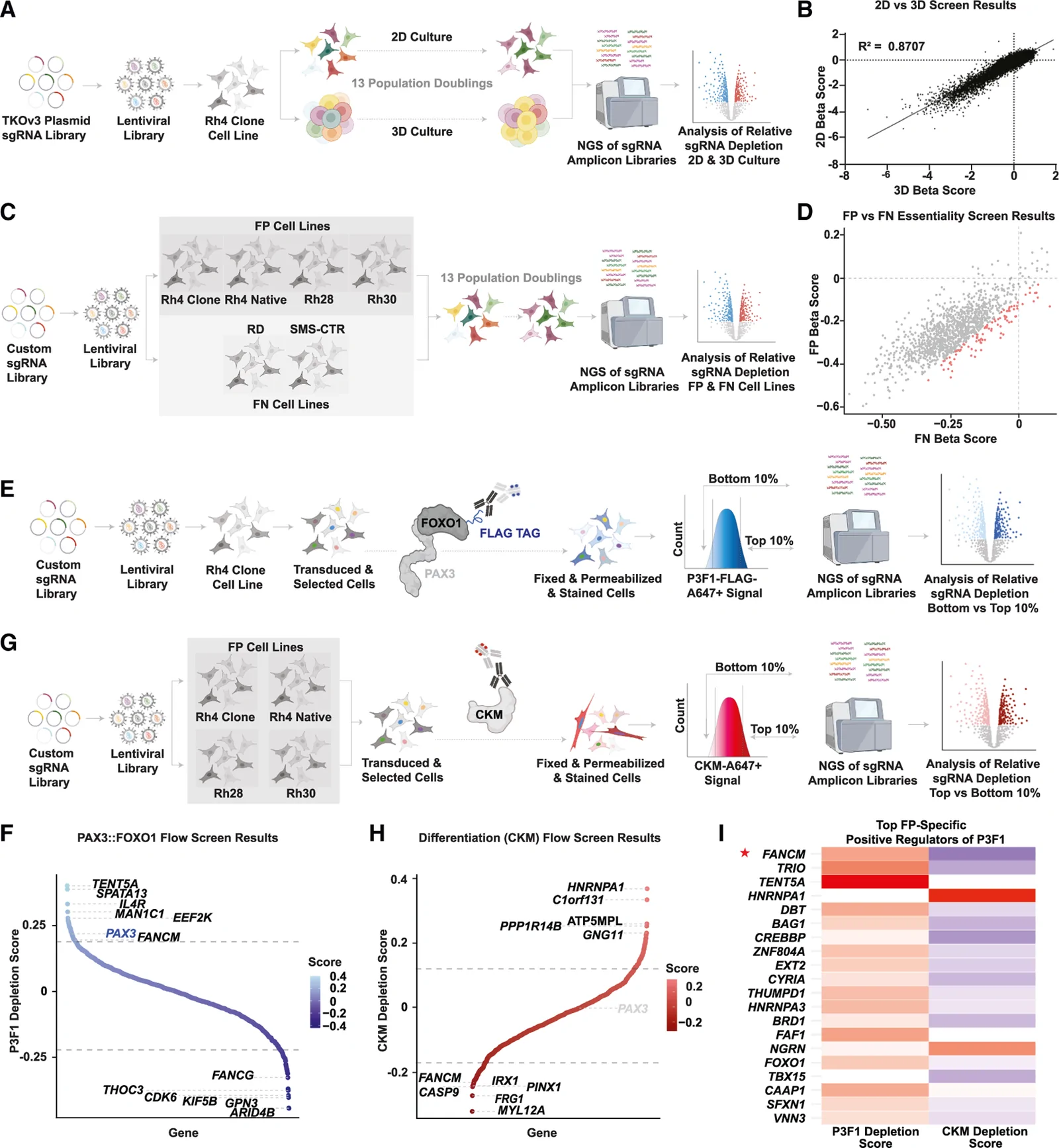
Multimodal CRISPR-Cas9 screens nominate *FANCM* as a regulator of *PAX3::FOXO1* abundance and myogenic differentiation in FP RMS (A) Schematic of the TKOv3 genome-scale CRISPR-Cas9 knockout screen performed in Rh4 clone cells in 2D monolayer and 3D spheroid culture. (B) Dot plot comparing 2D and 3D MAGeCK MLE beta scores from the genome-scale screens. Linear regression R^2^ value is shown. (C) Schematic of the custom sgRNA sub-library essentiality screen performed across FP and FN RMS cell lines. (D) FP versus FN beta scores from the custom sub-library essentiality screen. Genes preferentially required in FP RMS cells are highlighted. (E) Schematic of the PAX3::FOXO1-FLAG flow-based CRISPR-Cas9 screen in Rh4 clone cells. (F) Ranked PAX3::FOXO1-FLAG depletion scores from the flow-based screen. Selected genes are labeled. (G) Schematic of the CKM flow-based CRISPR-Cas9 differentiation screen performed in FP RMS cell lines. (H) Ranked CKM depletion scores from the CKM flow-based screen. Selected genes are labeled. (I) Heatmap of top FP-specific positive regulators of *PAX3::FOXO1*, ranked by combined P3F1 and CKM depletion scores. *FANCM* is highlighted.

**Figure 2.**
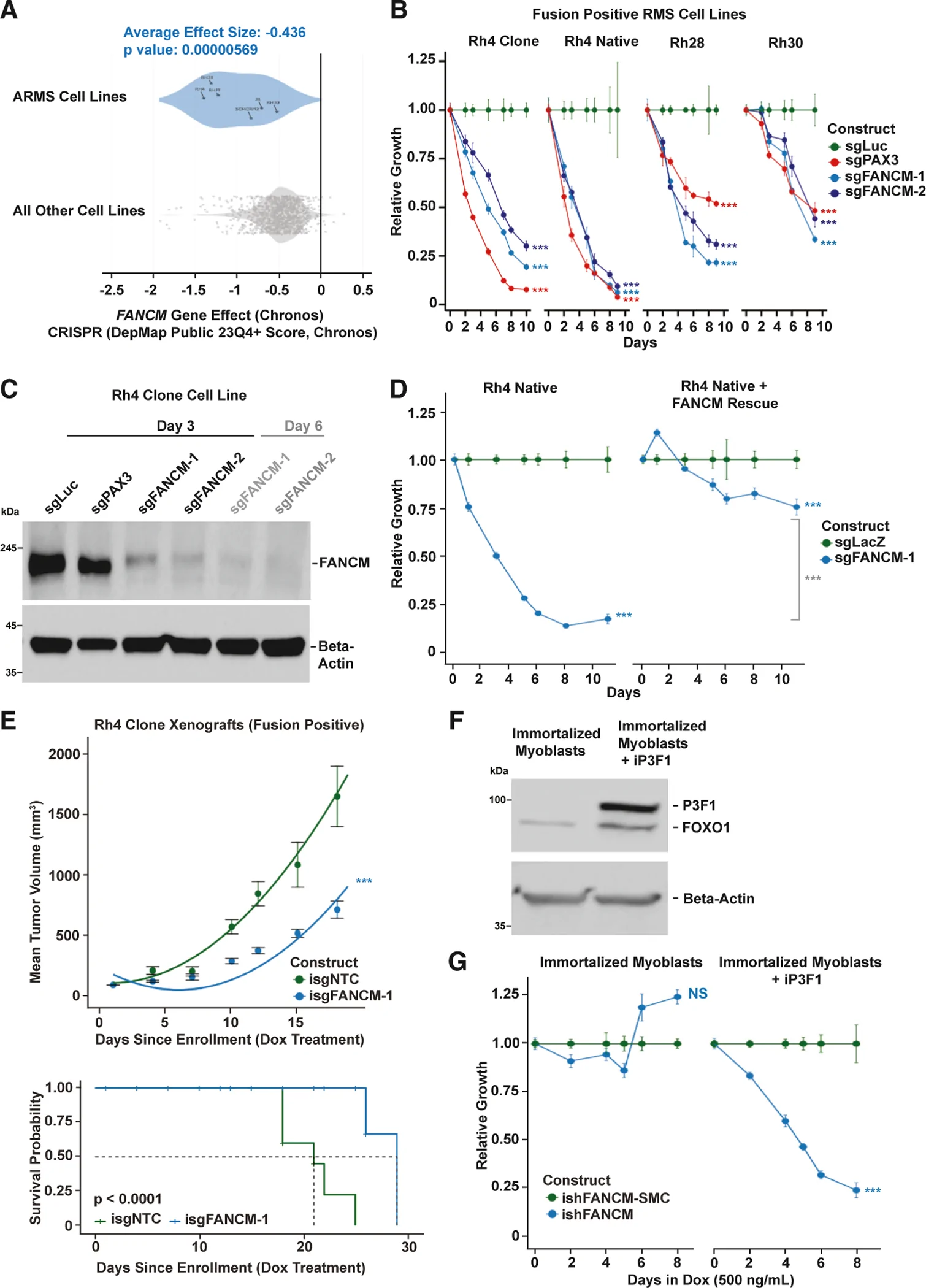
*FANCM* is a dependency in FP RMS models *in vitro* and *in vivo* (A) DepMap *FANCM* gene effect scores comparing FP ARMS cell lines with all other cancer cell lines. (B) Growth curves after sgRNA-mediated *FANCM* knockout in four FP RMS cell lines. sgLuc is the negative control and sgPAX3 is the positive-control dependency. Data are represented as mean ± SEM. *** indicates *p* < 0.001 by two-way ANOVA with Dunnett’s multiple-comparisons test. (C) Western blot validation of *FANCM* knockout in Rh4 clone cells after sgRNA transduction and puromycin selection. (D) Growth curves showing rescue of the *FANCM* knockout growth defect by ectopic sgRNA-resistant *FANCM* in Rh4 native cells. Data are represented as mean ± SEM. *** indicates *p* < 0.001 by two-way ANOVA with Dunnett’s multiple-comparisons test. (E) Rh4 Clone xenograft tumor growth curves and Kaplan-Meier survival curves after induction of control or *FANCM*-targeting sgRNAs. Tumor volumes are represented as mean ± SEM. *** indicates *p* < 0.001 by ordinary least-squares polynomial regression. Survival was compared by log rank test. (F) Western blot validation of inducible *PAX3::FOXO1* expression in immortalized human myoblasts. (G) Growth curves of immortalized myoblasts with or without inducible *PAX3::FOXO1* expression after simultaneous induction of shFANCM-SMC or shFANCM. Data are represented as mean ± SEM. *** indicates *p* < 0.001 and NS indicates not significant by two-way ANOVA with Dunnett’s multiple-comparisons test.

**Figure 3.**
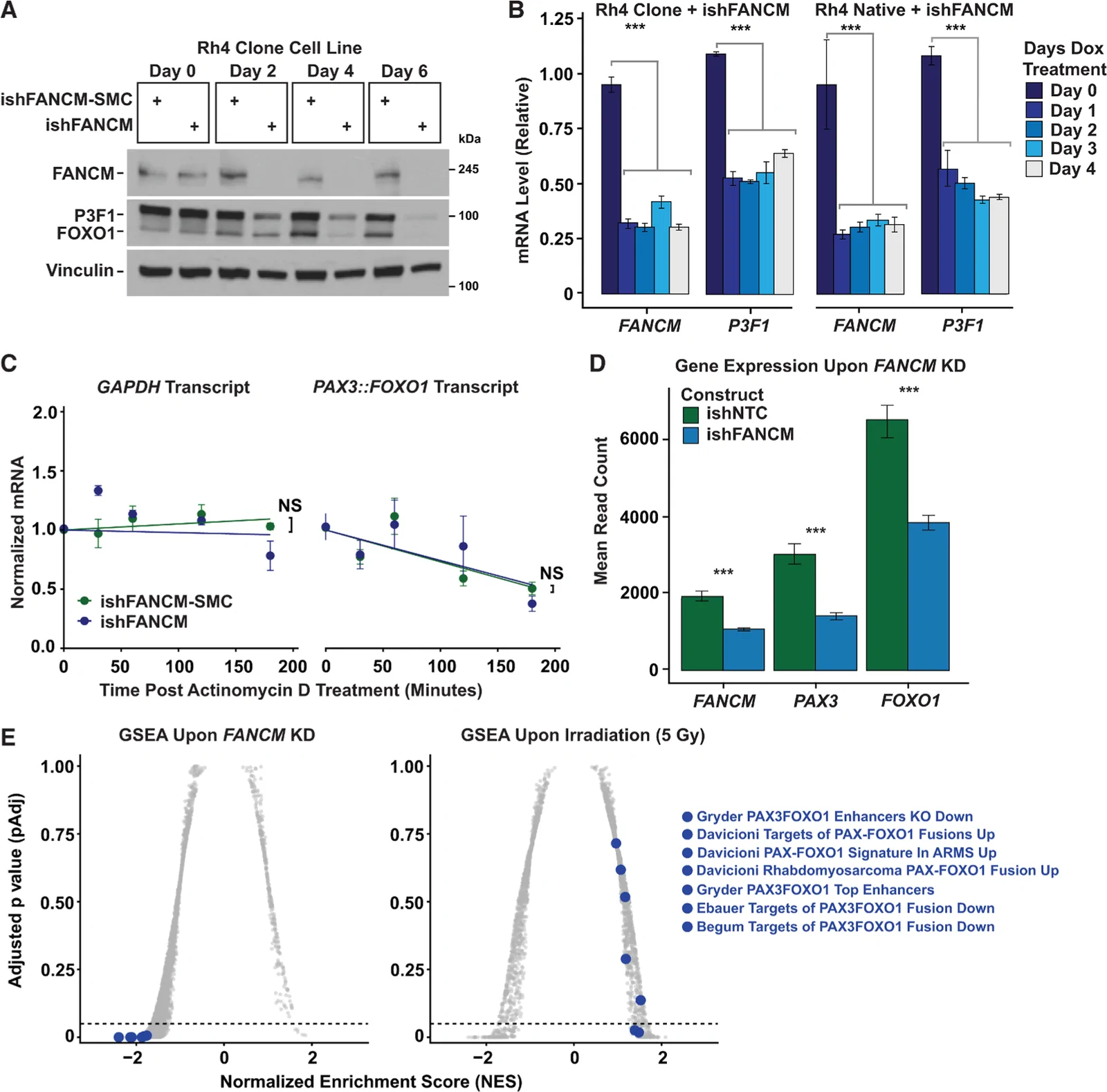
*FANCM* loss reduces PAX3::FOXO1 levels and suppresses the fusion transcriptional program (A) Western blot time course showing FANCM and PAX3::FOXO1 levels after inducible shFANCM or seed-matched control in Rh4 clone cells. (B) RT-qPCR time course of *FANCM* and *PAX3::FOXO1* transcript levels after shFANCM induction in Rh4 clone and Rh4 native cells, normalized to housekeeping transcript and day 0. Data are represented as mean ± SEM. *** indicates *p* < 0.001 by one-way ANOVA with Dunnett’s multiple-comparisons test. (C) Actinomycin D chase assay measuring *GAPDH* and *PAX3::FOXO1* transcript degradation after *FANCM* KD compared with seed-matched control. Data are represented as mean ± SEM. NS indicates not significant by an F test comparing linear-regression slopes. (D) RNA-seq mean read counts for *FANCM*, *PAX3*, and *FOXO1* after *FANCM* KD compared with non-targeting control. Data are represented as mean ± SEM. *** indicates *p* < 0.001, unpaired two-sided *t* test. (E) GSEA summary of published PAX3::FOXO1 target gene sets after *FANCM* KD or 5 Gy irradiation. The dashed line indicates adjusted *p* value = 0.05.

**Figure 4.**
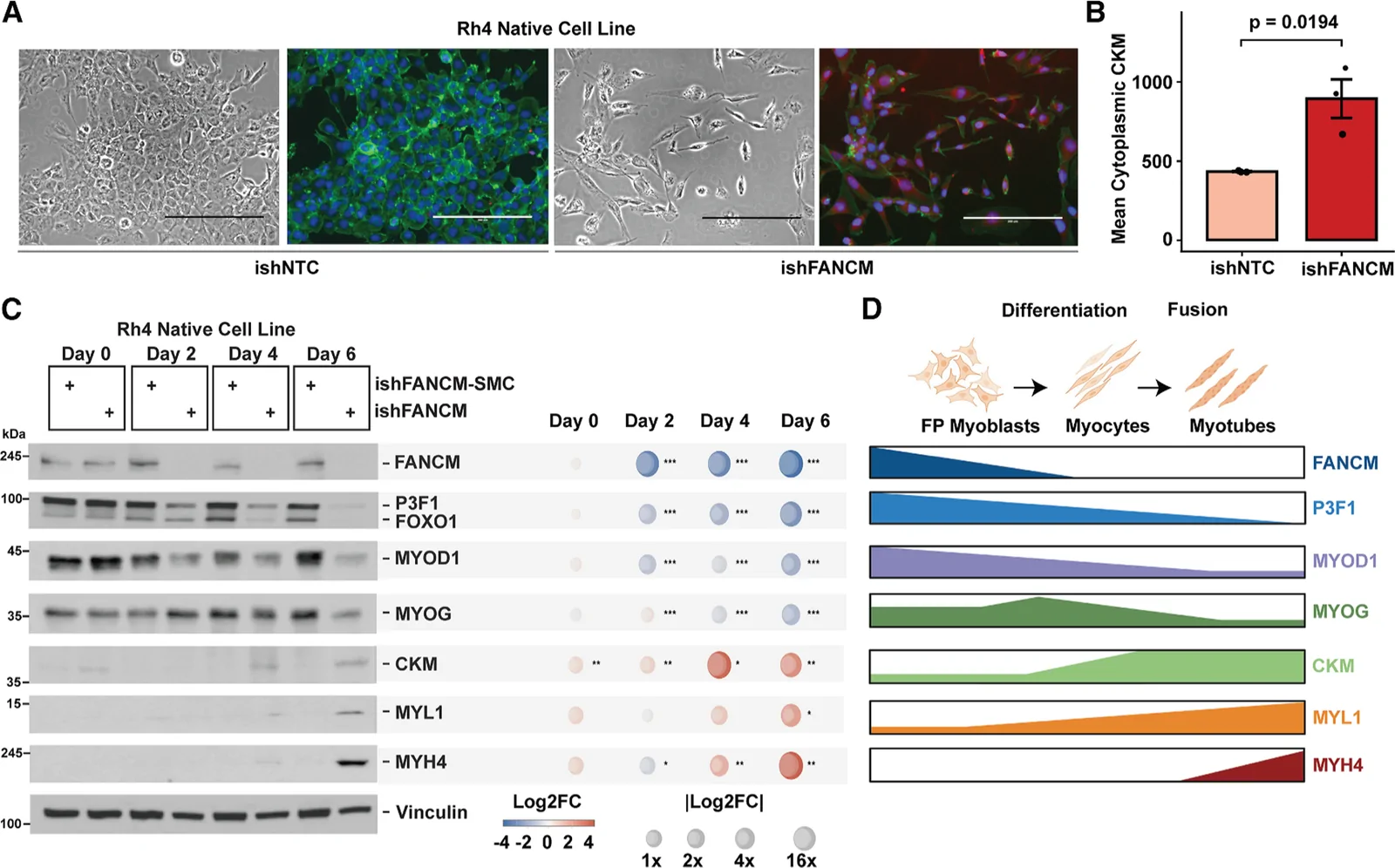
*FANCM* loss induces myogenic differentiation in FP RMS cells (A) Representative brightfield and immunofluorescence images of Rh4 native cells after inducible shNTC or shFANCM. Nuclei, F-actin, and CKM are shown. Scale bars, 200 μm. (B) Quantification of mean cytoplasmic CKM intensity from immunofluorescence images. Data are represented as mean ± SEM. *p* = 0.0194 by unpaired two-sided *t* test. (C) Western blot time course and densitometric summary of FANCM, PAX3::FOXO1, MYOD1, MYOG, CKM, MYL1, and MYH4 after inducible shFANCM compared with seed-matched control. Signal intensities were normalized to loading control and day-matched seed-matched control. Bubble size and color represent mean log2 fold change and absolute log2 fold change, respectively. *, **, and *** indicate *p* < 0.05, *p* < 0.01, and *p* < 0.001, by unpaired two-sided *t* tests. (D) Schematic summarizing differentiation-associated marker changes after *FANCM* loss.

**Figure 5.**
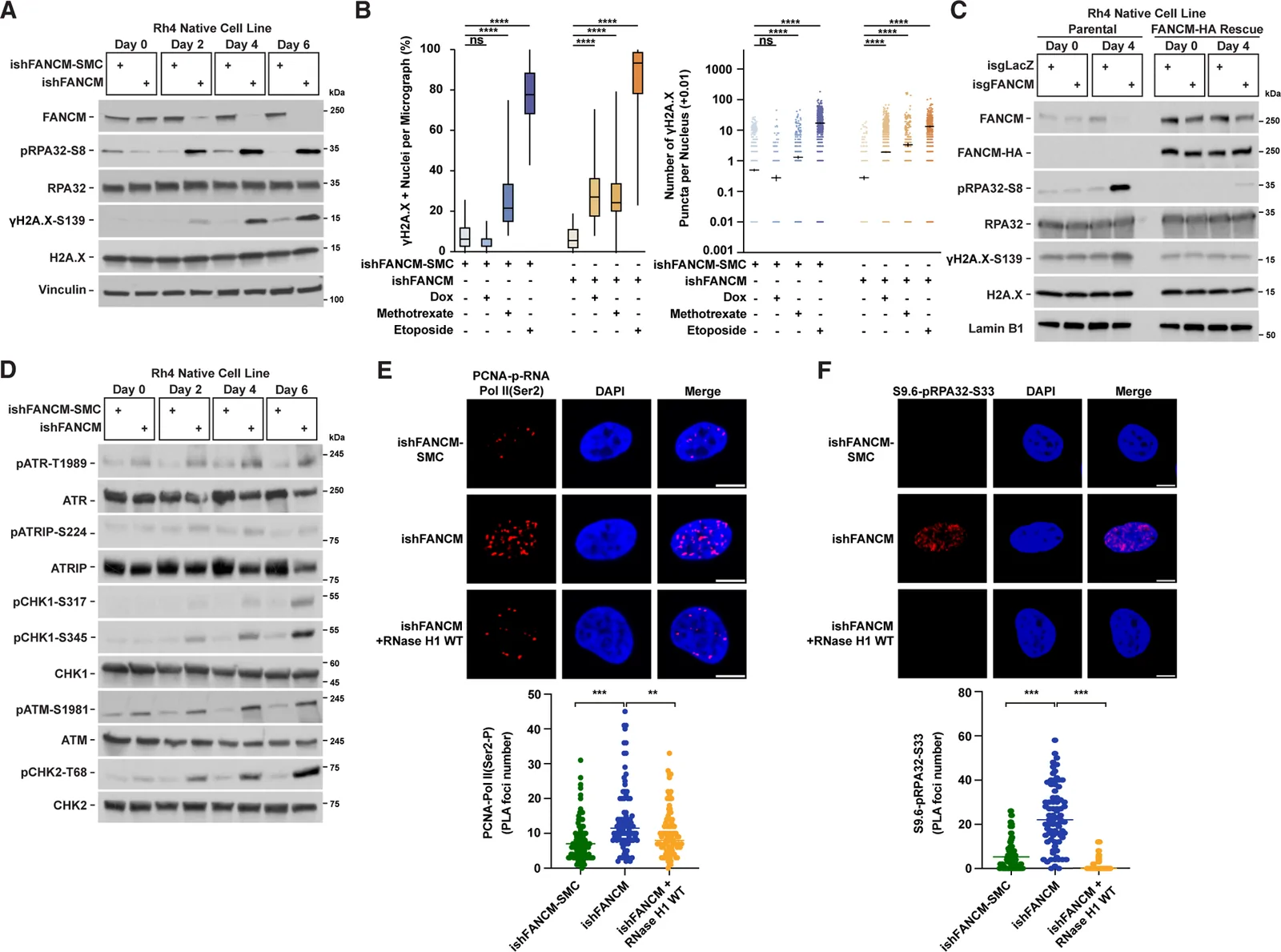
*FANCM* loss increases replication stress, DNA damage, and transcription-replication conflicts in FP RMS cells (A) Western blot time course of replication stress and DNA damage markers after inducible sh*FANCM* compared with seed-matched control in Rh4 native cells. (B) Quantification of γH2A.X-positive nuclei per micrograph and γH2A.X puncta per nucleus after *FANCM* loss, with methotrexate and etoposide positive controls. Boxplots show median, IQR, and range; puncta are represented as mean ± SEM. **** indicates *p* < 0.0001 and NS indicates not significant by one-way ANOVA with Dunnett’s multiple-comparisons test. (C) Western blot analysis showing rescue of pRPA32-S8 and γH2A.X accumulation by ectopic sgRNA-resistant *FANCM* after inducible sgFANCM knockout. (D) Western blot time course of ATR-associated and ATM-associated DDR signaling markers after inducible shFANCM compared with seed-matched control. (E) Representative PCNA and phospho-RNA polymerase II Ser2 PLA images and quantification. Wild-type RNase H1 reduces TRC-associated PLA foci after *FANCM* KD. Scale bars, 5 μm. Each dot represents one nucleus; lines indicate mean ± SEM. ** and *** indicate *p* < 0.01 and *p* < 0.001, respectively, by one-way ANOVA with Tukey’s multiple-comparisons test. (F) Representative S9.6 and pRPA32-S33 PLA images and quantification. S9.6 detects RNA-DNA hybrids, including R-loops, and pRPA32-S33 marks replication stress. Wild-type RNase H1 reduces R-loop-associated pRPA32-S33 PLA foci after *FANCM* KD. Scale bars, 5 μm. Each dot represents one nucleus; lines indicate mean ± SEM. *** indicates *p* < 0.001 by one-way ANOVA with Tukey’s multiple-comparisons test.

**Figure 6.**
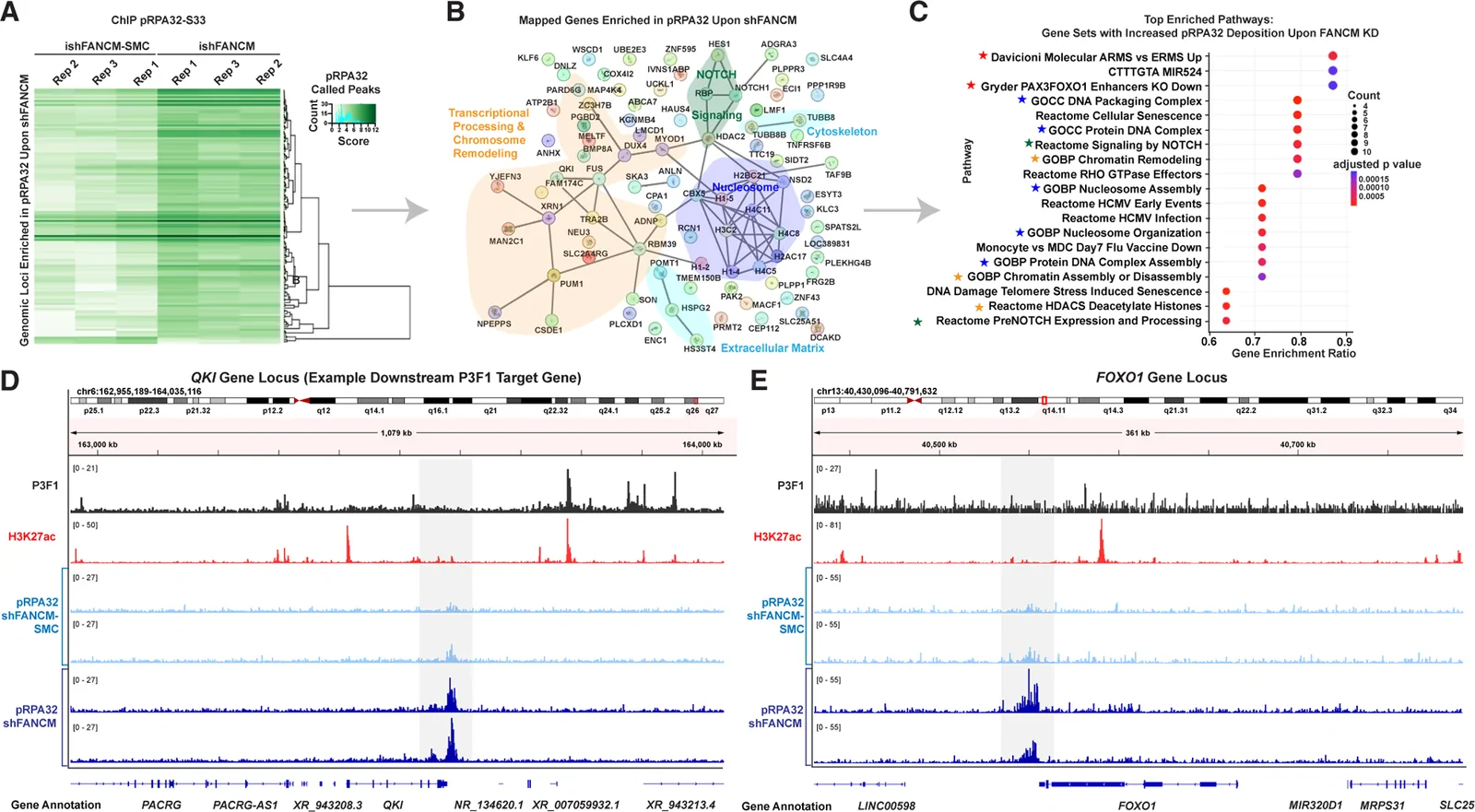
*FANCM* loss enriches chromatin-associated replication stress at PAX3::FOXO1-regulated loci (A) Heatmap of genomic loci enriched for pRPA32-S33 ChIP-seq signal after shFANCM compared with seed-matched control in Rh4 Native cells. (B) STRING network analysis of genes mapped to pRPA32-S33-enriched loci after *FANCM* KD. (C) Gene set overrepresentation analysis of pRPA32-S33-enriched genes. PAX3::FOXO1-related gene sets are highlighted. (D) Genome browser view of the *QKI* locus showing PAX3::FOXO1 occupancy, H3K27ac, and representative pRPA32-S33 ChIP-seq tracks after seed-matched control or *FANCM* KD. (E) Genome browser view of the *FOXO1* locus, used as a proxy for the C-terminal *PAX3::FOXO1* fusion partner. Track coloring is as in (D).

**Figure 7.**
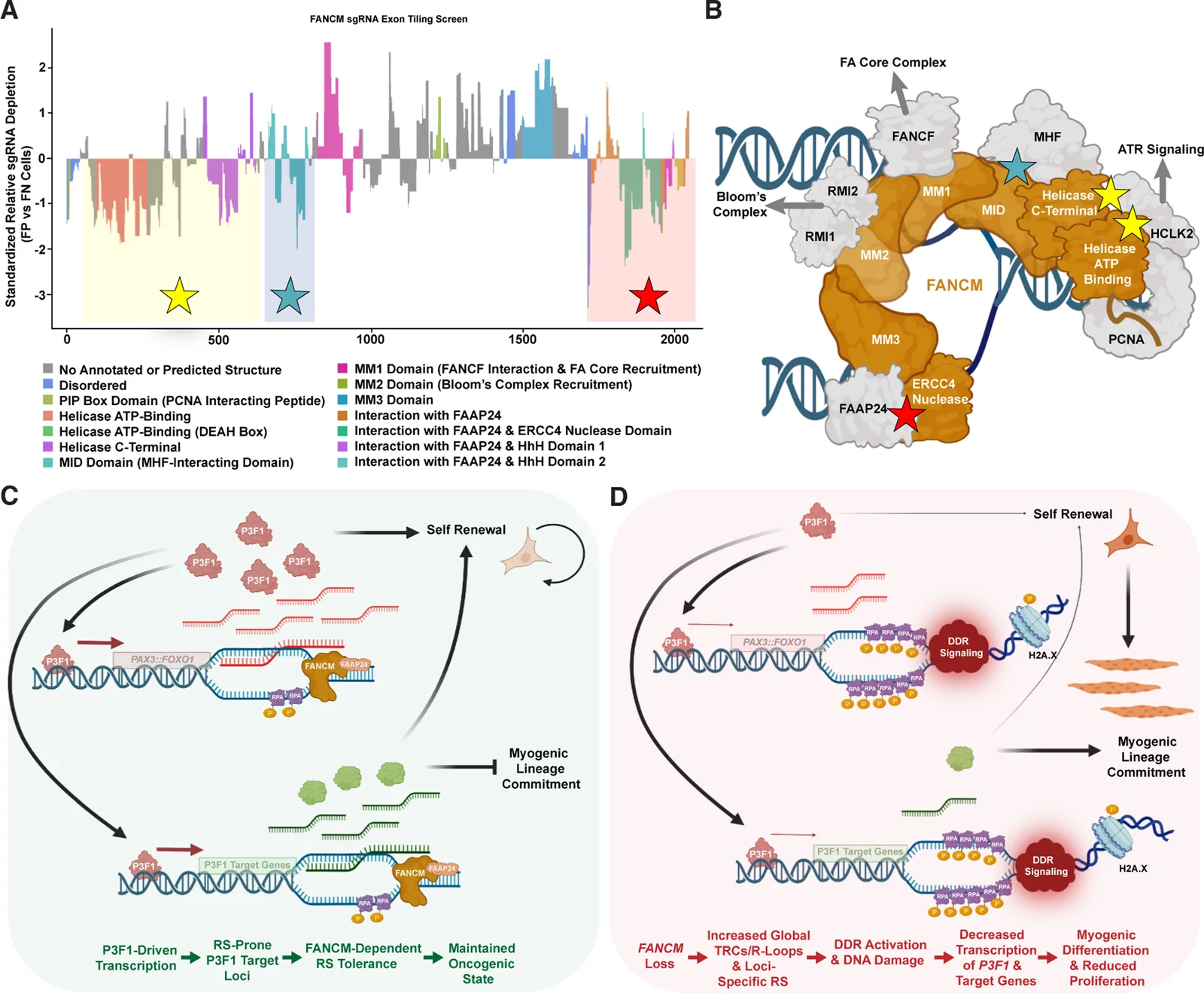
Exon tiling of *FANCM* prioritizes candidate functional hotspots and supports a replication stress tolerance model in FP RMS (A) CRISPR-Cas9 exon-tiling screen across the *FANCM* coding region. Final sgRNA frequencies were compared in FP versus FN cells, smoothed across a sliding window, and standardized. Negative values indicate preferential sgRNA depletion in FP cells. Candidate functional domains are annotated. (B) Schematic of *FANCM* domain architecture and selected functional interaction partners. Stars correspond to the major FP-selective regions nominated in (A). (C) Proposed model in *FANCM*-proficient FP RMS cells: PAX3::FOXO1-driven transcription creates RS-prone fusion-program loci that are tolerated through *FANCM*-dependent fork stabilization and RS tolerance, maintaining the oncogenic state. (D) Proposed model after *FANCM* loss: global TRCs and R-loops increase, locus-enriched RS increases, DDR signaling and DNA damage accumulate, fusion-program transcription decreases, and cells undergo myogenic differentiation with reduced proliferative capacity.

**Table:** KEY RESOURCES TABLE

REAGENT or RESOURCE	SOURCE	IDENTIFIER

Antibodies		

Rabbit anti-ATM (D2E2)	Cell Signaling Technology	Cat#2873; RRID: AB_2062659
Rabbit anti-phospho-ATM (Ser1981) (D25E5)	Cell Signaling Technology	Cat#13050; RRID: AB_2798100
Rabbit anti-ATR (E1S3S)	Cell Signaling Technology	Cat#13934; RRID: AB_2798347
Rabbit anti-phospho-ATR (Thr1989)	GeneTex	Cat#GTX128145; RRID: AB_2687562
Rabbit anti-ATRIP (E2I9A)	Cell Signaling Technology	Cat#97687; RRID: N/A
Rabbit anti-phospho-ATRIP (Ser224)	Cell Signaling Technology	Cat#5161; RRID: N/A
Rabbit anti-beta-actin (13E5)	Cell Signaling Technology	Cat#4970; RRID: AB_2223172
Rabbit anti-Caspase 3/cleaved Caspase 3 (Asp175)	Cell Signaling Technology	Cat#9661; RRID: AB_2341188
Mouse anti-CHK1 (2G1D5)	Cell Signaling Technology	Cat#2360; RRID: AB_2080320
Rabbit anti-phospho-CHK1 (Ser317)	Cell Signaling Technology	Cat#2344; RRID: AB_331488
Rabbit anti-phospho-CHK1 (Ser345) (133D3)	Cell Signaling Technology	Cat#2348; RRID: AB_331212
Rabbit anti-CHK2 (D9C6)	Cell Signaling Technology	Cat#6334; RRID: AB_11178526
Rabbit anti-phospho-CHK2 (Thr68) (C13C1)	Cell Signaling Technology	Cat#2197; RRID: AB_2080501
Mouse anti-CKM (1E3)	Abnova	Cat#H00001158-M02; RRID: AB_534819
Mouse anti-DNA-RNA hybrid (clone S9.6)	Home-made from Sigma-Aldrich/Millipore material	Cat#MABE1095; RRID: AB_2861387
Mouse anti-FANCM (CV5.1)	Novus Biologicals	Cat#NBP2–50418; RRID: AB_2716711
Mouse anti-FLAG M2	Sigma	Cat#F3165; RRID: AB_259529
Rabbit anti-FOXO1 (C29H4)	Cell Signaling Technology	Cat#2880; RRID: AB_2106495
Rabbit anti-H3K27ac	Active Motif	Cat#39133; RRID: AB_2561016
Rabbit anti-Histone H2A.X (D17A3)	Cell Signaling Technology	Cat#7631; RRID: AB_10860771
Rabbit anti-phospho-Histone H2A.X (Ser139) (20E3)	Cell Signaling Technology	Cat#9718; RRID: AB_2118009
HRP-conjugated secondary antibodies	Cell Signaling Technology	Cat#7074; RRID: AB_2099233; Cat#7076; RRID: AB_330924
Anti-Lamin B1	Cell Signaling Technology	Cat#13435; RRID: AB_2737428
Goat anti-mouse Alexa Fluor 647	Thermo Fisher Scientific	Cat#A-21235; RRID: AB_2535804
Mouse anti-MYH4/MYHC (MF20)	Developmental Studies Hybridoma Bank	Cat#MF20; RRID: AB_2147781
Rabbit anti-MYL1 (EPR9934(B))	Abcam	Cat#ab151749; RRID: N/A
Rabbit anti-MYOD1 (D8G3)	Cell Signaling Technology	Cat#13812; RRID: AB_2798320
Mouse anti-MYOG (5FD)	Santa Cruz Biotechnology	Cat#sc-52903; RRID: AB_784707
Rabbit anti-PARP/cleaved PARP (Asp214) (D64E10)	Cell Signaling Technology	Cat#5625; RRID: AB_10699459
Mouse anti-PAX3 (GT1210)	Thermo Fisher Scientific	Cat#MA5–31583; RRID: AB_2787210
Mouse anti-PCNA (PC10)	Santa Cruz Biotechnology	Cat#sc-56; RRID: AB_628110
Goat anti-rabbit Alexa Fluor 488	Thermo Fisher Scientific	Cat#A-11008; RRID: AB_143165
Goat anti-rabbit Alexa Fluor 647	Thermo Fisher Scientific	Cat#A-21244; RRID: AB_2535812
Rabbit anti-RNA polymerase II CTD phospho-Ser2	Novus Biologicals	Cat#NB100–1805; RRID: AB_10001499
Rabbit anti-RPA32	Cell Signaling Technology	Cat#52448; RRID: AB_2750889
Rabbit anti-phospho-RPA32 (Ser33)	Bethyl Laboratories	Cat#A300–246A; RRID: AB_2180847
Rabbit anti-phospho-RPA32 (Ser33) (PLA)	Novus Biologicals	Cat#NB100–544; RRID: AB_10002227
Rabbit anti-phospho-RPA32 (Ser8)	Cell Signaling Technology	Cat#83745; RRID: AB_2800029
Rabbit anti-vinculin (E1E9V)	Cell Signaling Technology	Cat#13901; RRID: AB_2728768

Bacterial and virus strains		

Endura electrocompetent cells	Lucigen	Cat#LGC6024221
Lentivirus generated for sgRNA, shRNA, FANCM rescue, RNase H1, and Cas9 delivery	This paper	N/A
One Shot Stbl3 chemically competent E. coli	Invitrogen	Cat#C737303

Chemicals, peptides, and recombinant proteins		

Actinomycin D	Thermo Fisher Scientific/Gibco	Cat#11805017
Alexa Fluor 488 phalloidin	Thermo Fisher Scientific	Cat#A12379
AZD6738 (ceralasertib)	MedChemExpress	Cat#HY-19323
BAY1895344 (elimusertib)	MedChemExpress	Cat#HY-101566
Blasticidin S HCl	Gibco	Cat#A1113903
DAPI	Thermo Fisher Scientific	Cat#62248
Doxycycline hyclate	VWR; Thermo Fisher Scientific; Inotiv	Cat#24390–14–5 (*in vitro* use); Cat#446060250 (*in vivo* oral gavage); Cat#TD01306 (chow)
Etoposide	MedChemExpress	Cat#HY-13629
G418 sulfate	Gibco	Cat#10131035
Hoechst 33342	Thermo Fisher Scientific	Cat#62249
Lipofectamine 3000	Invitrogen	Cat#L3000150
Lipofectamine LTX with PLUS reagent	Invitrogen	Cat#15338100
Matrigel	Corning	Cat#356234
Methotrexate	MedChemExpress	Cat#HY-14519
Methylcellulose	Fisher Scientific	Cat#M352–500
PIP-199	MedChemExpress	Cat#HY-124325
ProLong Gold mounting medium	Thermo Fisher Scientific	Cat#P36934
Puromycin dihydrochloride	GoldBio	Cat#P-600–100

Critical commercial assays		

Arcturus PicoPure DNA Extraction Kit	Thermo Fisher Scientific	Cat#KIT0103
CellTiter-Glo Luminescent Cell Viability Assay	Promega	Cat#G7573
DNeasy Blood & Tissue Kit	Qiagen	Cat#69504
Duolink *In Situ* PLA detection reagents	Sigma-Aldrich	Cat#DUO92008
Duolink *In Situ* PLA probes	Sigma-Aldrich	Cat#DUO92001; Cat#DUO92002
eBioscience Annexin V Apoptosis Detection Kits	Invitrogen	Cat#88–8007–72
eBioscience Intracellular Staining Buffer Set	Invitrogen	Cat#88–8824–00
GoTaq 1-Step RT-qPCR System	Promega	Cat#A6020
KAPA mRNA HyperPrep Kit	Roche	Cat#KK8581
MycoAlert Mycoplasma Detection Kit	Lonza	Cat#LT07–318
NEBNext Multiplex Oligos for Illumina	New England Biolabs	Cat#E7600S
NEBNext Ultra II DNA Library Prep Kit for Illumina	New England Biolabs	Cat#E7645S
NEBNext Ultra II Q5 Master Mix	New England Biolabs	Cat#M0544X
QIAshredder	Qiagen	Cat#79656
Quant-iT dsDNA High Sensitivity Assay Kit	Thermo Fisher Scientific	Cat#Q33120
Quick DNA Kit	Zymo Research	Cat#D3025
Quick-DNA Midiprep Plus Kit	Zymo Research	Cat#D4075
Quick-RNA Miniprep Plus Kit	Zymo Research	Cat#R1058
RNeasy Kit	Qiagen	Cat#74106

Deposited data		

Raw and processed ChIP-seq data	This paper	GEO: GSE292876
Raw and processed CRISPR/Cas9 screening data	This paper	GEO: GSE292882
Raw and processed RNA-seq data	This paper	GEO: GSE292872

Experimental models: Cell lines		

Human: 293T AAVpro	Takara Bio	Cat#632273
Human: immortalized myoblasts (Dbt cell lines) with/without inducible PAX3::FOXO1	Corinne Linardic	STR profiled; available from lead contact upon reasonable request
Human: RD FN RMS cell line	Corinne Linardic	STR profiled; available from lead contact upon reasonable request
Human: Rh4 Clone FP RMS cell line; endogenous PAX3::FOXO1-FLAG	Corinne Linardic; Laubscher et al.	STR profiled; available from lead contact upon reasonable request
Human: Rh4 Native FP RMS cell line	Corinne Linardic	STR profiled; available from lead contact upon reasonable request
Human: Rh28 FP RMS cell line	Corinne Linardic	STR profiled; available from lead contact upon reasonable request
Human: Rh30 FP RMS cell line	Corinne Linardic	STR profiled; available from lead contact upon reasonable request
Human: SMS-CTR FN RMS cell line	Corinne Linardic	STR profiled; available from lead contact upon reasonable request
Human: RMS derivative lines generated in this study (Cas9, sgRNA, shRNA, FANCM-rescue, RNase H1 derivatives)	This paper	Available from lead contact upon reasonable request

Experimental models: Organisms/strains		

Mouse: NSG mice, 6-weeks-old, female	The Jackson Laboratory	NOD.Cg-Prkdc^scid^ Il2rg^tm1Wjl^/SzJ; Strain #:005557; RRID: IMSR_JAX:005557

Oligonucleotides		

Beta-actin RT-qPCR primers	IDT	F: AGAGCTACGAGCTGCCTGAC; R: AGCACTGTGTTGGCGTACAG
*FANCM* RT-qPCR primers	IDT	F: AGGGATGGAATGAGGCAAAGT; R: AGTGGTTGATTCTTGAGCTGGT
*GAPDH* RT-qPCR primers	IDT	F: GGAGCGAGATCCCTCCAAAAT; R: GGCTGTTGTCATACTTCTCATGG
*PAX3::FOXO1* RT-qPCR primers	IDT	F: CATGGATTTTCCAGCTATACAGAC; R: GTTCCTTCATTCTGCACACG
Individual shRNA sequences	TRC; this paper	See STAR Methods
Individual sgRNA sequences	TKOv3; MinLibCas9; CRISPick; this paper	See STAR Methods
*FANCM* TIDE and amplicon sequencing primers	This paper	See STAR Methods

Recombinant DNA		

Doxycycline-inducible GFP-tagged RNase H1-WT and RNase H1-D210N expression constructs in pInducer20	Lee Zou laboratory	N/A
lentiCas9-Blast	Addgene	RRID: Addgene_52962
lentiGuide-Puro	Addgene	RRID: Addgene_52953
pLenti CMV *Neo* DEST (705–1)	Addgene	RRID: Addgene_17392
pLV-U6-gRNA-hUbC-mCherry-P2A-PuroR (enh-sgRNA-mCherry-Puro)	Charles Gersbach	N/A
pMD2.G	Addgene	RRID: Addgene_12259
pRDA_355	Addgene	RRID: Addgene_187159
psPAX2	Addgene	RRID: Addgene_12260
sgRNA-resistant C-terminal HA-tagged *FANCM* rescue construct	This paper	See Figure S3B; Table S8
Tet-pLKOpuro	Addgene	RRID: Addgene_21915
Toronto KnockOut CRISPR Library, Version 3 (TKOv3)	Addgene	RRID: Addgene_90294

Software and algorithms		

BWA	Li and Durbin^64^	RRID: SCR_010910
CellProfiler 4.2.8	Broad Institute	RRID: SCR_007358
clusterProfiler	Yu et al.^65^	RRID: SCR_016884
ColabFold2	Mirdita et al.^66^	RRID: SCR_025453
DECODR	Bloh et al.^67^	https://www.decodrinc.com/
deepTools	Ramirez et al.^68^	RRID: SCR_016366
DESeq2	Love et al.^69^	RRID: SCR_015687
DiffBind	Ross-Innes et al.^50^	RRID: SCR_012918
FastQC	Andrews	RRID: SCR_014583
featureCounts	Liao et al.^70^	RRID: SCR_012919
fgsea	Korotkevich et al.^71^	RRID: SCR_020938
GraphPad Prism 11	GraphPad Software	RRID: SCR_002798
HISAT2	Kim et al.^72^	RRID: SCR_015530
IGV	Broad Institute	RRID: SCR_011793
ImageJ	NIH	RRID: SCR_003070
JMP Pro	JMP Statistical Discovery	RRID: SCR_014242
MACS2	Zhang et al.^14^	RRID: SCR_013291
MAGeCK	Li et al.^31^	RRID: SCR_025016
MAGeCK-VISPR	Li et al.^73^	N/A
Open-source PyMOL	Schrösdinger/community	RRID: SCR_000305
Picard	Broad Institute	RRID: SCR_006525
R 4.4.2	R Foundation	RRID: SCR_001905
SAMtools	Li et al.^74^	RRID: SCR_002105
STRING	Szklarczyk et al.^33^	RRID: SCR_005223
Trim Galore	Babraham Bioinformatics	RRID: SCR_011847

## Highlights

- FANCM is a selective dependency in PAX3::FOXO1-positive RMS
- FANCM depletion suppresses the fusion oncogenic program
- FANCM buffers transcription-linked replication stress at PAX3::FOXO1 target loci
- FANCM domain mapping prioritizes candidate regions for future therapeutic studies
