# Supplementary material for: FANCM is required for the PAX3::FOXO1-driven oncogenic program in rhabdomyosarcoma
Source: Cell Rep. Author manuscript; Available in PMC 2026 Nov 3. (PMC13728430; doi:10.1016/j.celrep.2026.117960)
Supplement: Supplementary file 1 [file NIHMS2212244-supplement-1.pdf]

Cell Reports, Volume *45*

Supplemental information

FANCM is required for the PAX3::FOXO1-driven oncogenic program in rhabdomyosarcoma

Christopher D. Delaney, Min Lu, Seth P. Zimmerman, Yingying Meng, Camille Vaganay, Christian G. Cerda-Smith, Annie Liu, Jacob P. Hoj, Shane T. Killarney, Kerry Dillon, Amy E. Stewart, Kevin Huang, Julia D. Caci, Olivia White, Alexandre Puissant, David E. Root, Jack F. Shern, Christine E. Eyler, Chun-Wei David Chen, Christopher M. Counter, Corinne M. Linardic, Lee Zou, and Kris C. Wood

Figure S1

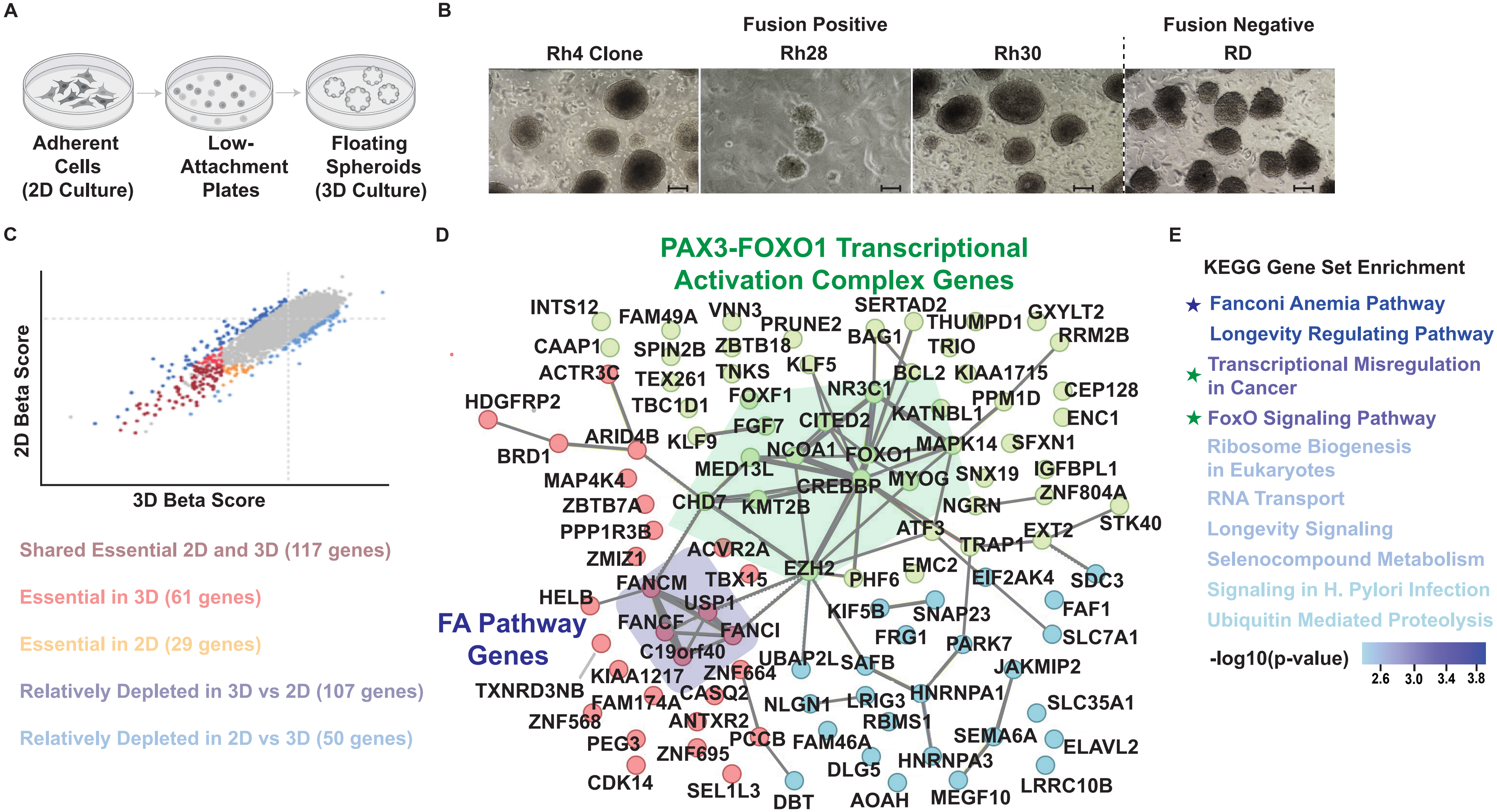

Figure S1. Genome-scale CRISPR/Cas9 screening in 2D and 3D culture nominates FA pathway genes in FP RMS, related to Figure 1.
(A) Schematic of the 2D adherent and 3D spheroid culture workflow.
(B) Representative brightfield images of FP and FN RMS spheroids. Scale bars, 100 µm.
(C) Genome-scale screen beta scores highlighting genes shared between 2D and 3D culture or preferentially depleted in one condition.
(D) STRING network analysis of top FP-specific genes from the custom essentiality screen, highlighting FA pathway and PAX3::FOXO1 transcriptional activation complex genes.
(E) KEGG gene set enrichment analysis of top FP-specific genes.

Figure S2

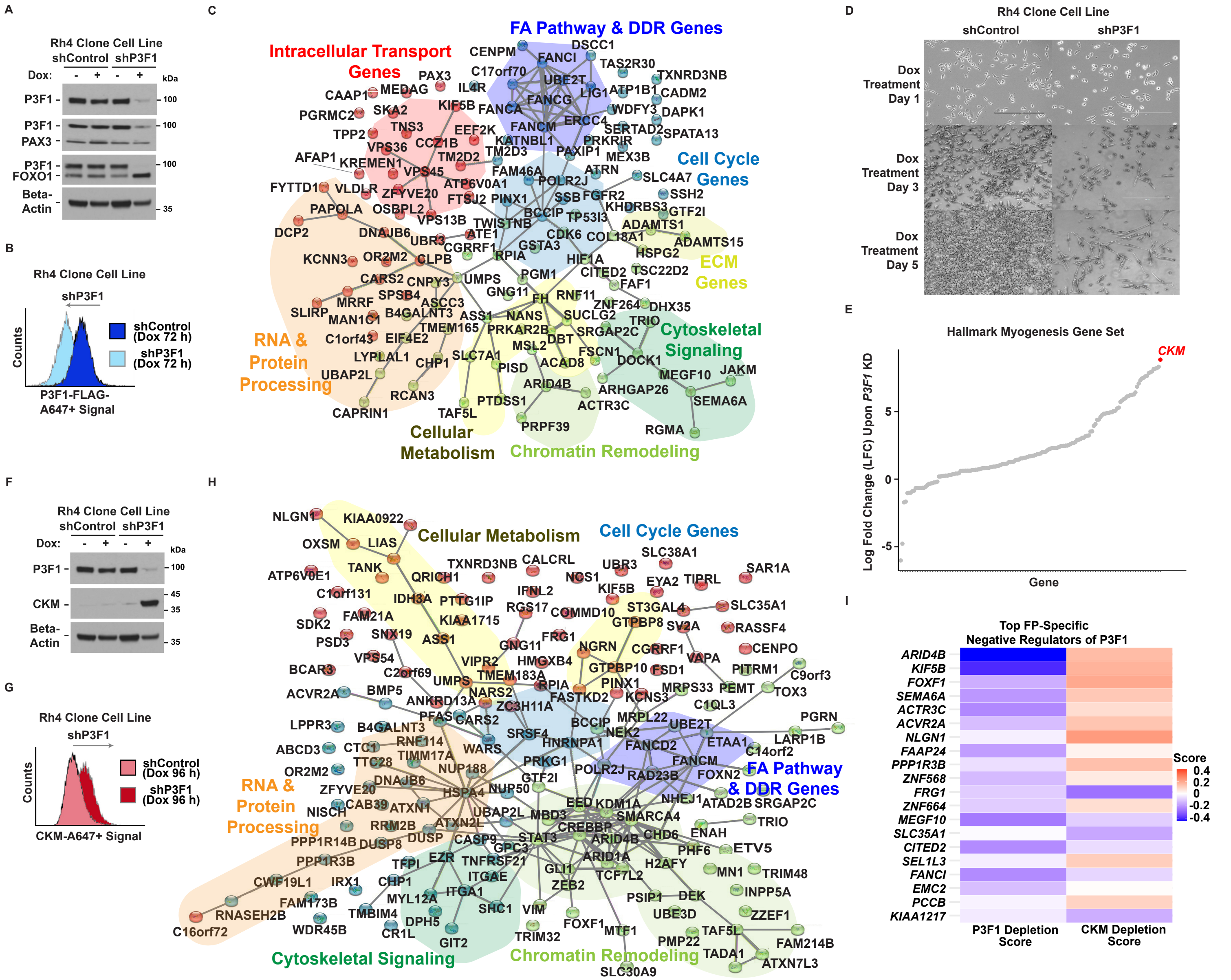

Figure S2. Validation and supporting analyses for the PAX3::FOXO1-FLAG and CKM flow-based screens, related to Figure 1.
(A) Western blot validation of breakpoint-targeting shP3F1 in Rh4 Clone cells. Antibody probes from top to bottom: anti-FLAG, anti-PAX3, anti-FOXO1.
(B) Flow cytometry detection of endogenous PAX3::FOXO1-FLAG signal after shControl or shP3F1 induction.
(C) STRING network analysis of PAX3::FOXO1-FLAG flow-screen hits.
(D) Sequential brightfield images after shControl or shP3F1 induction in Rh4 Clone cells. Scale bars, 400 µm.
(E) Ranked log fold changes for Hallmark Myogenesis genes after PAX3::FOXO1 KD. CKM is highlighted.
(F) Western blot validation of CKM induction after PAX3::FOXO1 KD. Anti-FLAG was used to detect P3F1.
(G) Flow cytometry detection of CKM after shControl or shP3F1 induction.
(H) STRING network analysis of CKM flow-screen hits.
(I) Heatmap of top FP-specific negative regulators of PAX3::FOXO1 showing P3F1 and CKM depletion scores.

Figure S3

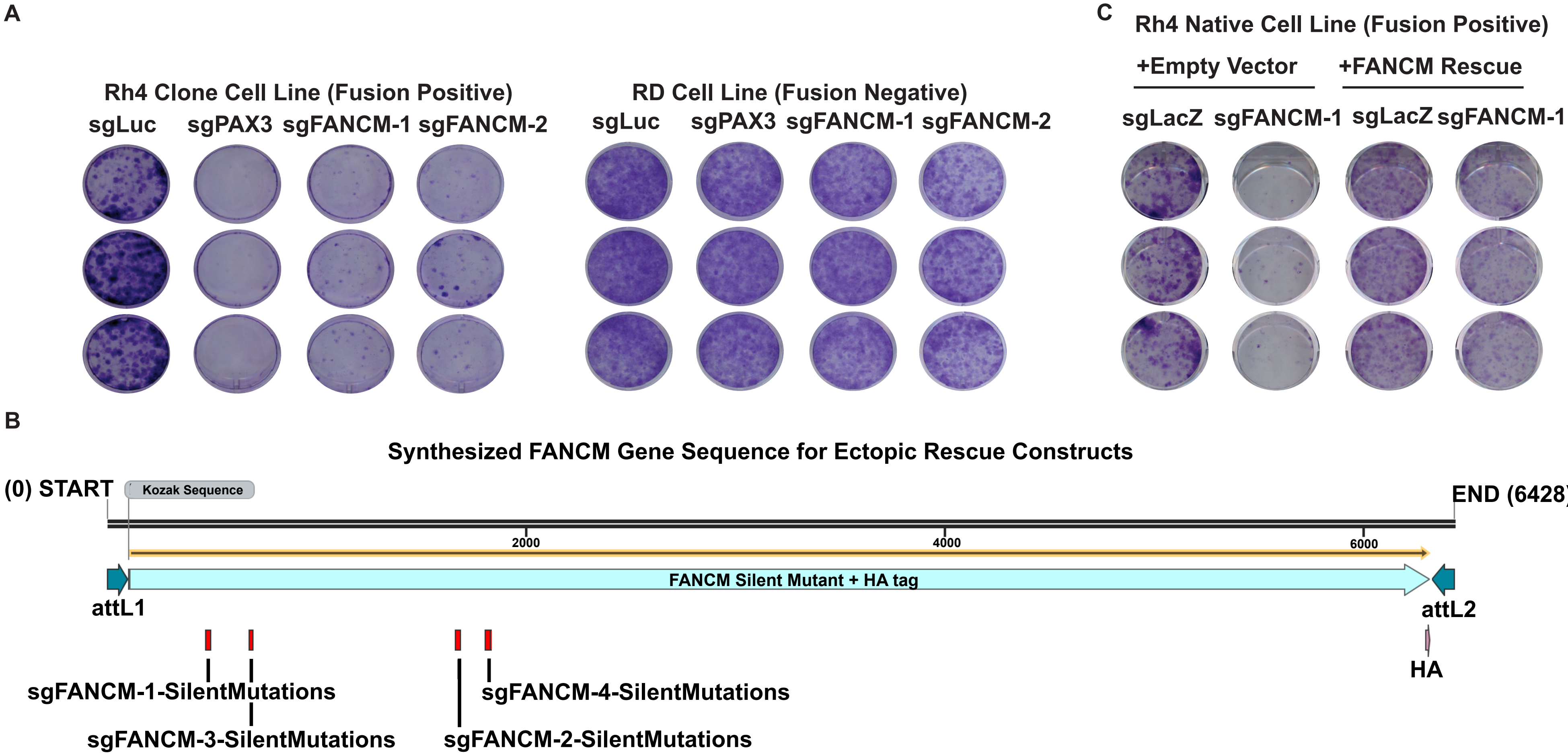

Figure S3. *FANCM* knockout clonogenic validation and ectopic rescue construct design, related to Figure 2.

(A) Clonogenic assays after sgRNA-mediated *FANCM* knockout in FP Rh4 Clone and FN RD cells.

(B) Schematic of the synthesized sgRNA-resistant *FANCM* rescue construct with silent mutations and C-terminal HA tag.

(C) Clonogenic rescue of the *FANCM* knockout phenotype in Rh4 Native cells expressing sgRNA-resistant *FANCM*.

Figure S4

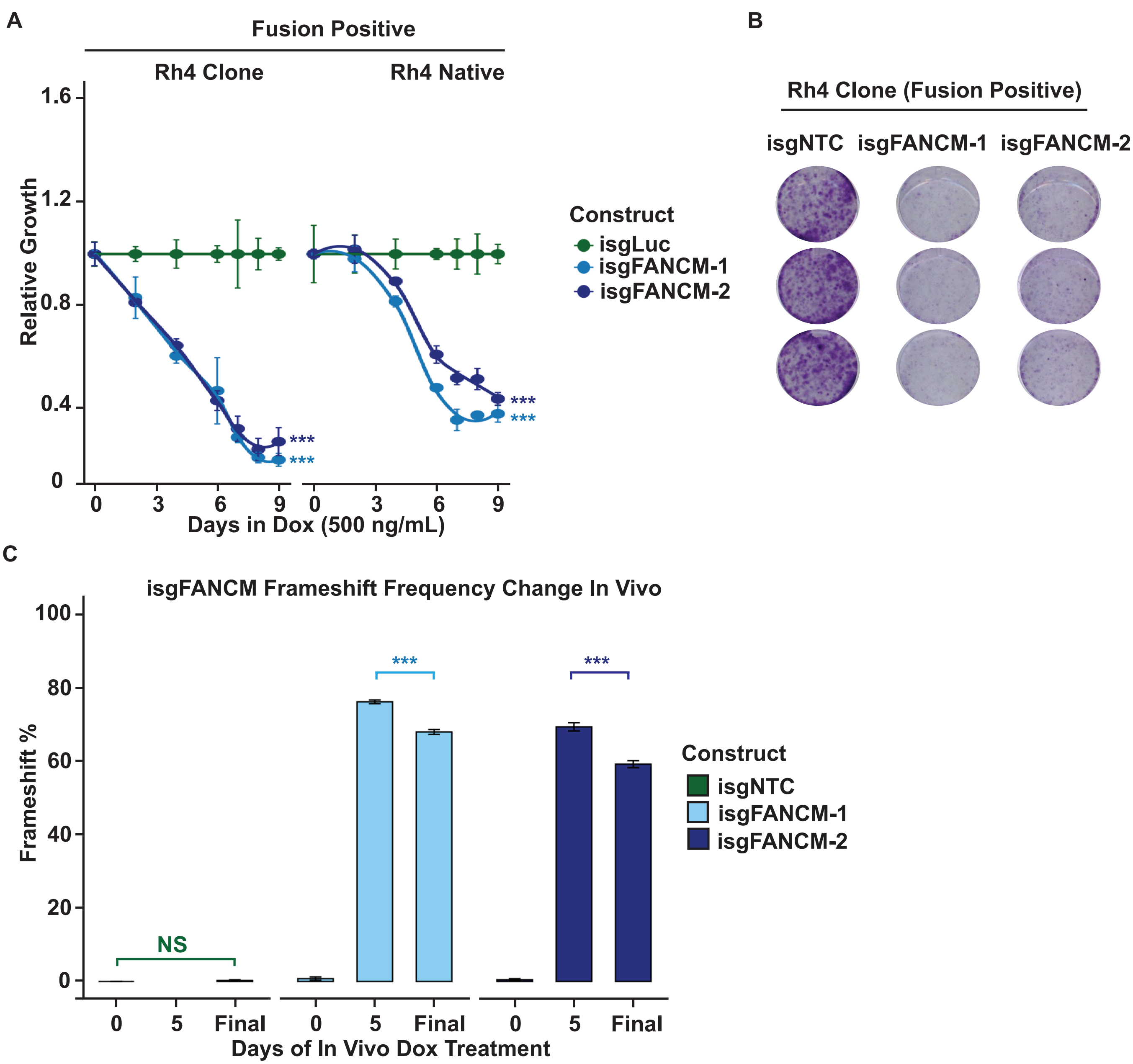

Figure S4. Characterization of the inducible sgRNA-mediated *FANCM* knockout system, related to Figure 2.
(A) Growth curves after inducible sgRNA-mediated *FANCM* knockout in FP RMS cell lines. Data are represented as mean ± SEM. *** indicates p < 0.001 by two-way ANOVA with Dunnett's multiple-comparisons test.
(B) Clonogenic assays after inducible sgFANCM or control sgRNA expression in Rh4 Clone cells. Data are represented as mean ± SEM.
(C) TIDE analysis of *FANCM* frameshift frequency in Rh4 Clone xenograft tumors after *in vivo* doxycycline induction. Data are represented as mean ± SEM. *** indicates p < 0.001 and NS indicates not significant by two-way ANOVA with Sidak's multiple-comparisons test.

Figure S5

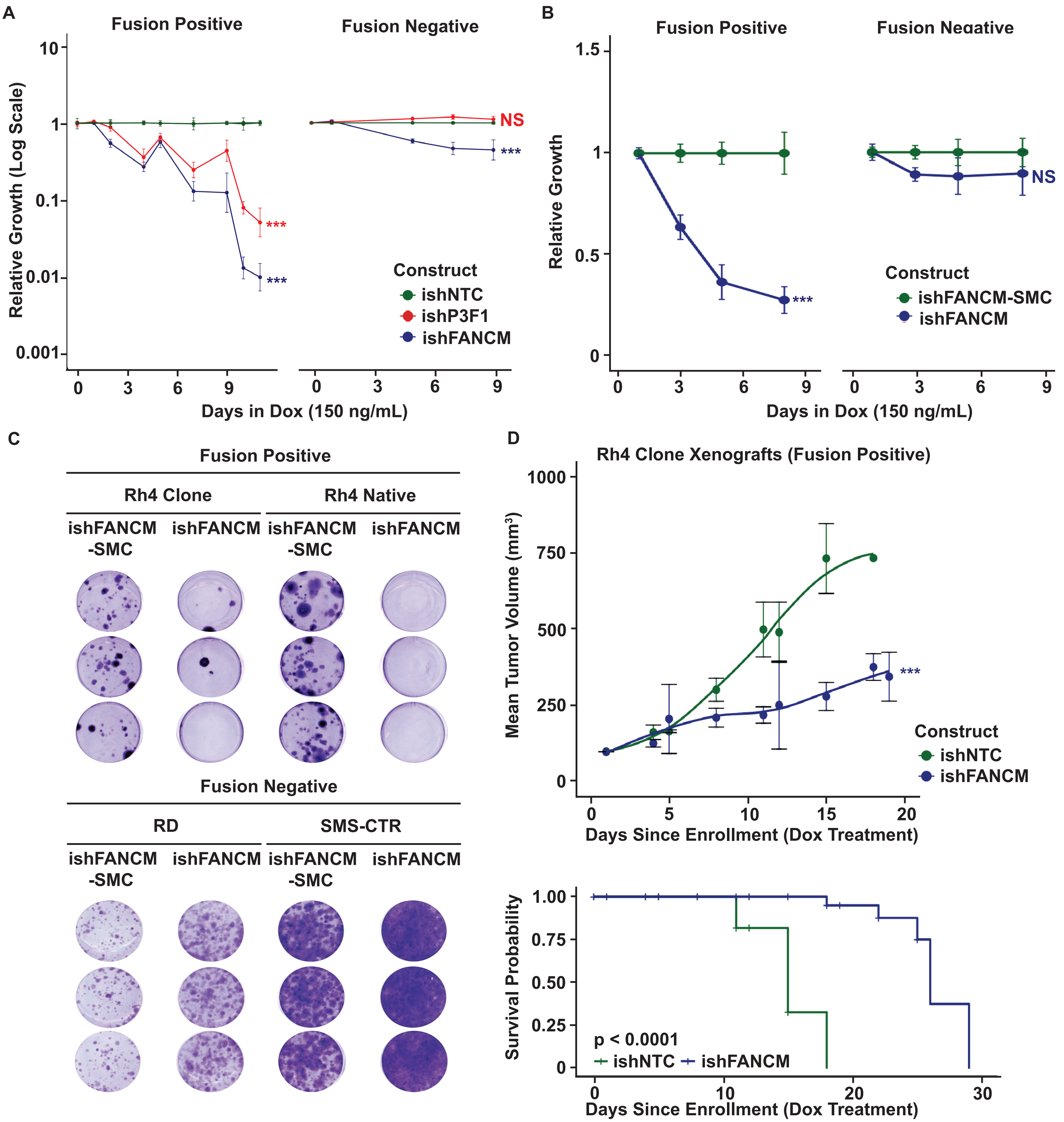

Figure S5. Characterization of inducible shRNA-mediated *FANCM* knockdown *in vitro* and *in vivo*, related to Figure 2.
(A) Growth curves after inducible shFANCM, shP3F1, or non-targeting control in FP and FN RMS cells. Data are represented as mean ± SEM. *** indicates $p < 0.001$ and NS indicates not significant by two-way ANOVA with Dunnett's multiple-comparisons test.
(B) Growth curves after inducible shFANCM compared with seed-matched control in FP and FN RMS cells. Data are represented as mean ± SEM. *** indicates $p < 0.001$ and NS indicates not significant by two-way ANOVA with Dunnett's multiple-comparisons test.
(C) Clonogenic assays after inducible shFANCM or seed-matched control in FP and FN RMS cells.
(D) Rh4 Clone xenograft tumor growth and Kaplan-Meier survival after inducible shFANCM or non-targeting control. Tumor volumes are represented as mean ± SEM. *** indicates $p < 0.001$ by ordinary least-squares polynomial regression. Survival was compared by log-rank test.

Figure S6

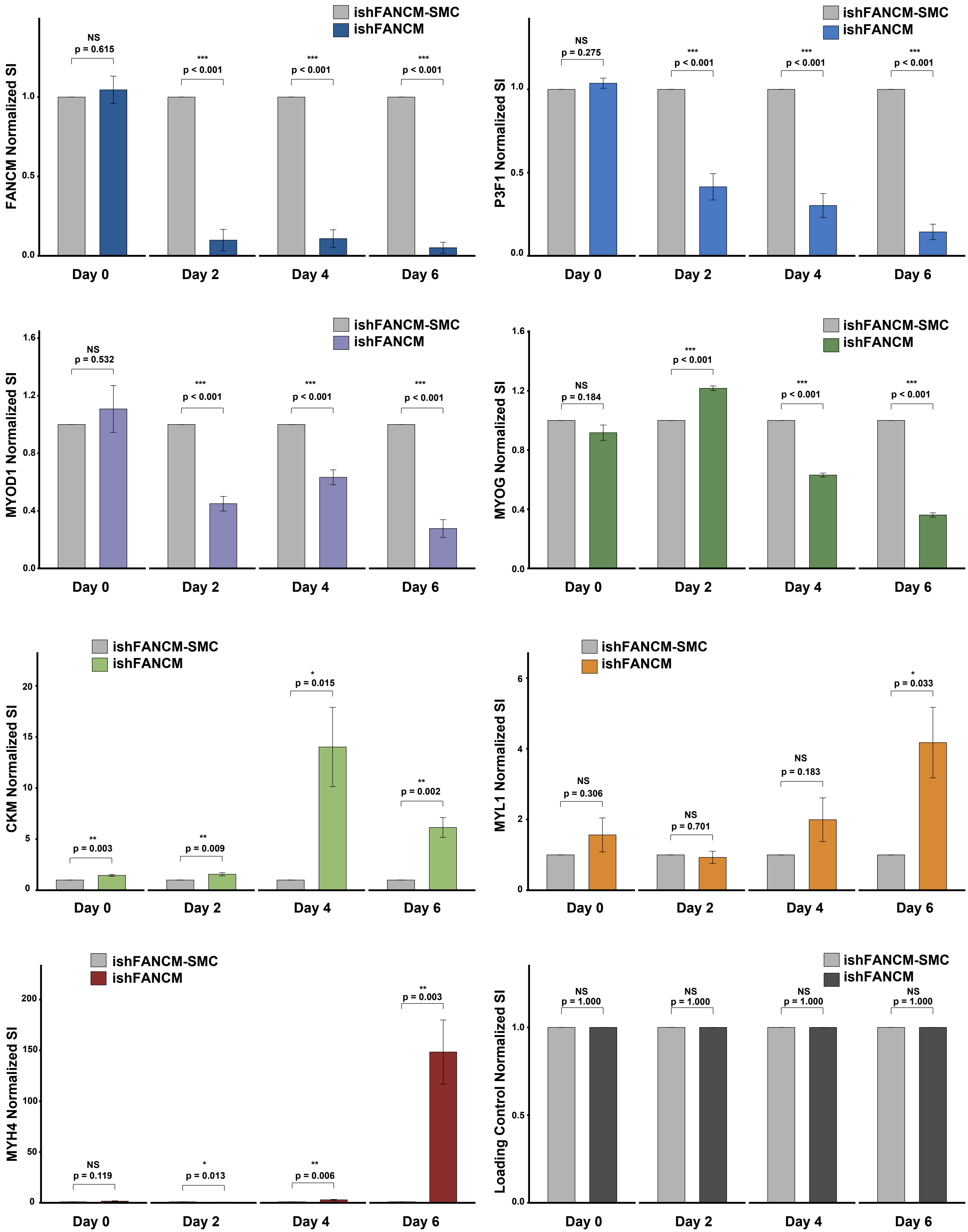

Figure S6. Quantification of myogenic differentiation marker Western blot time course after *FANCM* KD, related to Figure 4. Densitometric quantification of FANCM, PAX3::FOXO1, MYOD1, MYOG, CKM, MYL1, MYH4, and loading control signals from the Western blot time course in **Figure 4C**. Signal intensities were normalized to loading control and day-matched seed-matched control. Data are represented as mean ± SEM. *, **, and *** indicate $p < 0.05$, $p < 0.01$, and $p < 0.001$, respectively, and NS indicates not significant, by unpaired two-sided t tests.

Figure S7

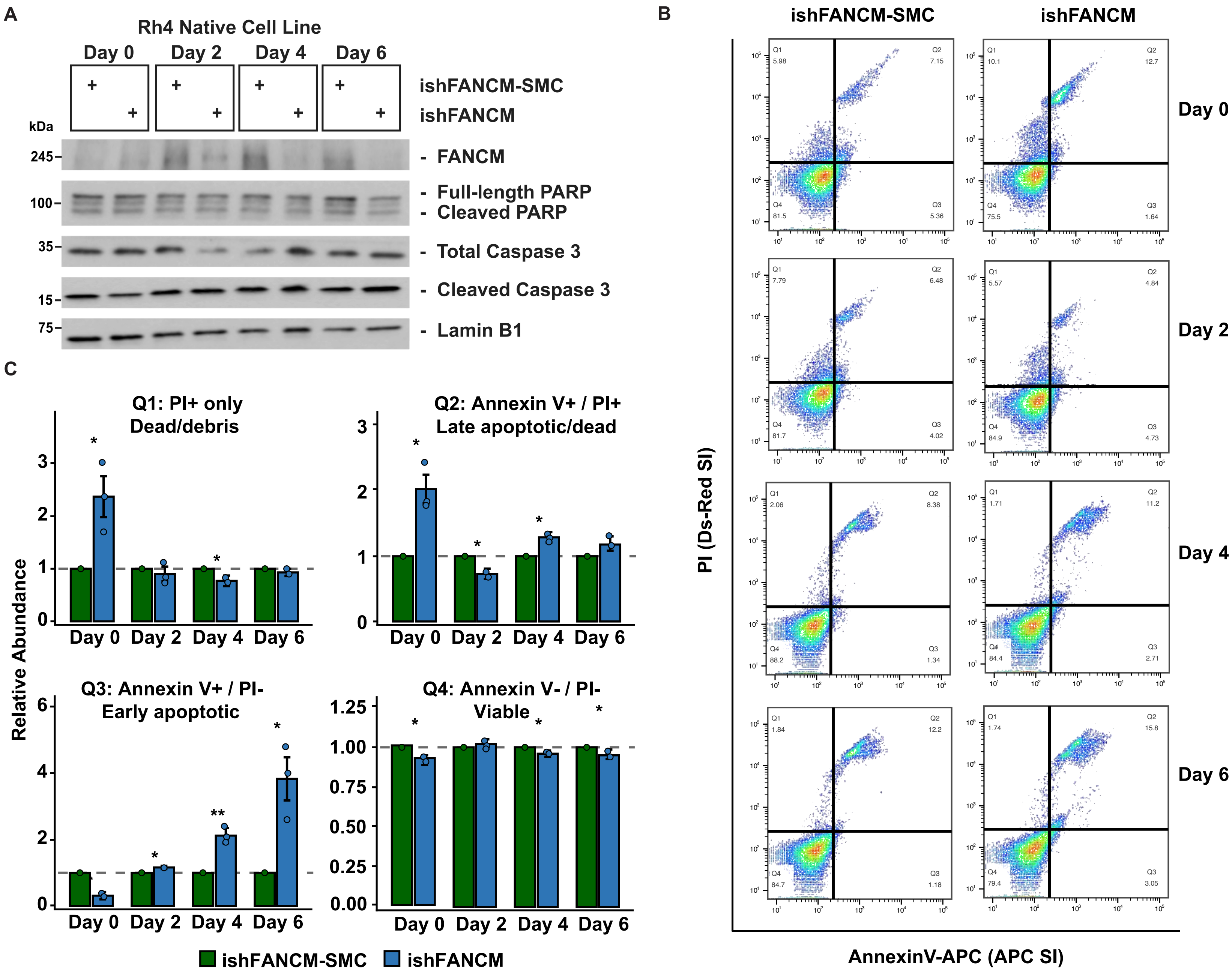

Figure S7. *FANCM* KD does not robustly activate apoptotic pathway markers in adherent FP RMS cells, related to Figure 4.
(A) Western blot time course of FANCM, PARP, Caspase 3, and Lamin B1 after inducible shFANCM or seed-matched control in Rh4 Native cells.
(B) Representative Annexin V and PI flow cytometry plots across the inducible shFANCM time course.
(C) Quantification of Annexin V and PI quadrants relative to seed-matched control. Data are represented as mean ± SEM. * and ** indicate $p < 0.05$ and $p < 0.01$, respectively, by unpaired two-sided t tests.

Figure S8

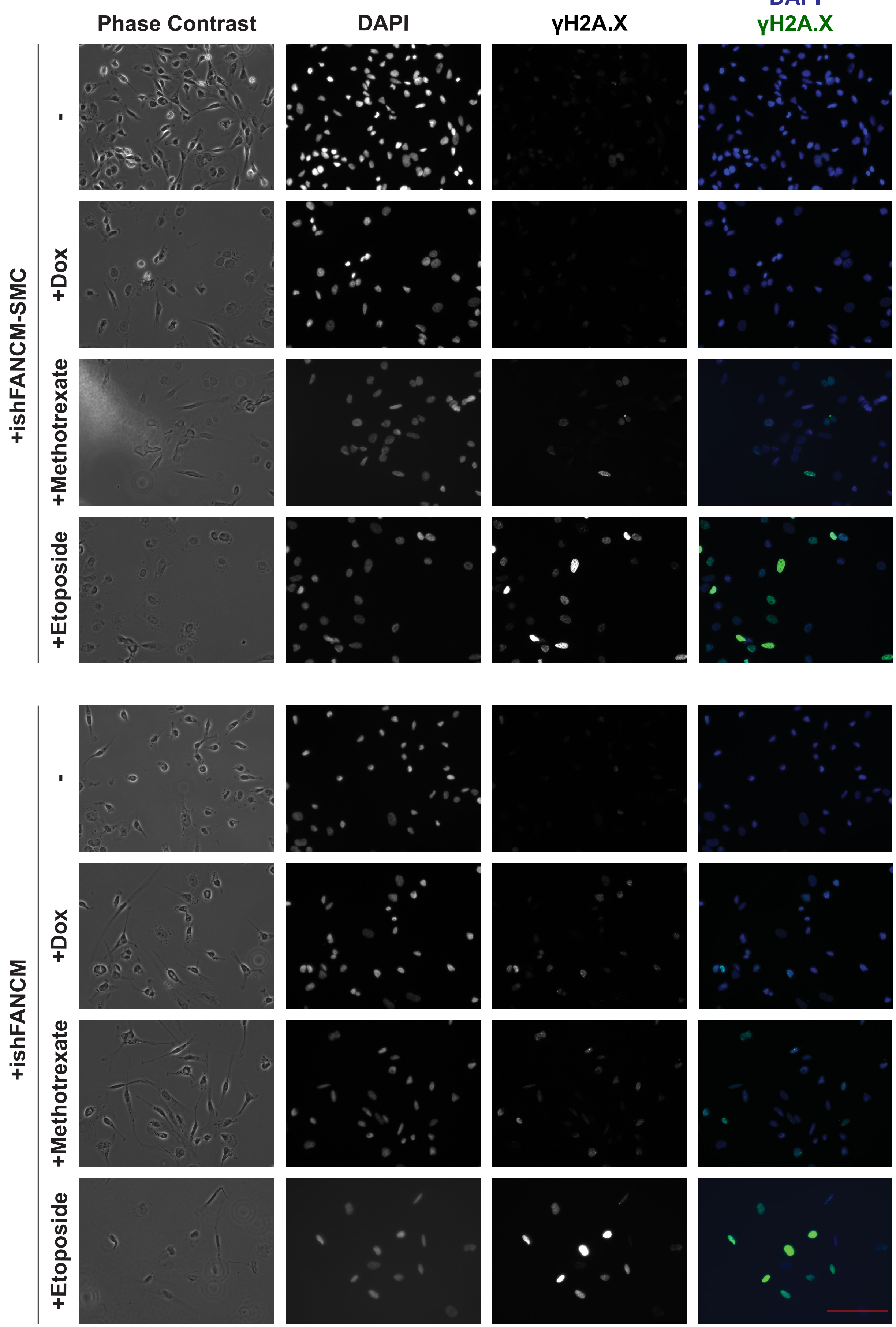

Figure S8. Representative γH2A.X staining used for DNA damage quantification, related to Figure 5.
Representative phase-contrast, DAPI, γH2A.X, and merged images from Rh4 cells expressing inducible shFANCM or seed-matched control. Cells were treated with doxycycline, vehicle, methotrexate, or etoposide as indicated before fixation and imaging. Scale bars, 50 µm.

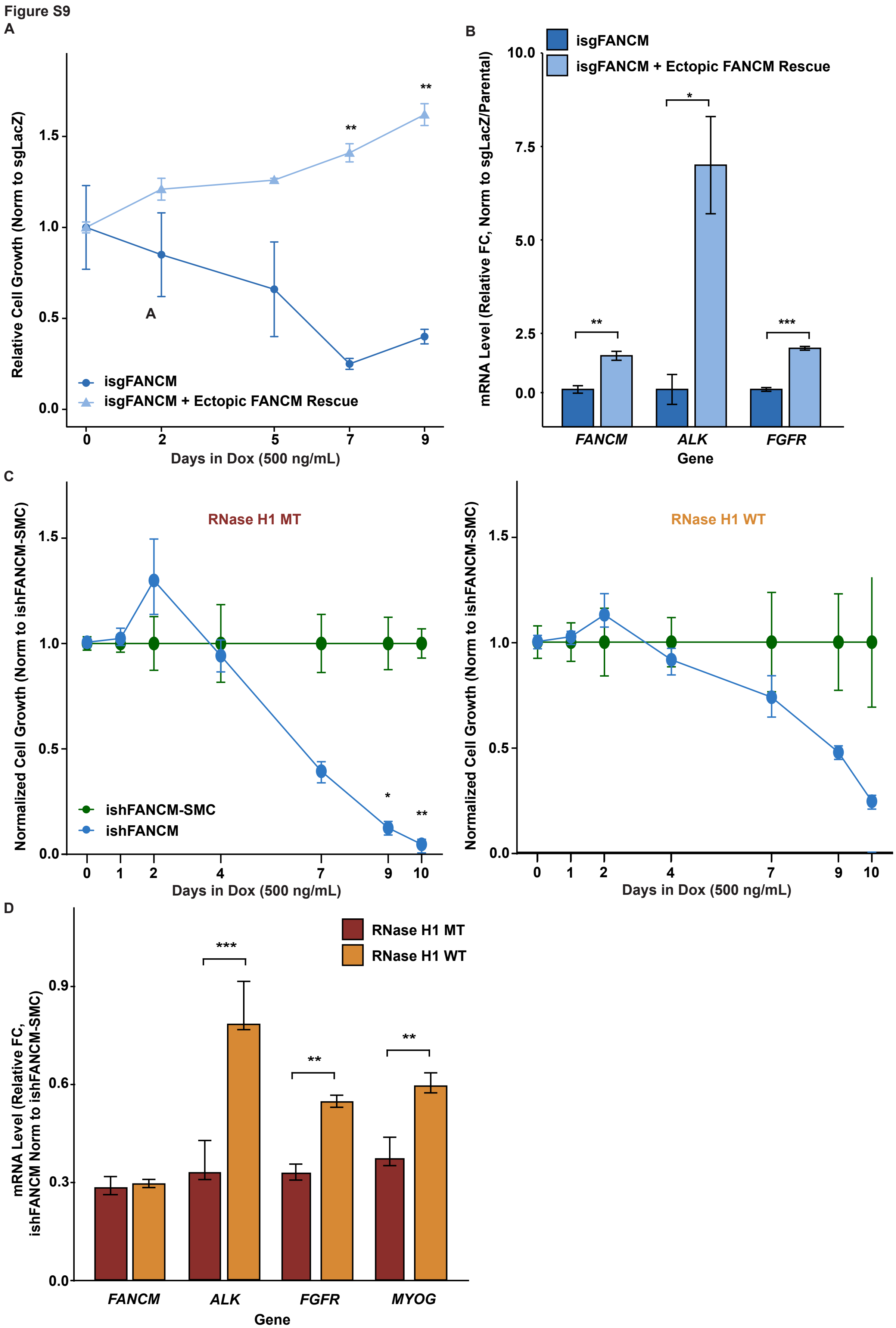

Figure S9. *FANCM* and *RNase H1* rescue analyses, related to Figure 5.
(A) Growth rescue after ectopic sgRNA-resistant *FANCM* expression in the setting of inducible sgFANCM knockout. Data are represented as mean ± SEM. ** indicates $p < 0.01$ by two-way ANOVA with Sidak's multiple-comparisons test.
(B) RT-qPCR analysis of selected PAX3::FOXO1-regulated transcripts after *FANCM* rescue in the setting of *FANCM* knockout. Data are represented as mean ± SEM. *, **, and *** indicate $p < 0.05$, $p < 0.01$, and $p < 0.001$, respectively, by unpaired two-sided t tests.
(C) Growth curves after inducible shFANCM or seed-matched control in cells expressing catalytically inactive mutant *RNase H1* or wild-type *RNase H1*. Data are represented as mean ± SEM. * and ** indicate $p < 0.05$ and $p < 0.01$, respectively, by two-way ANOVA with Sidak's multiple-comparisons test.
(D) RT-qPCR analysis of selected PAX3::FOXO1-regulated transcripts in *RNase H1* mutant or wild-type rescue conditions after *FANCM* KD. Data are represented as mean ± SEM. ** and *** indicate $p < 0.01$ and $p < 0.001$, respectively, by unpaired two-sided t tests.

Figure S10

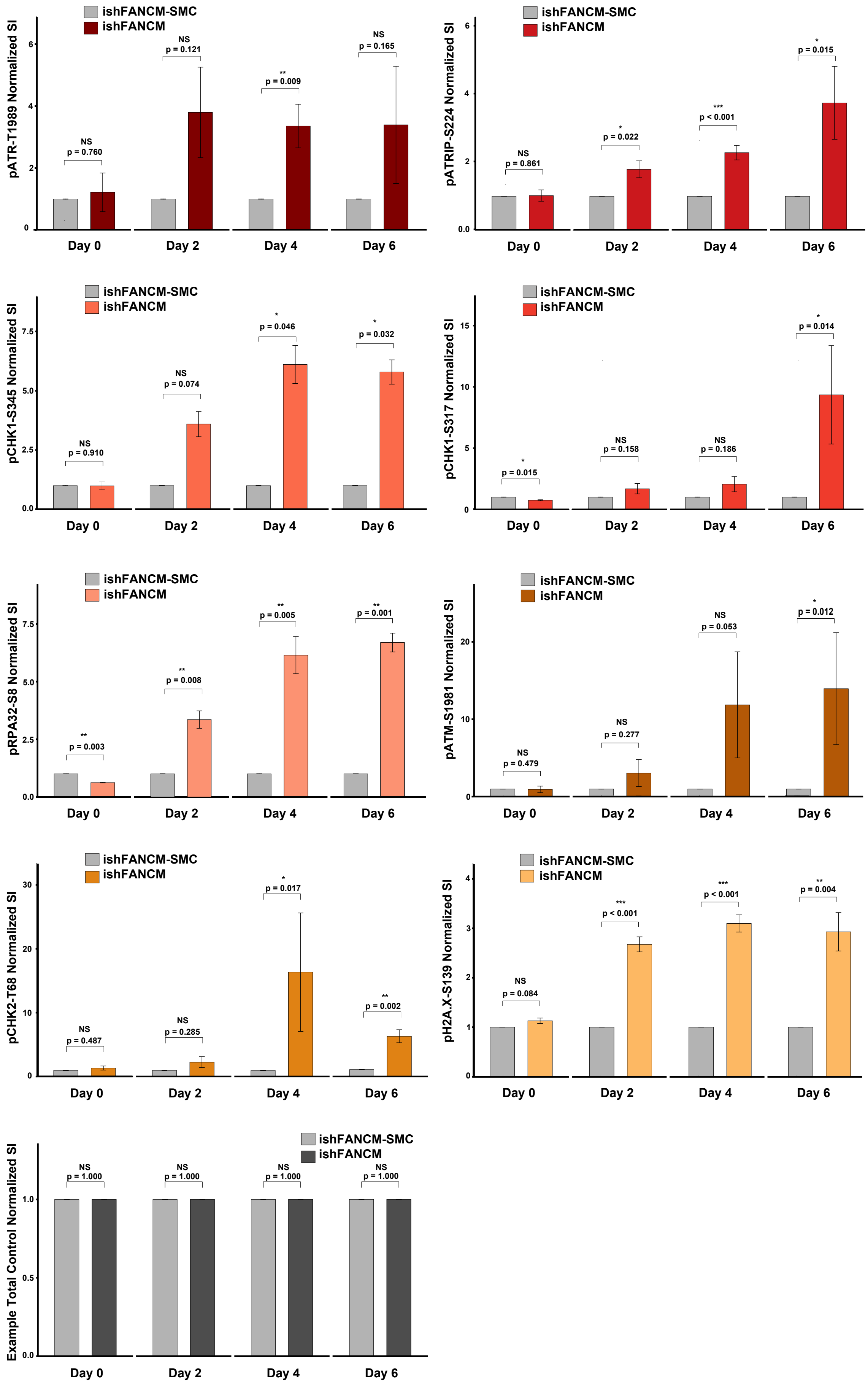

Figure S10. Quantification of replication stress, DNA damage, and DDR signaling markers after *FANCM* KD, related to Figure 5. Densitometric quantification of pATR-T1989, pATRIP-S224, pCHK1-S317, pCHK1-S345, pRPA32-S8, pATM-S1981, pCHK2-T68, γH2A.X-S139, and representative total protein control signals across the Western blot time courses in **Figure 5A** and **Figure 5D**. Signal intensities were normalized to the corresponding total protein control and then to the day-matched seed-matched control. Data are represented as mean ± SEM. *, **, and *** indicate $p < 0.05$, $p < 0.01$, and $p < 0.001$, respectively, and NS indicates not significant, by unpaired two-sided t tests.

Figure S11

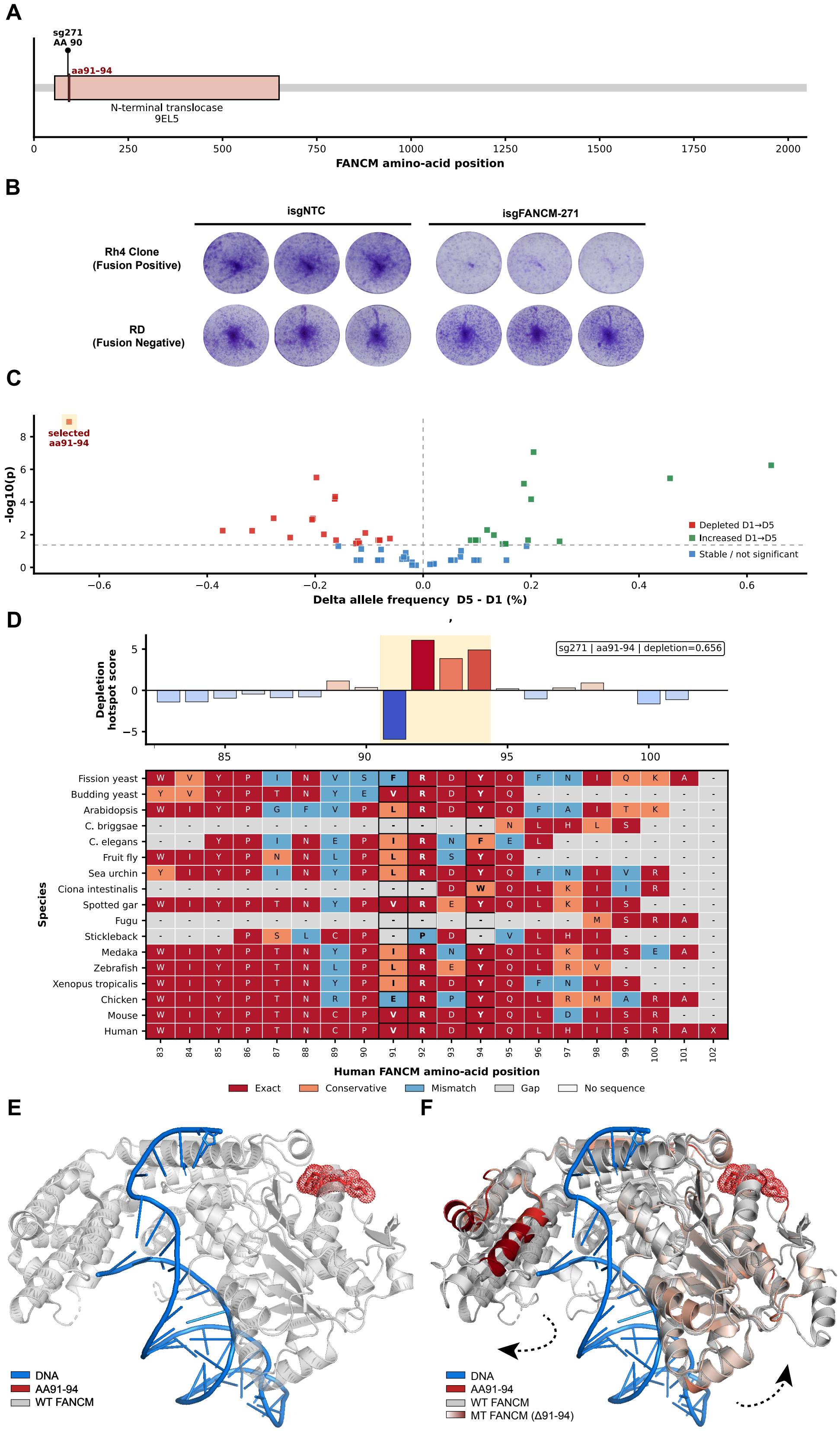

Figure S11. Focused validation of an N-terminal FANCM helicase hotspot nominated by exon tiling, related to Figure 7.

(A) Schematic of FANCM showing the selected N-terminal hotspot-targeting sgRNA and the local deleted amino-acid interval.

(B) Clonogenic validation of the selected hotspot-targeting sgRNA in FP Rh4 Clone and FN RD cells.

(C) Allele-level sequencing analysis showing edited allele frequency changes between day 1 and day 5. Depleted, enriched, and stable alleles are indicated.

(D) Local depletion hotspot score and conservation alignment across the N-terminal FANCM region surrounding residues 91 to 94.

(E) Structural mapping of the selected depleted in-frame allele onto the N-terminal FANCM domain.

(F) Predicted structural effect of the Δ91-94 allele compared with the wild-type FANCM domain. Mutant FANCM is colored in a gradient according to the intensity of the predicted amino acid atomic coordinate shifts relative to the FANCM WT reference structure.

Figure S12

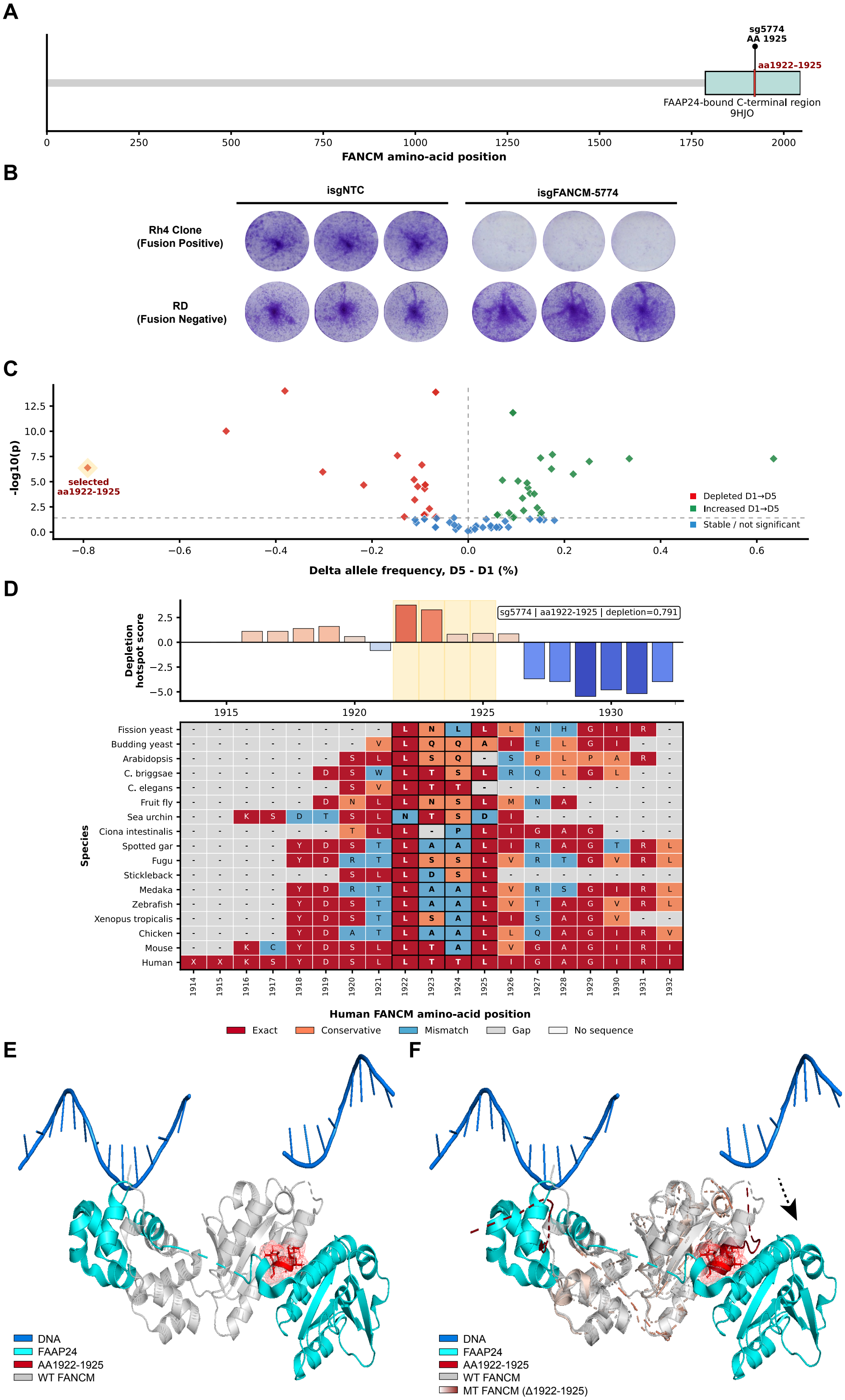

Figure S12. Focused validation of a C-terminal FANCM FAAP24-interacting hotspot nominated by exon tiling, related to Figure 7.

(A) Schematic of FANCM showing the selected C-terminal hotspot-targeting sgRNA and the local deleted amino-acid interval.

(B) Clonogenic validation of the selected hotspot-targeting sgRNA in FP Rh4 Clone and FN RD cells.

(C) Allele-level sequencing analysis showing edited allele frequency changes between day 1 and day 5. Depleted, enriched, and stable alleles are indicated.

(D) Local depletion hotspot score and conservation alignment across the C-terminal FANCM region surrounding residues 1922 to 1925.

(E) Structural mapping of the selected depleted in-frame allele onto the FAAP24-interacting FANCM domain.

(F) Predicted structural effect of the Δ1922-1925 allele compared with the wild-type FANCM domain. Mutant FANCM is colored in a gradient according to the intensity of the predicted amino acid atomic coordinate shifts relative to the FANCM WT reference structure.

Figure S13

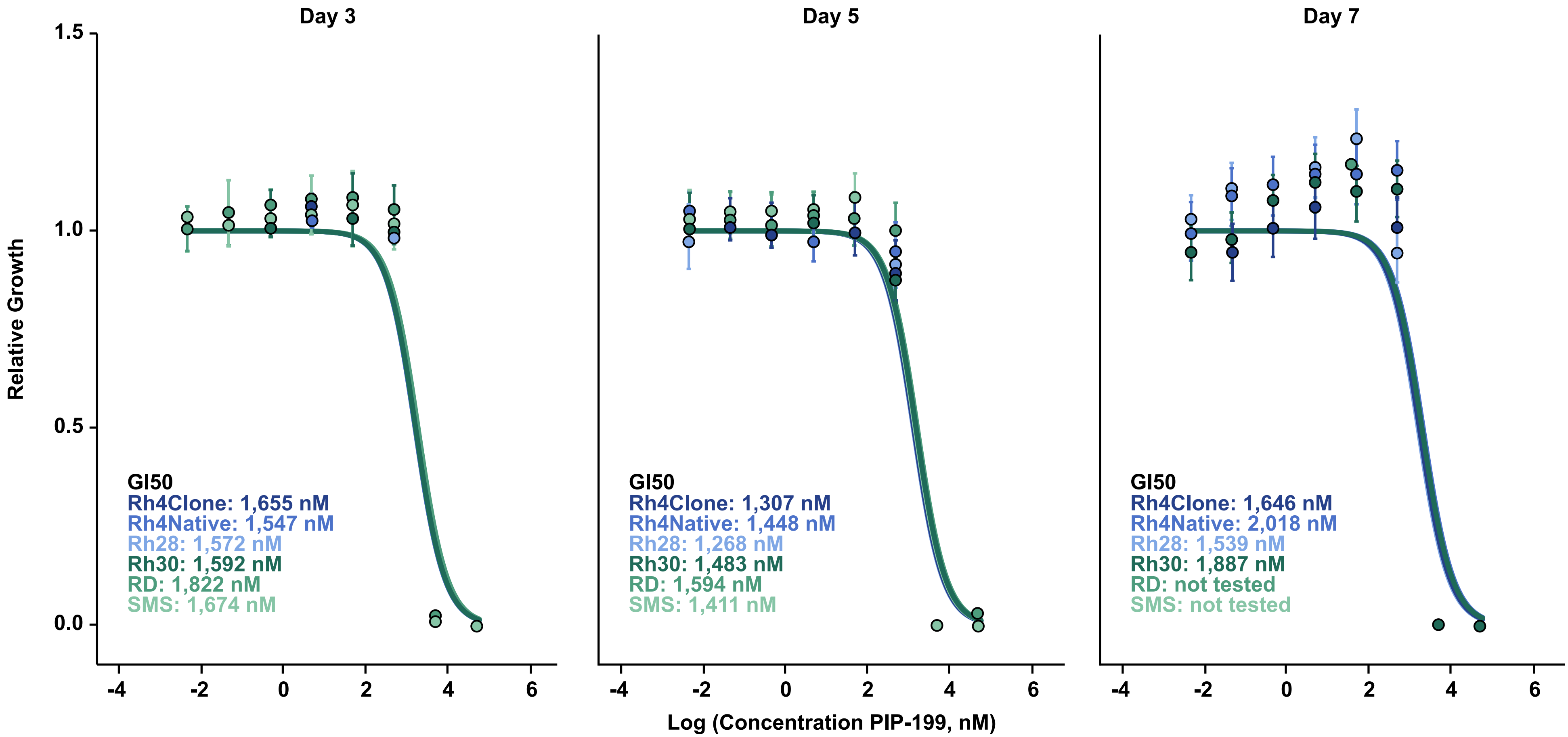

Figure S13. PIP-199 does not show FP-selective growth inhibition in RMS cell lines, related to Figure 7.
Dose-response growth assays measuring relative growth rate after PIP-199 treatment across FP RMS cell lines and FN RMS cell lines at days 3, 5, and 7. Calculated GI50 values are shown within each panel. RD and SMS-CTR were not tested at day 7 due to overproliferation. Data are represented as mean ± SEM.

Figure S14

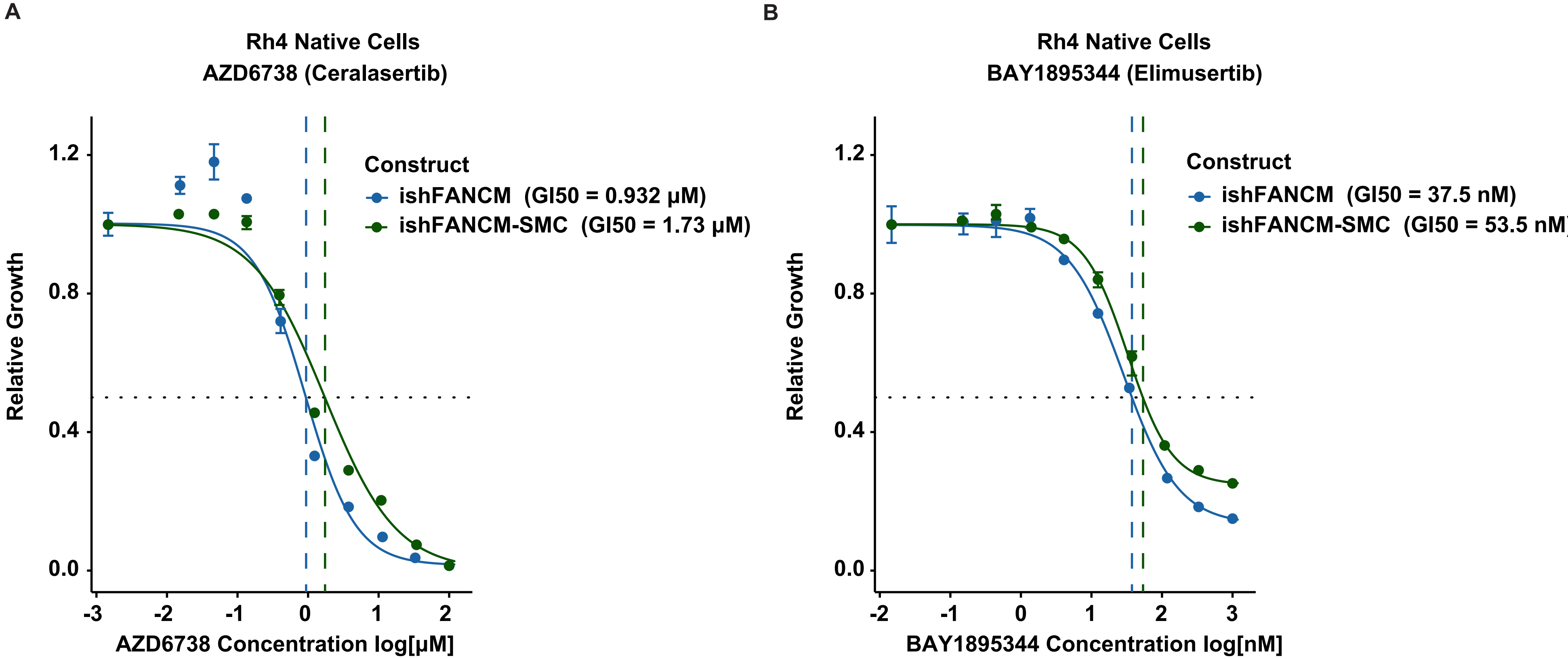

Figure S14. *FANCM* KD sensitizes FP RMS cells to ATR inhibitors, related to Figure 7.
(A) Dose-response curve for AZD6738 (Ceralasertib) in Rh4 Native cells expressing inducible shFANCM or seed-matched control. Calculated GI50 values are shown. Data are represented as mean ± SEM.
(B) Dose-response curve for BAY1895344 (Elimusertib) in Rh4 Native cells expressing inducible shFANCM or seed-matched control. Calculated GI50 values are shown. Data are represented as mean ± SEM.

Figure S15

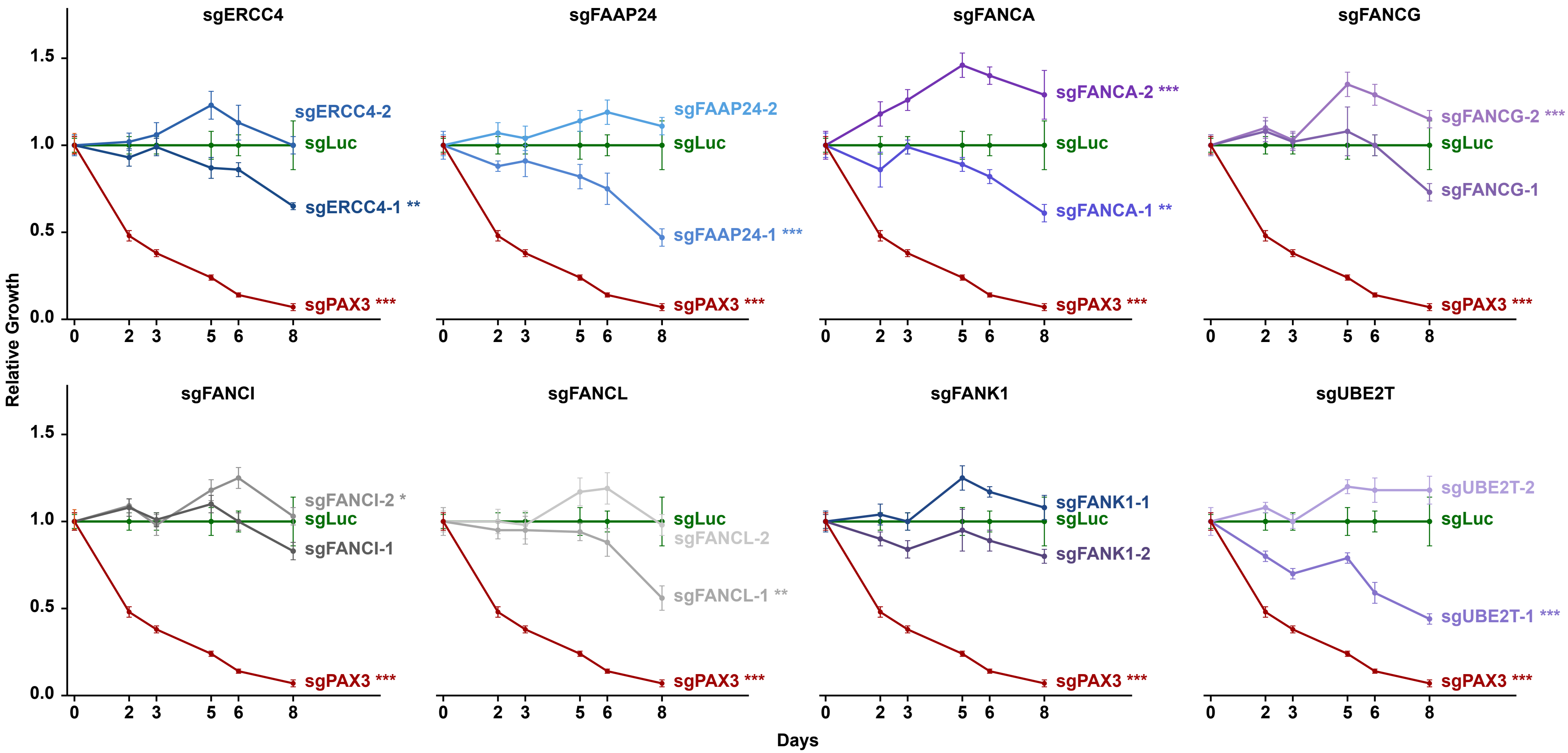

Figure S15. Selected FA pathway and FANCM-interacting genes produce partial growth defects in FP RMS cells, related to Figure 7.
Growth curves after sgRNA-mediated depletion of selected FA pathway or FANCM-interacting genes in FP RMS cells. sgLuc served as the negative control and sgPAX3 served as the positive-control dependency. Two sgRNAs were tested per target where indicated. Data are represented as mean ± SEM. *, **, and *** indicate $p < 0.05$, $p < 0.01$, and $p < 0.001$, respectively, by F tests comparing nested spline curve models with sgLuc.
